# Acoustic immune reprogramming: a novel paradigm for spatiotemporally controlled immune regulation using ultrasound-responsive nanoplatforms

**DOI:** 10.3389/fimmu.2025.1715455

**Published:** 2025-12-15

**Authors:** Tianyi Chen, Junli Chen, Mingkai Chen, Ruixiao Song, Mingsi Wang, Xiaolong Yu

**Affiliations:** 1 Science and Education Section, Wujin Hospital Affiliated With Jiangsu University, Changzhou, Jiangsu, China; 2 School of Medicine, Jiangsu University, Zhenjiang, Jiangsu, China; 3 Department of Pulmonary and Critical Care Medicine, The First Affiliated Hospital of Soochow University, Suzhou, Jiangsu, China; 4 School of Medicine, Southeast University, Nanjing, Jiangsu, China; 5 Department of Orthopaedics, Zhongda Hospital Affiliated to Southeast University, Nanjing, Jiangsu, China; 6 Center of Interventional Radiology and Vascular Surgery, Nurturing Center of Jiangsu Province for State Laboratory of AI Imaging and Interventional Radiology, Department of Radiology, Zhongda Hospital, Medical School, Southeast University, Nanjing, China

**Keywords:** acoustic immune reprogramming, ultrasound-responsive nanoplatforms, immune modulation, spatiotemporal control, immunotherapy

## Abstract

The maintenance of immune homeostasis is a cornerstone of health, and mastering its regulation is now the focus of groundbreaking disease treatments. This review innovatively proposes and elucidates the “Acoustic Immune Reprogramming” framework: using ultrasound as a catalyst and nanoplatforms as carriers to enable hierarchical, spatiotemporally precise immune microenvironment interventions. It represents an advanced form of immunomodulation distinguished by its capacity for physical programming—the proactive and rational remodeling of cellular functions and tissue microecology with spatiotemporal precision and dose control unavailable to molecular agents alone. The review covers key technological advances in acoustic-mediated biological barrier penetration, local microenvironment programming, and precise delivery of drugs/genes/antibodies. It highlights functional remodeling of macrophages, neutrophils, dendritic cells (DCs), and the synergistic network effect. From a systems engineering perspective, acoustic nanoplatforms offer remote physical modulation, non-invasive activation, spatiotemporal control, and integrate bioinformatics, materials science, medical engineering, and AI. Three challenges are identified: (1) deciphering “black box” mechanisms via acoustic immune biology and single-cell multi-omics; (2) calibrating biological acoustic dosimetry for “physical input-to-immune effect” translation, plus personalized treatment prediction via cavitation standardization/digital twinning; (3) managing safety boundaries for “immune-programmable” nanomaterials and intelligent closed-loop systems. This review establishes the acoustic immune reprogramming framework, providing a theoretical basis for immune modulation/precision medicine and guiding interdisciplinary breakthroughs. Future advances may make acoustic “spatiotemporal immune sculpting” a cornerstone of intelligent medicine.

## 1 Introduction

Immune homeostasis serves as the cornerstone for maintaining overall health. Dysregulation in specific tissues or organs constitutes a common pathological basis for many significant diseases. These diseases, ranging from the suppressive “cold” state in the tumor microenvironment (TME) (1) to the destructive autoimmune attacks seen in rheumatoid arthritis (RA) (2), from sterile inflammation in atherosclerotic plaques (3) to the uncontrolled mucosal immune responses characteristic of inflammatory bowel disease (IBD) (4), and including immune-privileged regions affected by the blood-brain barrier (BBB) in neurodegenerative disorders like Alzheimer’s disease (5), all share a fundamental issue: the disruption of immune balance within specific microenvironments.

Despite significant advances in treatment strategies for these diseases, a common fundamental challenge persists: the lack of spatiotemporal precision. Traditional small-molecule drugs, along with biologics such as monoclonal antibodies, often rely on systemic administration, which inevitably results in drug distribution to non-target tissues, triggering off-target (6–8) effects and limiting the maximum effective concentration that can be achieved at the site of the lesion (9, 10). Even modern immunotherapies, exemplified by immune checkpoint inhibitors (ICIs), while mobilizing systemic anti-tumor immunity, frequently lead to severe immune-related adverse events (11, 12). The challenges are more pronounced in specific domains: in cardiovascular medicine, statins effectively control blood lipids but fail to address the residual inflammatory risks associated with plaques (13); in neuroscience, the physiological barrier posed by the BBB prevents most potential therapeutic agents from effectively entering the central nervous system (14); and in the treatment of IBD, the low bioavailability of oral medications and the limitations of localized enema therapies similarly constrain therapeutic efficacy (15).

These challenges underscore a critical unmet clinical need: the development of a therapeutic paradigm capable of precisely controlling the local microenvironment—at the right time, in the right location, and at the optimal dosage. In this context, ultrasound-responsive drug delivery systems (DDS) have emerged as a promising solution. The unique capability of ultrasound to serve as both a real-time imaging modality and a therapeutic trigger provides an unparalleled closed-loop system for spatiotemporally controlled interventions (16). This theranostic (therapy + diagnostic) interface allows for visualizing the target, guiding the energy deposition, and even monitoring the treatment effect in real-time, which is a fundamental advantage over other physical energy sources. Leveraging this capability, researchers have developed a range of intelligent nano- and microcarriers that undergo physical or chemical changes under ultrasound stimulation. These carriers have evolved from early drug-loaded microbubbles to today’s more complex platforms, including liposomes, polymeric nanoparticles, and phase-transition nanoemulsions. Their primary advantage lies in “locking” the drug’s activity within the carrier until it reaches the target site, where external ultrasound triggers its “unlocking”. This “on-demand release” strategy offers unprecedented opportunities for achieving efficient, low-toxicity drug delivery (17–19).

In recent years, the utilization of ultrasound and its mediated drug delivery for regulating the immune system—termed “ultrasound-mediated immunomodulation”—has emerged as a rapidly developing interdisciplinary frontier (20, 21). Numerous studies have shown that ultrasound can modulate immune status by inducing immunogenic cell death (ICD) through thermal and mechanical effects or by enhancing the permeation of drugs and cells. However, there is a notable absence of a unified and theoretically robust nomenclature in academia to address the complex, multi-layered biological effects involving multi-scale physical energy deposition, diverse biological carrier platforms, and polyfunctional cell conversion. To address this gap, this review systematically introduces and defines the novel concept of “acoustic immune reprogramming,” which aims to serve as a cohesive theoretical framework encompassing all strategies and applications that utilize acoustic energy for the functional programming or reprogramming of immune and stromal cells in specific pathological microenvironments under spatiotemporal control.

**Figure 1:**
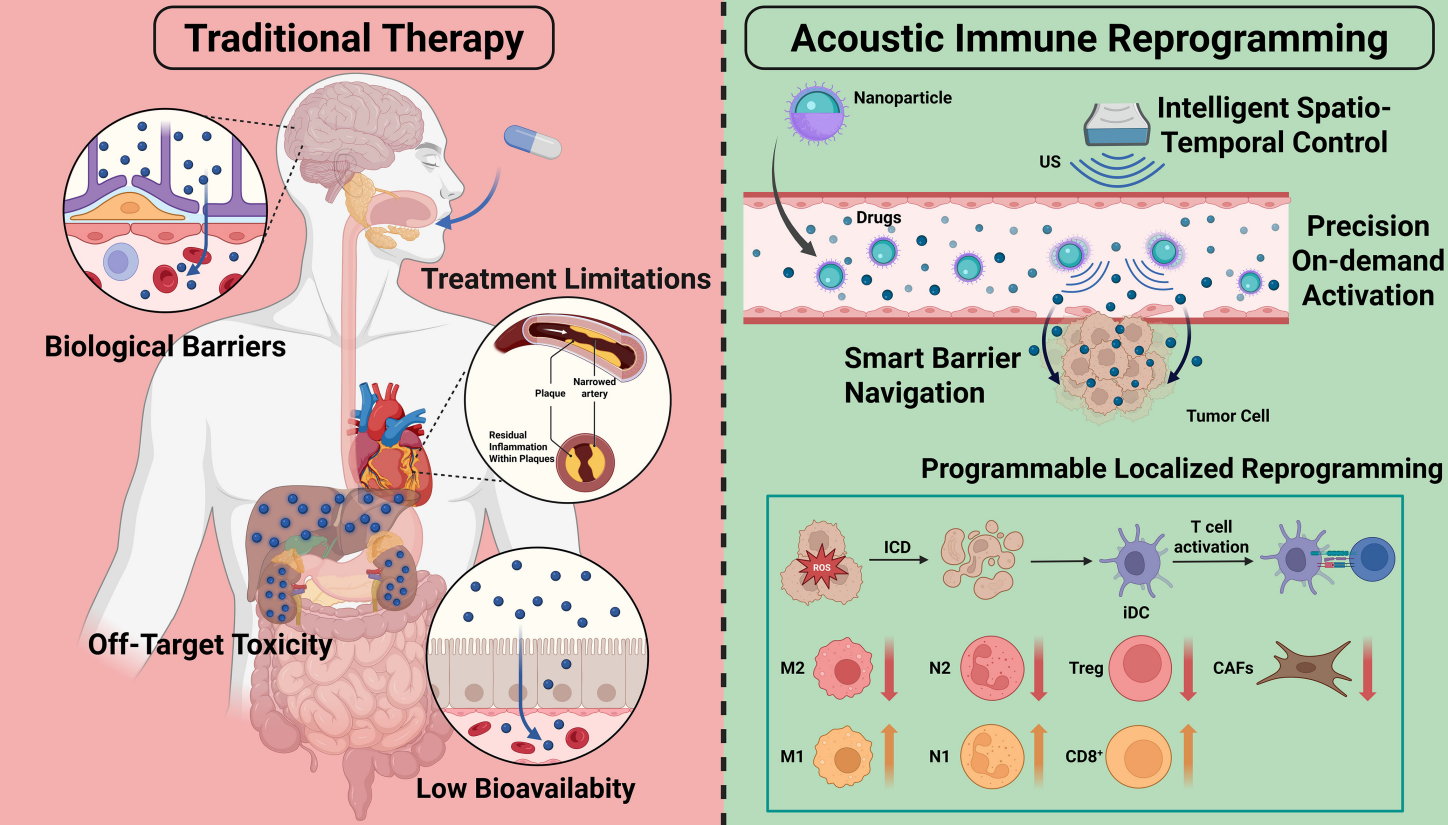
Definition of paradigm: the central concept of acoustic immune remodeling (Created in https://BioRender.com): Traditional therapies encounter significant challenges, including off-target toxicity (8), biological barriers (14), treatment limitations (13), and low bioavailability (15). Acoustic immune remodeling seeks to employ ultrasound to induce ICD through thermal and mechanical effects, or to modulate immune status by enhancing the permeability of drugs and cells (22). This approach offers several advantages, including “spatio-temporal control”, “on-demand activation”, “barrier breach”, and “localized reprogramming”.

We contend that the concept of “acoustic immune reprogramming” more precisely encapsulates the core essence of this paradigm: it is not merely “modulation”, but an active and profound “programming” and “reprogramming” of cellular functions and tissue microenvironments in line with predefined objectives(**Figure 1**). To provide a clear roadmap for this review, we will first delineate the technological pillars of this paradigm, namely the programmable physical energy of ultrasound and the diverse classes of ultrasound-responsive nanoplatforms. We will then systematically dissect the core strategy of acoustic immune reprogramming—cell-specific functional programming—with a focused examination of its application across key immune players (including macrophages, T cells, DCs, and neutrophils) and stromal components in the context of cancer, autoimmune diseases, and cardiovascular conditions. Finally, the review will critically address the pivotal challenges and future directions, encompassing the decoding of underlying mechanisms, the establishment of precise bio-acoustic dosimetry, and the navigation of safety thresholds, which are essential for the clinical translation of this innovative approach. This review will systematically explore the application potential of this versatile platform technology.

## 2 Enabling technologies and platforms for “acoustic immune reprogramming”

Acoustic immune remodeling relies on two distinct but complementary, independently tunable components: ultrasound, a physical energy field with intrinsic immunoregulatory effects; and ultrasound-responsive platforms (URPs), engineered carriers that enhance, localize, or diversify these effects. Notably, ultrasound alone can directly induce immune remodeling via its unique biophysical actions (mechanical, thermal, and neuromodulatory). The incorporation of URPs, however, substantially improves outcomes by overcoming biological barriers, enabling targeted delivery, and producing synergistic therapeutic effects (21, 23). This chapter systematically delineates the technical characteristics of these components and their mechanisms of action in immune remodeling.

### 2.1 Ultrasound: the fundamental physical force behind immune reprogramming

Ultrasound-mediated remodeling of the immune microenvironment arises from complex biophysical effects generated during tissue interactions. The relationship between acoustic parameters and biological outcomes is highly nonlinear, with effect type determined by the interplay of intensity, frequency, duty cycle, and pulse duration rather than intensity alone (23, 24). Understanding these parameter-effect relationships is essential for rationally designing acoustic immunomodulation strategies.

Ultrasound generates biological effects through two principal physical mechanisms. Mechanical effects originate from acoustic radiation force, cavitation, and acoustic streaming (25–27). Cavitation, defined as the oscillation, growth, and potential collapse of gas nuclei under acoustic fields, represents the dominant mechanism driving URP activity. Based on bubble dynamics, cavitation bifurcates into stable cavitation, characterized by sustained oscillation without collapse, and inertial cavitation, involving violent bubble implosion generating extreme local temperatures and pressures (27, 28). Thermal effects, in contrast, result from continuous absorption of acoustic energy converting to heat, typically requiring sustained high-intensity exposure with duty cycles exceeding 10% or continuous wave delivery (23, 29, 30).

Critically, the intensity-effect relationship defies simple categorization. Low-intensity pulsed ultrasound, operating at spatial-peak temporal-average intensities below 1 W/cm², induces stable cavitation when combined with microbubbles at mechanical index values of 0.3-0.6, generating microstreaming and reversible membrane sonoporation without significant thermal deposition (26, 31, 32). Conversely, high-intensity ultrasound employing peak pressures exceeding 10 MPa can produce predominantly mechanical rather than thermal effects when delivered in short pulses with duty cycles below 1-2%. This regime, exemplified by histotripsy using microsecond pulses and boiling histotripsy (BH) employing millisecond pulses, mechanically disintegrates tissue through inertial cavitation without thermal accumulation (30, 33, 34). The apparent paradox—that high intensity does not inherently favor thermal over mechanical effects—resolves when recognizing duty cycle as the critical determinant. Continuous or high-duty-cycle delivery permits thermal accumulation, achieving temperatures of 60-80°C in high-intensity focused ultrasound (HIFU) thermal ablation, whereas pulsed delivery with low duty cycles dissipates heat between pulses, favoring mechanical disruption (23, 29, 35, 36).

The cavitation threshold, defined as the minimal acoustic pressure required to initiate bubble oscillation, exhibits strong frequency dependence and tissue-specific variation. In the absence of exogenous nucleation sites, intrinsic cavitation thresholds range from 15 to 30 MPa at megahertz frequencies. The introduction of microbubbles dramatically reduces this threshold to 0.3-0.6 MPa, enabling mechanical effects at clinically safe intensities (32, 37). This threshold reduction underlies the synergistic enhancement observed when combining ultrasound with URPs.

Within the context of acoustic immune reprogramming, ultrasound functions as a precisely programmable multi-parameter physical energy field rather than a singular tool. By systematically modulating frequency, which governs penetration depth and spatial resolution; acoustic pressure, which determines cavitation intensity; pulse sequence and duty cycle, which control the balance between mechanical and thermal effects; and exposure time, which dictates total energy deposition, a spectrum of biological effects spanning mild membrane perturbations to complete tissue ablation can be achieved (38). This parametric versatility permits selective activation of distinct immune reprogramming pathways tailored to specific therapeutic objectives, transforming ultrasound from a simple triggering mechanism into a multifunctional immunomodulatory controller.

Based on fundamental physical effects and desired immunological outcomes, the mode of ultrasound action categorizes into three primary regulatory pathways.

1. Barrier Permeability and Enhanced Immune Infiltration: This approach aims to physically overcome tissue barriers (such as vascular endothelium and dense tumor stroma) to facilitate the infiltration of immune cells and therapeutic agents. This is typically achieved through the use of low-intensity pulsed ultrasound (LIPU) combined with microbubbles. Under these conditions, the microbubbles undergo stable, non-destructive oscillations, generating micro-streams and radiation forces that temporarily and reversibly open tight junctions between endothelial cells, a process known as “sonoporation” (39, 40). Sonoporation enhances liposome accumulation and penetration in tumors with low enhanced permeability and retention(EPR) (41). A study on pancreatic cancer shows a median survival increase from 8.9 months to 17.6 months in ten subjects augmented with sonoporation compared to 63 historical controls (42). In this context, ultrasound acts as a path-clearing agent, eliminating barriers for subsequent immune attacks.
2. Immunostimulatory *In Situ* Ablation: This approach aims to convert the *in situ* tumor into an endogenous vaccine through the application of high-intensity ultrasound energy. It is primarily realized using two techniques: HIFU thermal ablation and BH mechanical ablation. The high temperatures associated with HIFU (>60°C) induce coagulative necrosis in tumor cells, whereas BH employs intense cavitation effects to mechanically liquefy tissue (30). Both methods result in the release of significant amounts of tumor-associated antigens (TAAs) and damage-associated molecular patterns (DAMPs), thereby initiating a robust T-cell-mediated systemic anti-tumor immune response that effectively inhibits the growth of distant metastatic tumors, referred to as the “abscopal effect” (43). This strategy offers a powerful approach to achieving *in-situ* personalized immunization, effectively turning the tumor into an endogenous vaccine.
3. Targeted Neuro-Immune Axis Regulation: This is one of the most advanced fields in recent years, aiming to non-invasively and precisely modulate peripheral nerves using ultrasound, thereby indirectly regulating systemic immune responses. This is typically achieved with low-intensity, low-frequency focused ultrasound in a non-thermal, non-destructive manner. A key mechanism involves targeting the cholinergic anti-inflammatory pathway, which stimulates the vagus nerve to release acetylcholine at the peripheral terminals of the spleen, inhibiting macrophage production of pro-inflammatory cytokines such as TNF-α (44). The response of excitatory and inhibitory neurons to ultrasound pulse repetition frequency (PRF) differs inherently. By modulating the PRF of transcranial focused ultrasound (tFUS), specific types of neurons can be non-invasively targeted (45). Low-intensity tFUS significantly reduces excitatory neuron expression while increasing that of inhibitory GABAergic neurons. The PIEZO-1 protein in GABAergic neurons plays a role in inhibitory neuromodulation (46). Graham M. Seasons and colleagues propose that the heat shock response (HSR) may serve as an upstream regulatory factor in ultrasound’s anti-neuroinflammatory effects (47). Additionally, breakthrough studies have shown that ultrasound can prevent renal ischemia-reperfusion injury through the splenic cholinergic anti-inflammatory pathway and the α7 nicotinic acetylcholine receptor (α7nAChR) (48). This acoustic neuromodulation offers a novel, drug-free therapeutic approach for treating systemic inflammation or autoimmune diseases, transforming ultrasound into a non-invasive biotechnological scalpel that can fundamentally reprogram neuro-immune communication.

#### 2.1.1 Ultrasound’s independent immunomodulatory capacity and synergistic enhancement by platforms

Notably, the three ultrasound modulation modes described above can independently induce immune remodeling without exogenous agents; this agent−free capability arises from biophysical effects generated by direct ultrasound–tissue interactions. When combined with engineered URPs, these effects can be substantially amplified, spatially localized, or functionally extended. For example, in regulating barrier permeability, ultrasound alone can disrupt endothelial tight junctions via mechanical stress, whereas the introduction of microbubbles can reduce the acoustic pressure threshold required to safely open the blood–brain barrier from >1 MPa to approximately 0.3–0.6 MPa and confer improved spatiotemporal precision. In immunogenic ablation, HIFU thermal ablation can itself induce ICD, while co−administration with thermosensitive liposomes enables synchronized release of chemotherapeutic agents at the periphery of the ablation zone, producing a “physical ablation + chemical cytotoxicity + immune activation” triple−strike effect. Thus, URPs serve primarily as “acoustic effect amplifiers” and “therapeutic payload carriers” rather than prerequisites for ultrasound−mediated immunomodulation.

**Figure 2:**
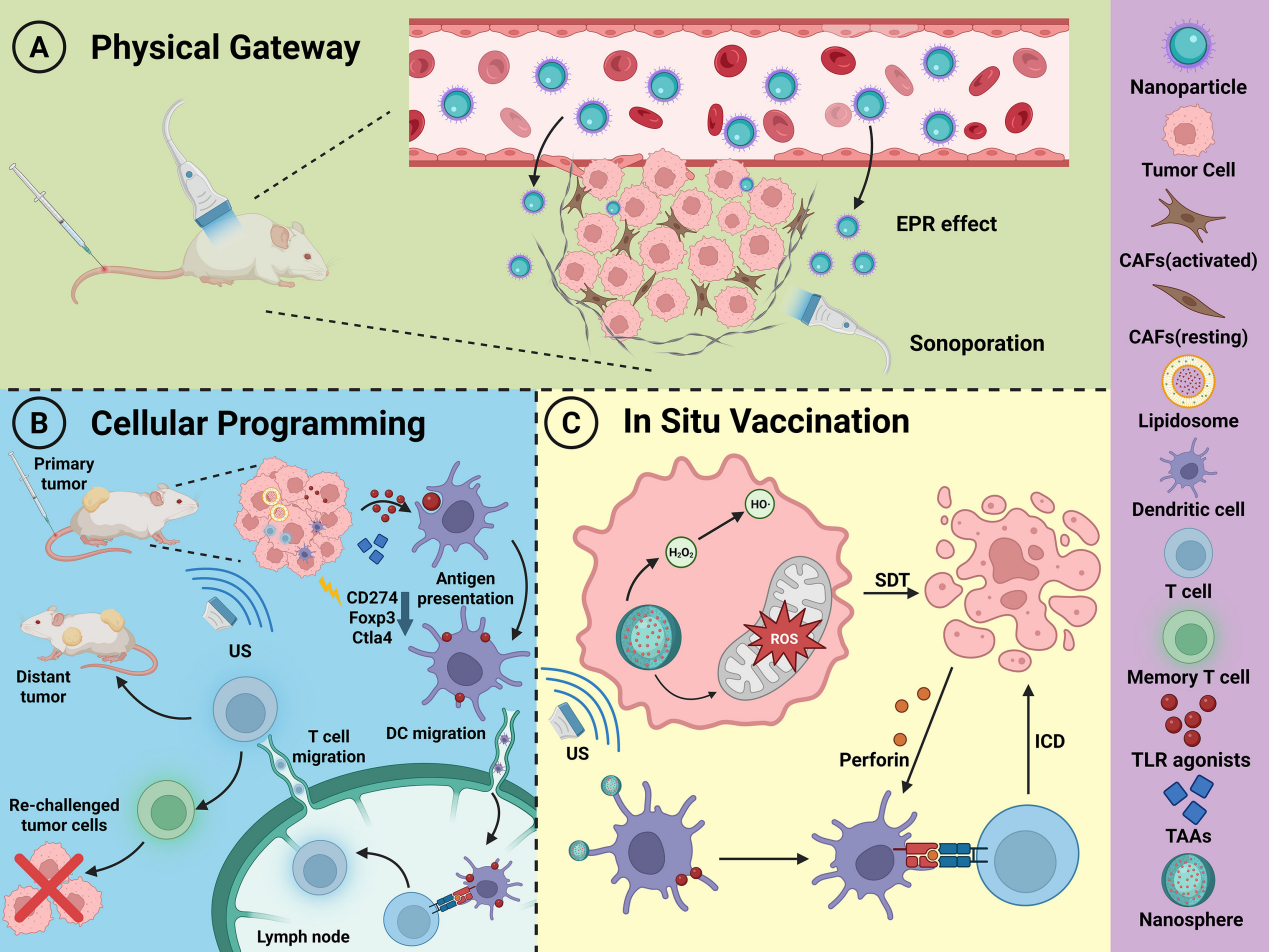
Multifunctional roles of ultrasound in acoustic immune reprogramming (Created in https://BioRender.com): **(A)** Physical Gateway: Ultrasound, in combination with microbubbles, temporarily and reversibly opens biological barriers (e.g., vascular endothelium, BBB) through sonoporation, enhancing the infiltration of immune cells and therapeutic agents (61). **(B)** Cellular Programming: Low-intensity focused ultrasound (LIFU) can precisely modulate specific cell functions, such as reprogramming neuronal activity to regulate systemic immunity via the neuro-immune axis (44, 48) or directly enhancing T cell activation through mechanostimulation (62). **(C)***In Situ* Vaccination: High-intensity ultrasound ablation (e.g., HIFU) induces ICD, releasing tumor antigens and danger signals to initiate a systemic anti-tumor immune response akin to an endogenous vaccine (63).

The programmability of ultrasound offers considerable flexibility for its application in immune reprogramming. By precisely designing its physical parameters, researchers can selectively activate specific biological pathways. As illustrated in **Figure 2**, these pathways can be conceptually summarized as: (A) Physical Gateway, primarily through sonoporation to breach barriers; (B) *In Situ* Vaccination, via ablative techniques to generate endogenous vaccines; and (C) Cellular Programming, leveraging neuromodulation and other precise effects to directly alter cell phenotypes and functions. This multifaceted controllability provides robust and versatile technical support for acoustic immune reprogramming.

However, this flexibility necessitates careful parameter optimization, as different parameter combinations can lead to substantially different immune outcomes. For instance, while HIFU thermal ablation (>60°C) effectively induces ICD for *in situ* vaccination, excessively high temperatures may destroy antigens and dampen immune responses (30). Conversely, LIPU combined with microbubbles achieves immunomodulation through mechanical rather than thermal effects, making parameter selection critical for balancing efficacy and safety. **Table 1** systematically summarizes the correlation between ultrasound parameters and immune effects across various disease scenarios, providing theoretical guidance for clinical translation. Future clinical translation will benefit from standardized parameter reporting and the development of patient-specific acoustic dosing strategies based on individual tissue characteristics and therapeutic objectives.

**Table 1:** Optimal ultrasound parameters for immune reprogramming in different disease contexts.

Disease context	Therapeutic goal	Ultrasound mode	Parameter ranges	Immune effects	References
Solid Tumors	ICD induction and *in situ* vaccination	HIFU	Intensity: 100–1000 W/cm² Frequency: 1–3 MHz Pulse duration: 10–100 ms	TAAs/DAMPs release; DC activation; T-cell priming	(30, 43, 49)
Solid Tumors	Enhanced drug delivery & immune cell infiltration	LIPU + Microbubbles	MI: 0.4-1.2 PRF: 1–100 Hz Duty cycle: 1-20%	Sonoporation; Vascular permeability; Immune cell trafficking	(39–42, 50)
Autoimmune Diseases	Anti-inflammatory macrophage polarization	LIPU + Nanocarriers	Intensity: 0.5–3 W/cm² Frequency:0.5-1.5 MHz Exposure time: 5–15 min	M1→M2 conversion; TNF-α/IL-6 reduction; Treg induction	(51–53)
Neurological Disorders	Neuro-immune modulation	tFUS	Intensity: 0.5–30 W/cm² Frequency: 0.25-0.65 MHz PRF: 10–1000 Hz	Vagus nerve activation; Acetylcholine release; Macrophage suppression	(36, 45, 46, 54)
Atherosclerosis	Plaque stabilization and inflammation resolution	UTMD + Microbubbles	MI: 0.8-1.6; PRF: 0.5–2 Hz	Macrophage phenotype modulation; Cytokine reduction; Fibrous cap enhancement	(55–57)
Organ Transplantation	Local immunosuppression	UTMD + Drug-loaded microbubbles	Intensity: 1–4 W/cm² MI: 0.8-1.4; Pulse length: 100–1000 cycles	Local drug concentration; T-cell inhibition; Graft survival extension	(58–60)

Parameter ranges represent typical values from preclinical and clinical studies and may require individual optimization. Immune effects are often synergistic with delivered therapeutic payloads (drugs, genes, cytokines). Abbreviations: HIFU: High-Intensity Focused Ultrasound; LIPU: Low-Intensity Pulsed Ultrasound; PRF: Pulse repetition frequency; tFUS: Transcranial Focused Ultrasound; UTMD: Ultrasound-Targeted Microbubble Destruction; MI: Mechanical Index.

### 2.2 URPs: engineered carrier systems for enhancing acoustic immune−remodeling effects

URPs are engineered carriers that amplify, localize, or diversify ultrasound−induced biological effects. Their design flexibility governs the scope and specificity of potential therapeutic strategies. Typically composed of biocompatible materials, URPs are designed to undergo defined physicochemical changes under ultrasound—for example, structural phase transitions, transient increases in membrane permeability, sonochemical reactions, or piezoelectric responses—thereby enabling spatiotemporally controlled drug release, localized generation of reactive oxygen species (ROS), amplification and transmission of mechanical forces, or generation of electric fields. URPs span a broad size range, from micron−scale gas−core systems (microbubbles) to submicron nanocarriers (nanobubbles, liposomes, inorganic nanoparticles). Differences in size, composition, and response mechanism yield complementary—and in some cases nonredundant—profiles in biodistribution, acoustic responsiveness, and appropriate application scenarios (21, 23).

#### 2.2.1 Core carrier platforms of URPs and their acoustic response mechanisms

According to their core physical forms and acoustic response mechanisms, URPs can be grouped into four principal types (**Table 2**): gas−core systems that directly harness acoustic cavitation; phase−change droplets that combine nanoscale permeation with microscale cavitation; organic nanocarriers that respond to ultrasound−induced physicochemical changes in the local microenvironment; and inorganic nanomaterials that mediate ultrasound–bio transduction through intrinsic material properties.

**Table 2:** Comparison of characteristics and immune remodeling applications of major ultrasound-responsive nanoplatforms.

Platform type	Core Composition & size	Acoustic activation mechanism	Primary payload types	Key advantages	Main limitations	Representative immuno-modulatory applications	Reference
Microbubbles	Lipid/protein shell layer + gas core (e.g., SF_6_, C_4_F_10_) (1-10 µm).	Inertial cavitation, steady-state cavitation, acoustic perforation.	Drugs, proteins, genes, gases.	The clinical applications are well-established, featuring real-time imaging navigation and robust mechanical effects.	Poor tissue penetration, a short half-life, and restricted payload capacity.	1.Enhancing the local accumulation of immune drugs such as PD-L1 antibodies in tumors.	(108, 109)
						2.Disrupting tumor blood vessels and promoting immune cell infiltration.	(39, 40)
						3.Synergistic cavitation-induced ICD.	(110, 111)
						4.Targeted delivery of immune regulatory factors (such as IFN and siRNA).	(112, 113)
						5.Enhancing BBB permeability to achieve brain immunotherapy.	(42)
Nanobubbles	Gas core + lipid/polymer/protein shell (100–1000 nm).	Cavitation and nanoparticle bubble perforation.	Small molecule drugs, proteins, nucleic acids, etc.	Nanoscale, tumor EPR effect, and improved permeability.	Bubble stability and acoustic response parameters need to be optimized.	1.Ultrasound promotes tumor ICD and the activation of anti-tumor immunity.	(110, 114)
						2.Acoustic-controlled delivery of immune adjuvants to initiate DC recruitment.	(114, 115)
						3.Assisted delivery of CRISPR/Cas9 for the reprogramming of immune cells.	(113, 116, 117)
						4.Combined with TGF-β inhibitors to regulate the TME.	(58, 117)
						5.Combined delivery of PD-L1 antibodies to achieve synergistic immunotherapy.	(108, 109)
PCNDs	Liquid perfluorocarbon core with a shell layer (200–800 nm).	Acoustic vaporization and cavitation.	Drugs, oxygen/gas, genes, etc.	Nanoscale, controllable vaporization, and strong drug loading capacity.	The phase transition threshold is elevated, and the stability of the composition requires enhancement.	1. Delivery of oxaliplatin induces ICD and enhances tumor antigen release.	(110, 111)
						2. Assisted delivery of DNA/mRNA vaccines enhances immunogenicity.	(112)
						3. Delivery of immune adjuvants such as R837 for the modulation of the TME.	(111)
						4. Delivery of O_2_/NO and other agents to reverse tumor hypoxia and activate T cells.	(118, 119)
						5. Combined delivery of PD-L1 antibodies to enhance the immune response.	(109)
Sonosensitive polymer/liposome nanoparticles	Sonosensitive polymer/lipid self-assemblies (50–300 nm).	Sonodynamic effect and structure-change controlled release.	Sonosensitizers, drugs, immune adjuvants, nucleic acids.	High drug loading capacity, multifunctional/targeted adjustability, and good biocompatibility.	Optimizing the acoustic response mechanism is challenging, and the release kinetics are inherently complex.	1. Acoustic-controlled release of STING/cGAMP agonists to activate DCs and T cells.	(114, 115)
						2. Sonosensitizers induce tumor ICD and synergistically establish immune memory.	(110, 114)
						3. Delivery of CRISPR/Cas9 to reprogram Tregs or macrophages.	(113, 116, 117)
						4. Targeted delivery for immunosuppressive diseases.	(58, 117)
						5. Combined PD-L1 antibodies/immune adjuvants to reverse immune suppression.	(108, 109)

This table summarizes the core characteristics and applications of four major ultrasound-responsive nanoplatforms in immune regulation. Abbreviations: SF_6_, sulfur hexafluoride; C_4_F_10_, perfluorobutane; PCNDs, Phase-Change Nanodroplets; EPR, Enhanced Permeability and Retention; ADV, Acoustic Droplet Vaporization; ICD, Immunogenic Cell Death; PD-L1, Programmed Death-Ligand 1; IFN, Interferon; siRNA, Small Interfering RNA; TLR, Toll-Like Receptor; STING, Stimulator of Interferon Genes; cGAMP, Cyclic Guanosine Monophosphate-Adenosine Monophosphate; CRISPR/Cas9, Clustered Regularly Interspaced Short Palindromic Repeats/Cas9; Treg, Regulatory T cells; TGF-β, Transforming Growth Factor-β; O_2_, oxygen; NO, nitric oxide; R837, Imiquimod.

##### 2.2.1.1 Gas−core acoustic agents: fundamental distinctions between microbubbles and nanobubbles and their divergent applications

Gas−core systems are the primary platforms for eliciting strong acoustic responses. Containing gas or gas precursors, they interact directly and intensely with ultrasound and are the main mediators of sonoporation and inertial cavitation. Crucially, microbubbles and nanobubbles must be distinguished within this category because they differ fundamentally in size, biodistribution, and application context. Although both feature a gaseous core and similar shell architectures, they represent complementary, noninterchangeable platforms optimized for different therapeutic objectives. Recent systematic studies have explicitly delineated this distinction (64, 65).

1. Microbubbles (1–10 μm): intravascular acoustic amplifiers

Microbubbles, the first−generation and most extensively studied gas−core platforms, comprise a thin stabilizing shell that encloses an inert gas core (e.g., SF6 or perfluorocarbons) (66). The materials used for their shells are diverse, with the most common being lipid monolayers (such as phospholipids), proteins (such as human serum albumin), or biodegradable polymers (67). Microbubbles are defined by their size (1–10 μm), which typically exceeds that of most capillaries (≈5–10 μm), thereby confining them to the vascular lumen and preventing extravasation into the interstitium (65). This size constraint is both a limitation and an advantage: microbubbles cannot directly contact extravascular tumor cells or parenchymal tissue, yet their strict intravascular localization renders them ideal agents for vascular−targeted therapies.

Microbubbles function as contrast agents at the low acoustic pressures used for diagnostic ultrasound, whereas under therapeutic ultrasound they undergo strong volumetric oscillations at their resonance frequency (typically 1–5 MHz), producing stable (sustained) or inertial (violent collapse) cavitation (26, 27). Theoretical estimates show that, under identical excitation, a 1 μm bubble’s linear acoustic scattering cross−section exceeds that of a 200 nm nanobubble by several orders of magnitude (65). Cavitation−generated local mechanical effects—including microstreaming, microjets, and shock waves—can transiently increase cell−membrane permeability (sonoporation) and disrupt endothelial barriers. Moreover, microbubbles have a short circulation half−life (≈5–10 min) and are mainly cleared by pulmonary filtration and gas dissolution; therefore, ultrasound treatment should be applied immediately or shortly after intravenous injection (64).

Microbubbles contribute to immune remodeling through several mechanisms. They enable BBB opening: shear forces from microbubble oscillation transiently disrupt tight junctions, permitting delivery of immune−checkpoint inhibitors to intracranial tumors. They mediate vascular disruption that facilitates immune infiltration by damaging tumor endothelium. In cooperation with co−injected nanocarriers (e.g., liposomes), cavitation triggers localized intravascular drug release. Finally, sonoporation facilitates transendothelial delivery of immunomodulators, such as antibodies and cytokines. Several microbubble formulations (e.g., Definity^®^, SonoVue^®^) are FDA−approved for diagnostic imaging and are being repurposed in clinical trials for therapeutic applications.

2. Nanobubbles (<1 μm): intratumoral acoustic effectors capable of extravasation

Nanobubbles are size−optimized gas−core platforms, typically 100–800 nm in diameter. Their submicron dimensions confer two principal advantages over microbubbles (64, 68, 69). First, nanobubbles can passively extravasate through fenestrations in aberrant tumor vasculature via the EPR effect, enabling direct access to the tumor interstitium, cancer cells, and infiltrating immune cells (68, 69). Second, nanobubbles exhibit markedly prolonged circulation times (hours to days), particularly after PEGylation, which permits substantial accumulation in target tissues before ultrasound activation and thereby provides a larger, more flexible therapeutic window (64, 70).

Despite their size advantages, miniaturization imposes acoustic trade−offs. Blake−threshold theory predicts that cavitation of ~200 nm nanobubbles requires acoustic pressures much higher than those for micron−scale microbubbles (65). A recent systematic study using rigorous size fractionation and acoustic assays reported that, after removing microbubble contaminants by centrifugation or flotation, pure nanobubble suspensions produced negligible echo under diagnostic ultrasound (center frequency 7 MHz; peak negative pressure 330 kPa) (64). This suggests that acoustic signals attributed to “nanobubbles” in many reports may actually originate from small numbers of residual microbubble contaminants; consequently, strict size separation and multimodal characterization are necessary when preparing and validating nanobubbles. To address the weak response of pure nanobubbles at conventional therapeutic ultrasound frequencies, many designs incorporate phase−change cores (see Sect. 2.2.1.2) that vaporize *in situ* to form microbubbles upon ultrasound activation, thereby combining nanoscale permeability with micron−scale cavitation effects (68, 71).

Nanobubbles offer several advantages for immune remodeling. After extravasation into the tumor interstitium, they can induce ICD in cancer cells. When surface−functionalized with targeting ligands (e.g., anti−CD206 antibodies), nanobubbles can selectively deliver M1−polarizing agents to tumor−associated macrophages (TAMs). They enable intratumoral delivery of immunoadjuvants, such as TLR agonists (e.g., R837) or STING agonists, to the TME. By co−loading chemotherapeutics with immune−checkpoint inhibitors, nanobubbles facilitate chemo−immunotherapeutic synergy (71, 72). Finally, their nanoscale size permits lymphatic drainage to draining lymph nodes, where they can activate intranodal DCs (73).

3. Rational decision-making criteria for platform selection

This core distinction should inform platform selection. For targets confined to the vascular endothelium, the vascular–tissue interface, or compartments accessible via transient barrier disruption (e.g., the CNS after BBB opening), microbubbles are preferred because of their high cavitation efficiency and established safety profile (40, 74). Conversely, when direct targeting of extravascular tumor cells, tumor−associated immune cells, or lymph node populations is required, the extravasation capability of nanobubbles is essential (72, 73, 75). In an orthotopic liver tumor model, a head−to−head comparison of doxorubicin−loaded nanobubbles versus microbubbles showed that nanobubbles achieved higher intratumoral drug concentrations and superior therapeutic efficacy attributable to extravasation (72). Emerging hybrid strategies—such as using microbubbles to transiently increase vascular permeability followed by nanobubble delivery to extravascular targets—offer a promising route for sequential, precision acoustic immune−remodeling interventions (73).

##### 2.2.1.2 Phase-change acoustic nanodroplets: an *in situ* amplification strategy from nanoscale to microscale

Phase-Change Acoustic Nanodroplets can be classified as a “third-generation” gas core platform that synthesizes the advantages of both microbubbles and nanobubbles, positioning them as a highly promising new generation of ultrasound-responsive carriers. These droplets are formulated by emulsifying liquid perfluorocarbons, specifically perfluoropropane (C_3_F_8_, boiling point approximately -37°C) and perfluoropentane (C_5_F_12_, boiling point approximately 29°C), which possess boiling points below body temperature, into nanoscale droplets measuring typically less than 500 nm (76). A key to their stability in circulation is the significant Laplace pressure (ΔP = 2γ/r, where γ is surface tension and r is the droplet radius) within the nanodroplets. This elevated internal pressure, a consequence of their small radius of curvature, effectively suppresses spontaneous vaporization by raising the actual boiling point of the encapsulated PFC above physiological temperature (77). This innovative design effectively integrates the permeation capabilities of nanobubbles with the cavitation effects of microbubbles within a spatiotemporally controllable “two-phase” platform.

During the *in vivo* circulation phase, the liquid core enables nanosized droplets to demonstrate pharmacokinetic properties akin to those of conventional nanoparticles, including prolonged circulation, high stability, and accumulation within tumor tissues via the EPR effect. When the accumulation within the tumor reaches its peak, focused ultrasound energy is applied to trigger “Acoustic Droplet Vaporization” (ADV), whereby the liquid perfluorocarbon instantaneously transitions into gas, resulting in rapid volume expansion (up to 5–6 times) and the *in situ* formation of micron-sized bubbles (78). This dramatic nano-to-micro phase transition not only produces stronger mechanical disruption effects than traditional microbubbles—attributable to the volume expansion pressure associated with the phase transition—but also enables a “nuclear explosion”-like release of the drug loaded internally (79).

In the field of immune remodeling, phase-change nanodroplets are particularly suited for applications that require deep tissue penetration and robust local activation. Sonodynamic treatment (SDT) enhances tumor immunogenicity; phase-change droplets loaded with ultrasound sensitizers, following ADV, produce substantial amounts of ROS during the cavitation process, which can induce ICD and release tumor antigens and DAMPs. Additionally, mechanical immune activation occurs as the strong mechanical forces generated by ADV directly disrupt immunosuppressive microenvironments and activate mechanosensitive channels, such as Piezo1, thereby promoting immune cell infiltration. Furthermore, ultrasound imaging-guided precision immunotherapy utilizes gas bubbles formed after phase transition as ultrasound contrast agents, allowing for real-time monitoring and adjustments of therapeutic doses.

##### 2.2.1.3 Liposomal and polymeric nanocarriers: multi-stimuli responsive DDS

These carriers do not contain gas cores; rather, they serve as indirect or synergistic response platforms that primarily react to changes in the physical environment (e.g., temperature increase, pH alteration) or mechanical environment (e.g., shear stress, pressure waves) induced locally by ultrasound. Additionally, they can interact synergistically with microbubbles and nanobubbles generated either exogenously or *in situ* to facilitate controlled drug release.

1. Temperature-Sensitive Liposomes (TSLs):

Liposomes are traditional drug delivery carriers; however, by precisely designing their phospholipid components (e.g., using DPPC, which exhibits a gel-to-liquid crystalline phase transition near physiological temperature), they can be engineered to be highly sensitive to temperature (80). When HIFU induces mild hyperthermia (40-43°C) in the targeted area, the membrane structure of TSLs undergoes a dramatic phase transition, resulting in a sharp increase in permeability and the rapid release of encapsulated water-soluble drugs within minutes (81). In this system, ultrasound acts as a precise “remote heater,” while thermosensitive liposomes serve as heat-activated “nano-valves.”

In the context of immune remodeling, the most prominent application of TSLs is their synergistic effect with HIFU ablation. HIFU ablates the tumor core, inducing ICD and releasing antigens, while TSLs release chemotherapeutic agents (such as doxorubicin) at the margin of the ablation, resulting in a multimodal synergy of “physical ablation + chemical cytotoxicity + immune activation.” Additionally, TSLs can serve as carriers for immunosuppressants (such as dexamethasone) in the localized and precise treatment of autoimmune diseases, effectively confining potent immunosuppressive effects to the affected tissue while avoiding systemic immunosuppression. Presently, TSL-based ThermoDox^®^ (doxorubicin-loaded thermosensitive liposomes) is undergoing clinical trials for conditions such as liver cancer.

2. Polymeric Nanosystems

Nanoparticles or micelles composed of biodegradable polymers, such as poly(lactic-co-glycolic acid) (PLGA) and polycaprolactone (PCL), efficiently encapsulate hydrophobic drugs and respond to ultrasound through various mechanisms (82, 83): thermal-responsive degradation, where the thermal effects of ultrasound accelerate the hydrolysis and degradation of polymers, thereby facilitating drug release; cavitation-induced synergistic disruption, in which the substantial mechanical force generated by the inertial cavitation of microbubbles directly damages the structure of polymer nanoparticles, enabling forced drug release; and mechanically-triggered chemical release, a more innovative approach that involves designing mechanophore-integrated polymers. These polymers incorporate chemical bonds, such as disulfide bonds and azobenzene bonds, within their polymer backbone or crosslinking points, which can selectively break under ultrasonic mechanical force. This strategy achieves precise drug delivery triggered by “mechanical force chemistry” without requiring a temperature increase or cavitation (84).

In the context of immune remodeling, polymer nanosystems have several applications: delivery of STAT6 siRNA for reprogramming TAMs—wherein PLGA nanoparticles carrying this cargo downregulate key transcription factors of M2 macrophages following ultrasound-triggered release, thus promoting the transition from M2 to M1; combination delivery of multiple drugs—co-loading chemotherapeutic agents, ICIs, and immune adjuvants within a single nanoparticle to achieve simultaneous release through ultrasound, thereby maximizing therapeutic synergy; and immunogenic nano-vaccines—polymer nanoparticles loaded with tumor antigen peptides and adjuvants, enhanced by ultrasound for improved delivery to draining lymph nodes and uptake by DCs.

##### 2.2.1.4 Inorganic nanoparticles: a new mechanism for endogenous ultrasound response and immune regulation

In addition to the previously mentioned organic material-based carrier platforms, a new class of inorganic nanomaterials is exhibiting distinct endogenous ultrasound-responsive capabilities. These materials leverage their inherent physicochemical properties—such as the piezoelectric effect, sonocatalysis, and mechanochemical transduction—to convert acoustic energy into immune-regulatory outputs, including ROS, electric fields, and the release of structural drugs. This results in novel mechanisms of action that organic systems cannot replicate (85–87).

1. Piezoelectric Nanoparticles (PNPs)

Piezoelectric nanoparticles are at the forefront of innovative approaches in acoustic immune regulation. Materials such as barium titanate (BaTiO_3_, BTO), bismuth ferrite (BiFeO_3_, BFO), and ZnO nanowires exhibit non-centrosymmetric crystal structures. When exposed to ultrasound-induced mechanical strain, charge separation occurs within the lattice, generating a local electric field (85, 88, 89). Simulation studies conducted by Marino et al. demonstrate that piezoelectric BTO nanoparticles can produce oscillating voltages ranging from 1 to 10 mV under ultrasound stimulation (89). These electric fields can modify cell membrane potentials, regulate ion channel activity, and activate mechanotransduction pathways—effects that have been harnessed for immune modulation.

Recent studies have confirmed that piezoelectric BTO nanoparticles can significantly affect the functions of various immune cells through calcium ion influx and ROS generation induced by the piezoelectric field under ultrasound stimulation (1 MHz, 0.1–0.5 W/cm²). *In vitro* experiments demonstrate that ultrasound-driven BTO nanoparticles exhibit a pronounced anti-proliferative effect on HER2-positive breast cancer cells. This effect has been shown to result from the electrical stimulation generated by the piezoelectric effect, rather than solely from ultrasound exposure or non-piezoelectric nanoparticles (85). Importantly, this “electroacoustic immune regulation” occurs at ultrasound intensities significantly below the thermal injury threshold, offering a novel regulatory dimension distinct from traditional thermal and cavitation mechanisms.

The application of piezoelectric nanoparticles in cancer immunotherapy is particularly noteworthy. Pu et al. developed an ultrasound-triggered piezo-catalytic immunotherapy strategy that incorporates glycosylation inhibition (90). They co-loaded BiFeO_3_ nanosheets and 2-deoxyglucose (2-DG) into an injectable hydrogel. The piezo-catalytic effect generated by ultrasound stimulation not only induces ICD but also significantly enhances the phagocytosis of tumor cells by macrophages, reducing the “don’t eat me” signal (CD47) on the surface of tumor cells through inhibition of N-glycosylation. The study demonstrated significant suppression of both *in situ* tumors and distal metastases in a 4T1 breast cancer model, accompanied by DC maturation, increased polarization of M1 macrophages, and enhanced infiltration of CD8^+^ T cells (90). Additionally, a recent bioRxiv preprint (2025) reported that BTO piezoelectric nanoparticles can induce M1 polarization in bone marrow-derived macrophages (BMMs), providing preliminary evidence for the direct modulation of innate immune cells via piezoelectric stimulation (91).

2. Metal-Organic Frameworks(MOFs)

MOFs are porous crystalline materials formed by the self-assembly of metal ions or clusters with organic ligands through coordination bonds. They exhibit an exceptionally high specific surface area (up to 6000 m²/g) and adjustable pore sizes, rendering them ideal platforms for drug loading. Under ultrasound, the response mechanisms of MOFs include structural collapse to release cargo (where ultrasound vibrations disrupt coordination bonds) and enhanced release kinetics due to ultrasound cavitation (where cavitation waves accelerate the dissociation of MOFs in conjunction with microbubbles) (92–95).

In recent years, significant advancements have been made in the use of MOFs in cancer immunotherapy. MOF-based STING agonist delivery systems have shown considerable promise, with multiple studies indicating that MOFs can effectively deliver STING agonists, such as cGAMP and c-di-GMP, to tumor cells, thereby activating the cGAS-STING pathway and inducing type I interferon responses along with anti-tumor immunity (92, 96, 97). Notably, manganese-based MOFs have attracted particular attention in cancer immunotherapy due to their biocompatibility and multifunctionality. Manganese ions not only act as STING agonists to directly activate the cGAS-STING pathway but also catalyze the production of ·OH from H_2_O_2_ through the Fenton reaction, enhancing ROS-mediated ICD (92, 98). Additionally, membrane-coated biomimetic nano-MOFs have been developed to evade immune clearance and actively target tumors. Following ultrasound disruption of the membrane layer, the MOFs become exposed, facilitating the release of loaded CpG adjuvants and amplifying DC (DC) activation effects (99).

3. Mesoporous Silica Nanoparticles (MSNs)

MSNs feature tunable mesoporous structures with pore sizes ranging from 2 to 50 nm and are easily modifiable at the surface. By incorporating “acoustic-responsive nano-gates” at the pore openings, such as polymer caps that respond to ultrasound-induced temperature or mechanical forces, they enable on-demand release of cargo (100, 101). In immune applications, MSNs have been employed for targeted delivery of CpG adjuvants to DCs in lymph nodes. Specifically, MSNs loaded with CpG and modified with lymph node-homing peptides can activate the DC population in the lymph nodes following ultrasound-triggered release. Additionally, MSNs facilitate controlled release of STING agonists, which activate the STING pathway and induce a strong type I interferon response; however, systemic administration carries significant toxicity. Moreover, MSNs allow for precise local release of STING agonists at tumor sites (97, 100).

4. Semiconductor Sonosensitizers

TiO_2_, ZnO, and porphyrin-based polymer nanoparticles are semiconductor materials capable of generating ROS, particularly ¹O_2_, under ultrasound cavitation. This process is known as SDT (102–105). The underlying mechanism involves the excitation of sonosensitizer molecules in a high-energy density environment created during cavitation, leading to the formation of electron-hole pairs that subsequently react with nearby oxygen molecules to produce ROS.

In immune remodeling, SDT plays a pivotal role in inducing ICD. The pyroptosis triggered by SDT initiates gasdermin-mediated cell death, resulting in the release of IL-1β and IL-18, thereby reshaping the TME (105). When used in conjunction with ICIs, SDT facilitates multifaceted remodeling of the immune microenvironment, significantly inhibiting the growth of both primary tumors and metastases (106). For instance, the Z-type TiO_2_@CeO_2_ nanozyme/SDT/immunotherapy synergistic platform induces ICD through the SDT effect of TiO_2_, while CeO_2_ nanozymes alleviate tumor hypoxia and scavenge excess ROS via superoxide dismutase (SOD)-like and catalase (CAT)-like activities, achieving precise immune activation without damaging normal tissues (103). Additionally, surface metal modification (such as with Au or Pt) can significantly enhance the sonocatalytic efficiency of semiconductor materials like TiO_2_. The structural design of TiO_2_ nanosheets modified with Au nanocrystals at the edges exhibits excellent anti-tumor and anti-metastatic effects across various tumor models (105).

5. Advantages and Challenges of Inorganic Platforms

Inorganic nanoparticles offer several advantages, including high chemical stability, adjustable physicochemical properties (magnetic, optical, electrical), and the capacity to integrate multiple functions, such as imaging and therapy. Nevertheless, challenges persist, including the potential risks associated with heavy metal accumulation, which require improvements in biodegradability—such as using manganese or iron-based materials instead of heavy metals like barium or lead (85, 92). Additionally, these nanoparticles exhibit weaker acoustic responses under low ultrasound intensity compared to gas core systems, necessitating parameter optimization or designs that incorporate microbubbles (102). Furthermore, there is a pressing need for more extensive long-term biosafety data. Future directions should focus on developing biodegradable inorganic nanomaterials, such as hollow MOFs and iron-based piezoelectric nanoparticles, as well as organic-inorganic hybrid systems. Examples include surface modifications of liposomes with piezoelectric nanoparticles and the integration of sonosensitizers with biodegradable MOFs, which aim to harness the advantages of both material types while minimizing potential toxicity (92, 107).

#### 2.2.2 Inherent bioactivity of URPs and exogenous active payloads

Following a systematic explanation of the diversity of carrier platforms for URPs, this section further examines the mechanisms through which these platforms facilitate immune remodeling. This includes their intrinsic biological activity (agent-free effects) and the functional enhancement achieved by loading exogenous molecules.

1. Intrinsic Immunoregulatory Activity of URPs.

Before exploring the functionality of URPs as carriers, it is crucial to understand a key concept: the combination of blank URPs and ultrasound, without any exogenous drug loading, inherently possesses significant immunoregulatory activity. This bioactivity primarily arises from the intense mechanical forces induced by ultrasound-driven URPs, which trigger mechanobiological responses (120). Recent studies have confirmed that the fluid shear forces and sonoporation effects generated by oscillating microbubbles can directly activate mechanosensitive ion channels on cell membranes, such as Piezo1, thereby inducing apoptosis in pancreatic cancer cells (121). In another study, microbubble-enhanced ultrasound, mediated by a low mechanical index (MI), significantly increased muscle blood perfusion and reduced necrosis during the early stages of surgery in a mouse model of hind limb ischemia (HLI) (122).

More importantly, some studies suggest that low-pressure pulsed focused ultrasound, combined with microbubbles, may serve as a valuable tool for triggering anti-cancer immune responses. Low-pressure pulsed focused ultrasound (0.6 and 1.4 MPa) combined with microbubbles promotes sustained infiltration of non-T regulatory tumor-infiltrating lymphocytes (TILs) and CD8+ cytotoxic T lymphocytes (CTLs), significantly increasing the CD8+/Treg ratio and inhibiting tumor growth (50). A study on microbubble-mediated focused ultrasound immunoregulation in human T cells demonstrated that this method alters the concentrations of key analytes, including IL-1β, TNF-α, TNF-β, CCL21, CX3CL1, and soluble CD40L, by modulating critical signaling pathways in the immune response, such as NFκB and TNF pathways, thus enhancing the efficacy of cancer immunotherapy (123). This immune activation, triggered solely by physical forces, forms the cornerstone of the biological effects underlying “acoustic immune reprogramming”.

2. Strategic Integration of Immunoregulatory Payloads.

Building on this foundation, the loading of diverse exogenous payloads significantly enhances and refines the immune remodeling capabilities of URPs. These payloads can be systematically classified based on their specific roles in immune remodeling.

1. Immune Response Inducers: These payloads are specifically engineered to transform the immunosuppressive “cold” microenvironment into an immunostimulatory “hot” microenvironment. Typical molecules include Toll-like receptor (TLR) agonists, such as R848. Encapsulating these molecules within URPs facilitates burst release at high concentrations within tumors upon ultrasound activation, thereby effectively activating natural immune cells, including DCs and natural killer (NK) cells.
2. Immune Cell Function Reprogramming Molecules: This category represents one of the most disruptive types of effective payloads utilized in “acoustic immune remodeling”. Rather than directly inducing cell death, these molecules are designed to reprogram the functional characteristics of specific immune cells. Their applications range from altering cellular metabolism to influencing the expression of key transcription factors. For instance, M1 polarization agents are employed to reverse the phenotype of TAMs, while certain compounds disrupt the stability of regulatory T cells (Tregs). The specific strategic applications of these molecules—especially regarding their potential to proactively reshape the entire immune microenvironment—will be explored in depth and systematically in Chapter Three.
3. Adaptive Immune Enhancers: To elicit a specific and long-lasting immune response, URPs can be employed for delivering TAAs or neoantigen peptides. Ultrasound-mediated delivery not only synchronizes the timing and spatial distribution of antigens with natural immune activation signals, such as DAMPs or co-delivered adjuvants, but also enhances the internalization of antigens by antigen-presenting cells (APCs) through the acoustic perforation effect, thereby significantly improving T cell activation efficiency.
4. Immune Tolerance Inducers and Inflammation Resolution Agents: In the treatment of autoimmune diseases, such as RA and IBD, the approach is fundamentally different. At this stage, URPs are employed for the precise delivery of anti-inflammatory drugs (e.g., dexamethasone and methotrexate) or immunosuppressive cytokines to localized lesions (124–126). The key advantage lies in confining the potent immunosuppressive effects strictly to the affected tissues, thus restoring local immune homeostasis and promoting inflammation resolution without inducing systemic immunosuppression.

## 3 Core strategy of “acoustic immune remodeling”: cell function reprogramming across the disease spectrum

**Figure 3:**
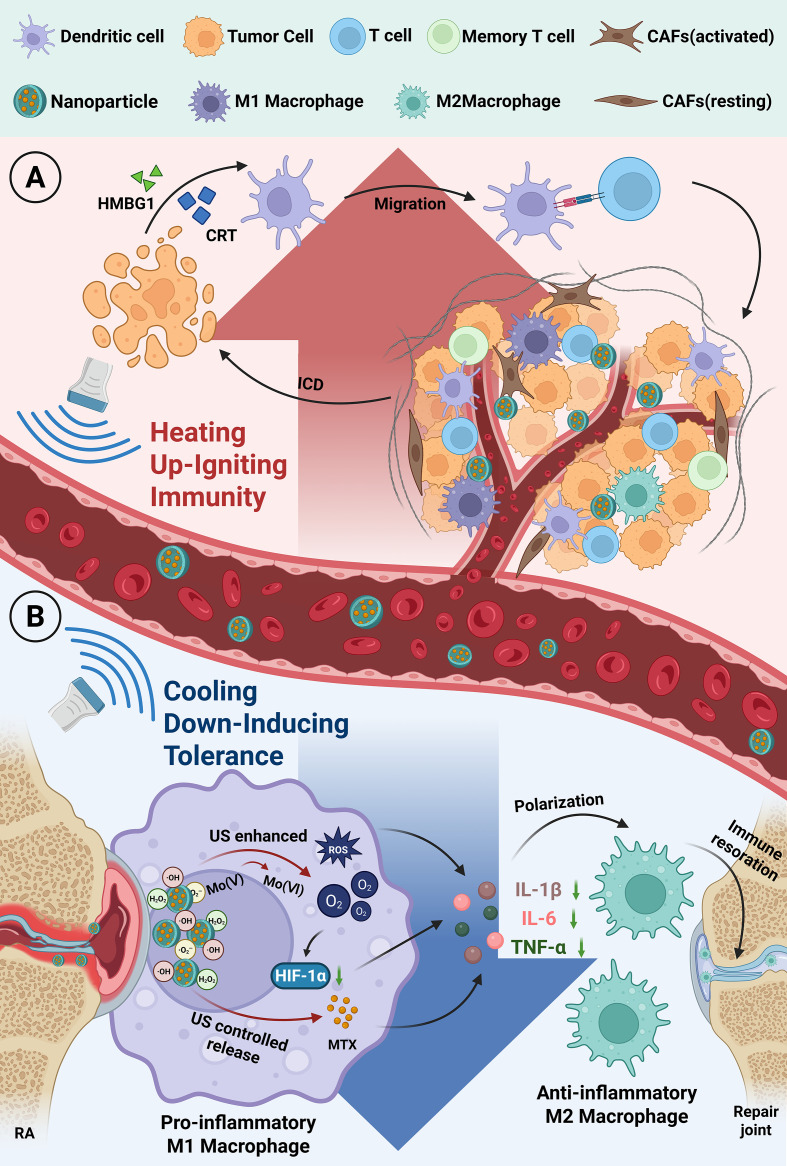
Programmability and bidirectional regulation across a spectrum of diseases (Created in https://BioRender.com): **(A)** Heating Up - Igniting Immunity: Ultrasound-triggered “*in situ* nanovaccines” amplify the immune activation cascade, converting “cold” tumors into “hot” immune-responsive tumors, thereby stimulating adaptive anti-tumor immunity (118). **(B)** Cooling Down - Inducing Tolerance: Precise delivery of nanocarriers to targeted lesions remodels the immune microenvironment, “inducing” immune tolerance and effectively driving the polarization of pro-inflammatory M1 macrophages toward the anti-inflammatory M2 phenotype, thereby alleviating the progression of autoimmune diseases (52).

Cell function programming is the central strategy of “Acoustic Immune Remodeling”(**Figure 3**). This approach focuses on key immune and stromal cells to comprehensively examine how it enables the specific programming or reprogramming of cell functions through precise spatiotemporal regulation(**Table 3**). This process spans various pathophysiological conditions, including tumors, autoimmune diseases, cardiovascular disorders, and inflammatory diseases, ultimately directing the pathological microenvironment toward predefined therapeutic outcomes.

**Table 3:** Summary of core therapeutic strategies and representative studies in acoustic immune remodeling.

Therapeutic strategy	Acoustic platform and parameters	Payload	Effector immune cells	Major immunological findings	Therapeutic effect.	Reference
Immune activation and potentiation	LMC + ultrasound trigger.	Doxorubicin	T cells, DCs.	Inducing enhanced ICD, driving DC maturation, and activating CTLs.	Achieving robust activation of anti-tumor immunity.	(110)
	UMC + pH-responsive liposomes.	TLR7/8 agonist imiquimod (R837).	Macrophages, DCs, T cells.	Promoting the reprogramming of M2-type TAMs to M1-type, synergistically facilitating DC maturation and cytokine secretion, and downregulating immune checkpoint molecules.	Significantly enhancing anti-tumor immunity and activating multicellular networks.	(111)
	Focused ultrasound-responsive nanocomposites (with sonoluminescent properties).	Photosensitizer	DCs	Focused ultrasound precisely targets the draining lymph nodes, where nanomaterials *in situ* activate photosensitizers through luminescence, thereby directly activating the “immune command center”.	The first successful demonstration of remote, non-invasive direct activation of lymph nodes.	(115)
	Synthetic biology + ultrasound-responsive polymer sonosensitizers.	Engineered bacteria expressing OX40L.	T cell	The sonodynamic effect induces ICD, provides antigens, synergistically exposes the OX40L co-stimulatory signal, and directly activates T cells.	The formation of a cascade-amplified, durable immune memory and anti-metastatic effect.	(114)
Induction of immunosuppression and tolerance.	UTMD	FK506 immunosuppressant	T cell	Significantly inhibiting T cell infiltration and the secretion of inflammatory cytokines to achieve localized, targeted immunosuppression.	The average graft survival time was prolonged from 12.83 days to 16.00 days, thereby avoiding systemic immunosuppressive side effects.	(58)
	Cerasomal nanoregulators (DMC@P-Cs) + ultrasound trigger.	DMC	Tregs, CTLs.	Specific inhibition of Treg function enhances CTL activity, facilitating precise regulation of the CTL/Treg ratio.	Paving new pathways for organ transplantation and the treatment of autoimmune diseases.	(117)
	UTMD serves as a non-viral gene delivery vector.	CRISPR/Cas9 system.	Tregs	Targeted knockout of the key Treg transcription factor Foxp3 enables precise reprogramming or depletion of immunosuppressive Treg cells.	The inhibitory effect of Tregs on CTLs was fundamentally alleviated.	(113, 116)
Matrix Remodeling	Nanoenzymes Loaded with Sound Sensitizers.	Sound sensitizers	FLS	Precise sonodynamic elimination of overproliferated FLS.	Inhibiting the pathological fibrosis process from its source to break the vicious cycle of inflammation and tissue damage.	(53)
	UTMD	TGF-β1/TIMP1 dual gene vector	Macrophages	Inhibiting macrophage activation, remodeling phenotypes, reducing inflammatory responses, and promoting plaque stability through matrix reconstruction.	Enhancing the stability of atherosclerotic plaques and improving the extracellular matrix environment of the vascular wall.	(57)
	Sound sensitizer and functionalized graphene nanoribbon composite.	Sound sensitizers	Stromal cells	Simultaneously achieving sonodynamic ablation, physically obstructing the adhesion of cancer cell spheroids and remodeling the tumor matrix structure.	Inhibiting the metastasis of ovarian cancer and altering the matrix characteristics of the TME.	(149)
Barrier Breakthrough	LIPU + Microbubbles.	—	Endothelial cells, immune cells.	Temporarily and reversibly opening the tight junctions between endothelial cells through sonoporation.	Facilitate the infiltration of immune cells and therapeutic drugs, thereby increasing the median survival time from 8.9 months to 17.6 months.	(39–42)
	HIFU + FUS	—	Immunologic effector cell	Decrease IFP by disrupting the dense network of collagen fibers through acoustic cavitation and mechanical forces.	Enhance the flow of tumor stromal fluid to facilitate drug penetration and promote the infiltration of immune cells.	(143)
	Sequentially targeted sonodynamic nanovaccine.	CpG adjuvant	DCs	Traverse the endothelial cells of the BBB in sequence to precisely deliver the adjuvant to the DC activation site.	Induce a sufficiently strong T-cell response to inhibit distant metastasis and establish systemic immune memory.	(180)
	Engineered neutrophil carrier system + Ultrasound.	Therapeutic payload	Neutrophils	Leverage the intrinsic capacity of neutrophils to cross the BBB for targeted delivery to the central nervous system, guided by ultrasound.	Overcome the delivery challenges associated with the treatment of central nervous system tumors.	(167)
Multicellular Network Coordination and Regulation	Multifunctional nanocomposites + Focused ultrasound activation.	Docetaxel + PD-L1 antibody.	MDSCs, T cell	Simultaneously block key immunosuppressive signaling pathways and cell populations, effectively reducing MDSCs and inhibiting the PD-L1 pathway.	Enhance T-cell function synergistically to achieve a therapeutic gain greater than the sum of its parts.	(108, 109)
	Vortex-based acoustic vortex-controlled intelligent microrobots.	CpG adjuvant	DC, T cells, macrophages, NK cells.	Induce controlled, active collective movement under the influence of an external acoustic field to uniformly distribute the immunoadjuvant throughout the tumor mass.	Address the challenges associated with uneven drug distribution in large solid tumors and the low efficiency of immune activation to promote systemic antitumor immunity.	(181)
	LDH Nanoplates + Sonodynamic Therapy.	Sonosensitizer ICG + PAD4 Inhibitor GSK484.	Neutrophils	Inhibit the formation of NETs and disrupt pro-metastatic structures.	Achieve dual efficacy in both “treating the primary tumor” and “preventing metastasis”.	(164)

This table summarizes representative studies in acoustic immune remodeling categorized by therapeutic strategies, covering five major strategies: immune activation, immunosuppression, matrix remodeling, barrier penetration, and multi-cellular network regulation. Numbers in square brackets refer to reference citations. Major abbreviations: LMC, liposome-microbubble complexes; ICD, immunogenic cell death; DC, dendritic cell; CTLs, cytotoxic T lymphocytes; UMC, ultrasound cavitation; TAMs, tumor-associated macrophages; UTMD, ultrasound-targeted microbubble destruction; DMC, demethylated cantharidin; Tregs, regulatory T cells; FLS, fibroblast-Like synoviocytes; LIPU, low-intensity pulsed ultrasound; HIFU, high-intensity focused ultrasound; FUS, focused ultrasound; IFP, interstitial fluid pressure; MDSCs, myeloid-derived suppressor cells; PD-L1, programmed death-ligand 1; LDH, layered double hydroxide; NETs, neutrophil extracellular traps.

### 3.1 Macrophage and myeloid lineage reprogramming: masters of immune microenvironment plasticity

Within the immune microenvironment, macrophages and myeloid-derived suppressor cells (MDSCs) are not merely bystanders but pivotal orchestrators of immune responses. Their preeminent status as targets for acoustic immune reprogramming stems from three intrinsic biological properties that align perfectly with the capabilities of ultrasound-responsive nanoplatforms. First, their exceptional functional plasticity, exemplified by the classic M1 (pro-inflammatory) to M2 (anti-inflammatory) polarization spectrum, allows for profound phenotype reversal rather than mere inhibition or activation. Second, their strategic positioning as abundant tissue-resident and infiltrating cells ensures they are first-line responders and key regulators in pathological sites, from tumors to atherosclerotic plaques. Third, their role as central signaling hubs—secreting a wide array of cytokines, chemokines, and growth factors—means that reprogramming a single myeloid cell can initiate a cascade that reshapes the entire local immune landscape. The following sections will dissect how acoustic immune remodeling leverages these unique attributes to forcibly recalibrate the phenotypes and functional states of these cells across the disease spectrum, transforming them from engines of pathology into pillars of therapy.

#### 3.1.1 Tumor microenvironment: functional reprogramming of myeloid cells from a “pro-cancer” to an “anti-cancer” phenotype

In the TME, TAMs and MDSCs are critical components in the formation of immune-suppressive barriers. Acoustic immune remodeling seeks to reverse their pro-cancer functions. The core strategies include the following:

First, Reversing the Immune Suppressive Phenotype (M2→M1). Employing URPs to deliver TLR agonists, such as the TLR7/8 agonist Imiquimod (R837) and the TLR9 agonist CpG oligonucleotides, along with ultrasound-enhanced intracellular delivery, can upregulate the transcription of pro-inflammatory cytokines. This process reprograms immunosuppressive M2-type TAMs into M1-type macrophages with phagocytic and antigen-presenting capabilities. One study utilized ultrasound cavitation (UMC) to facilitate the delivery of pH-responsive liposomes (PEOz-Lip@R837), which carry the TLR agonist R837 to tumor sites. UMC-induced TAAs, in the presence of immune adjuvants, synergistically promoted DC maturation and cytokine secretion while downregulating immune checkpoint molecules such as CD274, Foxp3, and CTLA4, thereby enhancing T cell activation and proliferation. This strategy significantly boosts anti-tumor immune responses, effectively inhibits both primary and metastatic tumor growth, and establishes immune memory to prevent recurrence (62). To address the bottleneck of low gene transfer efficiency in tumor gene immunotherapy, researchers combined the mini-plasmid pFAR4 encoding interleukin-12 (IL-12) with lipid-mediated TLR2 agonists and HIFU. After optimizing the conditions, they evaluated efficacy in the CT26 colon cancer mouse model. The results demonstrated that single components had no significant inhibitory effect on tumor growth, while the combination of all three substantially inhibited tumor growth, prolonged survival, and elicited non-local anti-tumor effects. This combined strategy effectively activated anti-tumor immune responses, underscoring its clinical potential (127). Shu Hong and colleagues successfully delivered STAT6 siRNA using poly PLGA-polyethylenimine (PEI) nanobubbles in conjunction with ultrasound-mediated nanobubble disruption, enhancing the transfection of PLGA-PEI NBs-STAT6 siRNA in M2-like macrophages, reprogramming them into M1-like macrophages, and inhibiting the proliferation, migration, invasion, and epithelial-mesenchymal transition (EMT) of non-small cell lung cancer cells (128).

Second, Reconstructing the Microenvironment Supporting TAM Survival: Tumor hypoxia drives TAM polarization toward the M2 phenotype primarily through activation of HIF-1α. Specially designed oxygen-carrying URPs (O2-MBs) enable “acoustic oxygenation” to directly alleviate hypoxia. This in turn inhibits HIF-1α activity, blocking M2 polarization at the transcriptional level (129). One study developed an ultrasound-mediated oxygen microbubble (OPMB)-enhanced strategy with ultrafine polyethylene glycol-modified protoporphyrin IX micelles (PPM) for hypoxic TMEs. Ultrasound disruption of OPMBs enabled the targeted release of oxygen and photosensitizers to the tumor, significantly improving tumor oxygenation and singlet oxygen production, inhibiting HIF-1α and related pathways, and blocking angiogenesis and EMT. This approach effectively suppressed the growth and metastasis of breast and pancreatic cancers, highlighting the powerful potential of ultrasound-mediated oxygen-enhanced photodynamic therapy (PDT) (130). In similar research, a perfluorocarbon nano-microbubble (D-vpcs-O2) with a core-shell structure of polyorganosiloxane and pH-sensitive tumor-targeting peptides was developed, achieving co-delivery of oxygen and doxorubicin. HIFU induced the precise release of oxygen and drugs from the microbubbles, downregulated TGF-β1 expression, alleviated hypoxia and multidrug resistance, suppressed EMT and tumor metastasis, and enhanced chemotherapy efficacy. To address the metastatic and treatment-resistant challenges of triple-negative breast cancer (TNBC), a team developed red blood cell membrane-camouflaged nanoparticles (SB-IR-PLGA@RM), which encapsulate the sonosensitizer IR780 and TGF-β inhibitor SB431542 to drive TAMs and neutrophils towards an anti-tumor phenotype, reshaping the microenvironment, inhibiting cancer-associated fibroblast (CAF) activation, and blocking EMT. This platform, combined with anti-PD-L1 therapy, significantly enhanced anti-tumor immunity, offering a novel strategy to overcome the treatment bottleneck of TNBC (22). All these studies demonstrate the substantial clinical potential of ultrasound-mediated oxygen delivery and drug co-release strategies.

Third, Targeting and Eliminating the Suppressive Function of MDSCs: MDSCs suppress T cell function through various mechanisms, including the production of ROS, arginase 1 (Arg1), and the induction of Tregs. Utilizing URPs to deliver therapeutic agents that promote the differentiation of MDSCs into mature, non-suppressive macrophages or DCs, or that directly inhibit MDSC function, allows for the precise disruption of this key immune-suppressive cell population in the TME. One study employed ultrasound-activated semiconductor polymer nanomaterials (SPNTi) integrated with sonodynamic agents, hypoxia-responsive tirapazamine (TPZ), and the MDSC-targeting drug ibrutinib to synergistically enhance ICD and remodel the tumor immune microenvironment by targeting immunosuppressive MDSCs. The ultrasound activation of SPNTi generated ROS, triggering the degradation of the polymer shell and the subsequent release of the drug. TPZ amplified ICD, while ibrutinib reduced MDSC activity, significantly inhibiting both primary and distal tumor growth and blocking metastasis, thus demonstrating high efficacy and precision in anti-tumor immunotherapy (131). In similar research, drug-loaded microbubbles (RD@MBs) combined with ultrasound-targeted microbubble disruption (UTMD) technology were utilized to achieve tumor-targeted release of docetaxel and imiquimod, promoting the release of TAAs and the activation of T cells. This strategy enhanced the accumulation of αPD-L1 at the tumor site while reducing MDSCs and modulating TAM polarization, thereby remodeling the immunosuppressive microenvironment. The approach significantly improved the effectiveness of PD-L1 blockade therapy, suppressing both primary and distant tumor growth and metastasis (108).

In the tumor immune microenvironment, the heightened plasticity of macrophages and MDSCs serves as both a fundamental mechanism of tumor immune suppression and a critical avenue for therapeutic intervention. Acoustic-responsive nanoplatforms enable active reprogramming of the phenotype and functional state of myeloid cells through precise spatiotemporal control of delivery. These platforms can reverse pro-tumor phenotypes, reshape the microenvironment essential for their survival, and effectively diminish the immune suppression barrier posed by MDSCs. This paradigm facilitates the concurrent disruption of multiple mechanisms underlying tumor immune evasion, thereby highlighting the unique advantages of enhancing anti-tumor immunity.

#### 3.1.2 Autoimmune diseases: the functional transition of polarized pro-inflammatory macrophages to an “anti-inflammatory and tissue repair” phenotype

Inducing an Anti-inflammatory and Tissue Repair Phenotype (M1→M2): In autoimmune diseases or acute inflammation, the pathological process is primarily driven by M1 macrophages or overactivated neutrophils. In a RA model, the precise delivery of corticosteroids (e.g., dexamethasone) or siRNA targeting key molecules in the NF-κB signaling pathway to inflamed synovial joints via URPs effectively induces the transition from M1 to the M2 macrophage phenotype (51). One study developed a supramolecular nanoplatform based on polyoxometalates (PCSN@MTX) to target and modulate macrophages in RA. The key to this strategy is the use of ultrasound as an external trigger to achieve dual synergistic effects: on one hand, ultrasound precisely disrupts the bond between the drug (methotrexate) and the carrier, enabling on-demand release at inflammation sites; on the other hand, ultrasound significantly enhances the nanoplatform’s catalytic ability to scavenge ROS. These combined effects synergistically reshape the joint microenvironment, effectively driving the polarization of pro-inflammatory M1 macrophages to the anti-inflammatory M2 phenotype, thereby significantly alleviating disease progression (52).

#### 3.1.3 Atherosclerosis: multifactorial remodeling of intraplaque macrophage function to enhance lesion stability

UTMD technology has emerged as a key platform for the precise delivery and controlled release of macromolecular drugs to lesion sites, particularly in the targeted regulation of macrophage function. Researchers have utilized this technology to deliver IL-8 monoclonal antibodies, effectively blocking chemotactic signals and inhibiting macrophage recruitment and infiltration into plaques (55). Additionally, they have targeted the delivery of siRNA to silence the expression of key inflammatory signaling molecules, such as GSK-3β, thereby suppressing the pro-inflammatory activation state of macrophages from within and alleviating plaque inflammation (56). Furthermore, researchers have employed UTMD as a gene delivery system to efficiently transport viral vectors encoding anti-inflammatory and tissue repair factors, such as TGF-β1 and TIMP-1, to macrophages within plaques. This strategy not only drives macrophage polarization toward the M2 phenotype, which promotes tissue repair, but also directly inhibits the activity of matrix-degrading enzymes they secrete, thereby achieving the dual therapeutic goals of “inhibiting inflammation” and “promoting stability” (57). Collectively, these studies confirm that the UTMD-mediated acoustic response strategy can precisely remodel macrophage function in atherosclerotic plaques through multiple pathways, including blocking recruitment, inhibiting activation, and reshaping phenotypes, providing critical support for the development of novel acoustic immunotherapies.

### 3.2 Dual-modulation of T-cell function

T cells serve as the primary effectors of adaptive immunity, with the “activation” and “inhibition” of their functions representing the primary objectives of most immunotherapies. The acoustic immune remodeling paradigm offers programmable strategies for both activation and inhibition.

#### 3.2.1 Tumor immunity: enhancing the comprehensive life cycle of T cell anti-tumor responses

The acoustic-responsive nanoplatform is a core technological approach for activating and enhancing T cell-mediated anti-tumor immune responses, facilitating “acoustic immune remodeling”. A primary step in this strategy is to initiate the immune response. Specifically, ICD is precisely induced in the TME to release endogenous danger signals. For instance, researchers loaded the chemotherapeutic agent doxorubicin into lipid-microbubble complexes (LMC), allowing for precise drug release and deep penetration upon ultrasound triggering, which induces a more robust ICD (110). Similarly, nanoplatforms loaded with sensitizers, such as hematoporphyrin, efficiently initiate ICD under photodynamic effects, promoting DC maturation and ultimately facilitating the effective activation and recruitment of CTLs (132, 133). One study effectively combined engineered bacteria expressing the T cell co-stimulatory ligand OX40L with ultrasound-responsive polymeric sensitizers through a synthetic biology-constructed *in vivo* biological hybrid platform. Upon ultrasound triggering, the photodynamic effect not only induces ICD, providing antigens for CTL activation, but also crucially exposes OX40L on the surface of the engineered bacteria. This co-stimulatory signal directly acts on T cells, significantly amplifying and sustaining their anti-tumor cytotoxic activity, ultimately generating a cascading amplification of lasting immune memory and anti-metastatic effects, thereby offering a multifaceted activation strategy for acoustic immunotherapy against cancer (114). Additionally, researchers innovatively employed low-frequency ultrasound and microbubbles as “gene syringes” to efficiently deliver DNA vaccines encoding tumor antigens into the body, thereby directly stimulating a strong, specific T cell response from the outset (112).

Building on this foundation, the key advantage of this technology lies in its capacity to further reshape and enhance T cell functions, enabling them to overcome the immunosuppressive TME. Studies have confirmed that UTMD serves as a non-viral gene delivery system to introduce the CRISPR/Cas9 system into tumors, facilitating the precise reprogramming or depletion of immunosuppressive Tregs by targeting the essential transcription factor Foxp3, thereby fundamentally eliminating their inhibition of CTLs (113, 116). Additionally, by creating red blood cell membrane-mimicking nanoparticles loaded with TGF-β inhibitors (such as Galunisertib) (22) or delivering targeted nanobubbles containing STAT3 inhibitors (134), this approach effectively modifies the TME via ultrasound mediation, thereby blocking critical immunosuppressive signaling pathways and promoting T cell infiltration and cytotoxicity. Furthermore, studies have shown that UTMD can trigger the controlled release of nitric oxide (NO) within tumors, sensitizing the TME and enhancing T cell killing efficiency (135). Notably, this acoustic strategy can bypass the antigen presentation phase, allowing for direct functional modulation of T cells. Research indicates that the localized mechanical effects produced by low-intensity focused ultrasound and microbubbles can interact directly with T cell membranes, activating mechanosensitive ion channels to promote Ca²^+^ influx, significantly enhancing T cell proliferation and activation, as well as upregulating the secretion of key effector molecules such as interferon-γ (IFN-γ) and granzyme B (21). In summary, the ultrasound-responsive nanoplatform utilizes a synergistic strategy that encompasses the initiation of immune responses, the removal of immunosuppression, and direct functional enhancement, achieving refined spatiotemporal control over the T cell anti-tumor life cycle—from activation and infiltration to killing—thus providing a novel paradigm for the development of effective tumor immunotherapies.

#### 3.2.2 Autoimmunity and transplantation: precision strategies for inducing local immune tolerance through suppression

In scenarios requiring immune response suppression, acoustic immune remodeling employs precise suppression strategies. In the prevention and treatment of organ transplant rejection, UTMD technology has emerged as an effective method for delivering immunosuppressants and regulatory genes. Liu et al. demonstrated that FK506-loaded microbubbles combined with UTMD technology can increase the concentration of the immunosuppressant within the transplanted heart by 1.64-fold, significantly inhibiting T cell infiltration and the secretion of inflammatory cytokines, thereby extending the average graft survival time from 12.83 days to 16.00 days (58). Importantly, this technique facilitates localized targeted delivery, mitigating the adverse effects associated with systemic immunosuppression. Innovations in gene therapy further broaden the applications of ultrasound-mediated immune modulation. Wang et al. successfully delivered galectin-7 siRNA to the transplanted heart via UTMD, achieving a 50% downregulation of this pivotal immunoregulatory molecule. Given that galectin-7 promotes the polarization of Th1/Th2 cells towards Th1, its inhibition can effectively modulate acute T cell-mediated rejection, significantly reducing interleukin-2 levels and successfully preventing acute cardiac transplant rejection (59). This strategy identifies new molecular targets for the precise regulation of transplant rejection by adjusting the polarization balance of T cell subpopulations.

In the context of signaling pathway modulation, the ultrasound-mediated sirolimus delivery system exerts immunosuppressive effects by inhibiting the TGF-β1/Smad pathway. This system not only suppresses T cell proliferation and activation but also promotes autophagy and reduces inflammation (60). The synergistic effects of these mechanisms provide a comprehensive solution for treating complex immune diseases. Notably, ultrasound-mediated nanoplatforms offer distinct advantages in regulating the balance of T cell subpopulations. The cerasomal nano-regulator (DMC@P-Cs) developed by Zhang et al. selectively suppresses the function of Tregs while enhancing the activity of CTLs through ultrasound-controlled release of demethylcolchicine (DMC), thereby achieving precise modulation of the CTLs/Tregs ratio (117). This system utilizes ROS generated by ultrasound not only to directly eliminate abnormal cells but also to oxidize unsaturated phospholipids, thereby altering the permeability of the lipid bilayer and enabling spatiotemporal control of drug release. Collectively, these studies demonstrate that ultrasound-mediated nanoplatforms can precisely regulate T cell immune responses, offering new avenues for treating non-tumor diseases such as organ transplantation and autoimmune disorders.

### 3.3 Remodeling and disrupting stromal cells and physical barriers

Fibroblasts and other stromal cells can create dense physical barriers while actively participating in immune suppression under pathological conditions. Acoustic immune remodeling addresses this challenge by utilizing a dual strategy that combines physical disruption with biological remodeling.

#### 3.3.1 Disruption of the stromal barrier in tumors and remodeling of cell phenotypes

The TME of solid tumors undergoes substantial remodeling under pathological conditions, with one of its most prominent features being the formation of a dense extracellular matrix (ECM) predominantly composed of CAFs. This ECM serves as a primary physical barrier encountered by current cancer therapies, particularly in nanoparticle drug delivery and immunotherapy (136). The excessive accumulation of stroma significantly alters the physicochemical properties of tumor tissues: it not only increases tumor stroma stiffness, compressing blood and lymphatic vessels, but also leads to a sharp rise in interstitial fluid pressure (IFP) within the tumor (137). These physical changes hinder the penetration and uniform distribution of therapeutic antibodies, chemotherapeutic agents, and nanocarriers deep into the tumor (138), while also limiting the infiltration and migration of immune effector cells, such as CTLs, due to a “physical entrapment” effect. This contributes to resistance to immunotherapy and the development of an “immune exclusion” or “immune evasion” TME (139). Consequently, the physical barrier formed by CAFs is a critical obstacle that must be overcome to enable effective anti-tumor therapy.

Acoustic immunity remodeling represents a promising strategy for overcoming the physical barrier established by CAFs. Initially, this strategy emphasizes leveraging the mechanical effects of ultrasound to directly disrupt the tumor stroma. Numerous studies have demonstrated that HIFU, focused ultrasound (FUS), and UTMD effectively dismantle dense collagen fiber networks through cavitation and mechanical forces. This process enhances fluid flow within the tumor stroma and reduces IFP, thereby creating physical channels for improved drug penetration (140–142). However, the essence of this strategy extends beyond mere physical disruption; it incorporates a multi-modal synergistic approach that integrates bioactive molecules. A particularly illustrative model is the “enzymatic pioneer-ultrasound strike” framework: researchers load collagenase onto the surface of ultrasound-sensitive nanoparticles. Upon delivery to the tumor, collagenase initially acts as a “pathfinder”, specifically degrading type I and type III collagen to “soften” the stroma. The subsequent application of ultrasound activates the ultrasound-sensitive agents at greater depths, leading to the generation of cytotoxic ROS and significantly enhancing the depth of therapeutic effect (143).

Beyond enzymatic degradation, direct reprogramming of the biological phenotype of stromal cells fundamentally disrupts barriers. A particularly innovative study on pancreatic cancer designed a pH-sensitive hollow mesoporous nanos carrier for the delivery of the antifibrotic drug pirfenidone. This carrier preferentially releases the drug in the acidic environment of the TME and subsequently employs UTMD technology to enhance its local concentration, successfully reversing the activated, matrix-producing pancreatic stellate cells to a quiescent phenotype (144). Following a similar approach, other studies have successfully reprogrammed the metabolism of activated stromal cells or CAFs by targeting ultrasound-responsive nanoplatfoms to deliver all-trans retinoic acid (ATRA) or the glutamine metabolism inhibitor V9302 (61, 145). The potential for this phenotypic remodeling even extends beyond stromal cells. One study confirmed that UTMD-mediated EZH2 gene silencing effectively inhibits the EMT process in liver cancer stem cells, demonstrating its significant potential in regulating tumor invasiveness (146).

The current forefront of this field emphasizes multi-modal responsive systems with enhanced spatiotemporal controllability and greater integration of functions. Researchers have developed a “cascade response” nanosystem triggered by near-infrared light (NIR) and ultrasound, which achieves higher-dimensional spatiotemporal control over TME remodeling (147). Additionally, utilizing SDT for TME reshaping has gained mainstream acceptance. For instance, by activating semiconductor polymer nanosculptors (SPN) or specially designed ultrasound-sensitive agents through ultrasound, ROS can be generated to directly degrade the ECM while also inducing ICD, such as pyroptosis, thereby synergistically activating anti-tumor immunity (132, 148). More advanced designs integrate ultrasound-sensitive agents with functionalized graphene nanosheets, achieving sonodynamic ablation and physically blocking the adhesion of ovarian cancer cell spheroids, thereby inhibiting metastasis (149). To combine diagnosis with treatment, peptide-functionalized phase-change nanoparticles have been developed that facilitate tumor imaging under low-intensity FUS, aiding navigation for subsequent therapies (150). Ultimately, these strategies form a multi-functional treatment platform; for example, ultrasound-triggered multifunctional nanocomposites enable simultaneous targeted delivery of chemotherapy and gene therapy, illustrating substantial potential for synergistic treatment (109). In summary, ultrasound-mediated nanosystems offer a robust theoretical foundation and insightful technological paradigms for dismantling the physical and biological barriers of tumors, effectively achieving acoustic immunity remodeling through the integration of various mechanisms, including physical disruption, enzymatic degradation, cellular reprogramming, and precise multi-modal control.

#### 3.3.2 Non-tumor diseases: from fibrosis inhibition to functional regeneration promotion

The applications of “acoustic remodeling” extend well beyond oncology. Its core principle—precise modulation of stromal cells and the extracellular matrix within lesion sites via ultrasound—shows considerable therapeutic potential in a wide range of non-tumor diseases characterized by chronic inflammation, tissue fibrosis, or degeneration. In such contexts, ultrasound-responsive nanoplatforms function not merely as drug delivery “carriers” but also as active “regulators” of the microenvironment.

A primary application of this strategy is the inhibition or reversal of pathological fibrosis. RA exemplifies this application, where the abnormal proliferation of fibroblast-like synoviocytes (FLS) is a key pathogenic factor. A carefully designed study developed nanoparticles loaded with ultrasound-sensitive agents that, when activated by ultrasound, can precisely eliminate the excessively proliferating FLS within the joint cavity through sonodynamic action, thereby disrupting the vicious cycle of inflammation and tissue destruction at its origin (53). Similarly, in keloid treatment, the emphasis is on inhibiting excessive fibroblast proliferation and collagen synthesis. Research has demonstrated that utilizing ultrasound in conjunction with lipid nanobubble technology allows for the efficient delivery of anti-fibrotic drugs, such as 5-fluorouracil, to the deeper layers of the dermis, significantly suppressing the activity of scar fibroblasts and highlighting its value in treating skin fibrotic diseases (151). In more complex cases of visceral fibrosis, such as diabetic cardiomyopathy (DCM) and metabolic dysfunction-associated fatty liver disease (MASH), researchers have utilized ultrasound-mediated nanoplatforms for the targeted delivery of the anti-fibrotic protein FGF21 or for precise modulation of hepatic stellate cells (HSCs), effectively alleviating the fibrosis process in the affected organs (152, 153).

In contrast to “inhibition”, the emphasis is on promoting functional tissue regeneration and wound healing. In the treatment of knee osteoarthritis (OA), research suggests that the combination of Prussian blue nanoparticles with low-intensity pulsed ultrasound (LIPUS) can enhance the synthesis of key cartilage matrix components by activating the PI3K-Akt-mTOR signaling pathway in chondrocytes, thereby facilitating the transition from “inhibition of degeneration” to “promotion of regeneration” (154). In the realm of wound repair, acoustic remodeling exhibits even greater multifunctionality. An innovative study employed sonodynamic principles using ultrasound-activated heterojunction nanocoatings, which not only generate low concentrations of ROS to eliminate bacteria but also utilize ROS as signaling molecules to promote the proliferation and migration of fibroblasts, thereby achieving the dual objectives of “antibacterial” activity and “healing” promotion (155). Furthermore, the mechanical effects of ultrasound have been harnessed to directly optimize the physical properties of regenerative materials. For example, ultrasound treatment can produce chitosan nanofiber mats with increased porosity and enhanced liquid absorption capacity, rendering them superior hemostatic materials (156).

Of particular interest is the ability of acoustic remodeling strategies to overcome matrix barriers constructed by pathogenic microorganisms. Microbial biofilms, which serve as inherently protective extracellular matrices, are key contributors to drug-resistant infections. To address this, researchers designed nanoparticles loaded with amphotericin B, targeting fungi, and harnessed the cavitation effect of low-frequency ultrasound to disrupt the dense structure of Beauveria bassiana biofilms, significantly enhancing drug penetration and efficacy, thereby opening new avenues for the treatment of drug-resistant biofilm infections (157). In summary, whether through sonodynamic ablation of pathological cells, precise drug delivery to inhibit fibrosis, promotion of tissue regeneration, physical modification of biomaterials, or disruption of microbial protective barriers, ultrasound-responsive nanoplatforms offer a spatiotemporally controllable and functionally versatile approach for treating non-tumor diseases.

### 3.4 Igniting adaptive immunity: acoustic manipulation focused on dendritic cells

As a pivotal link between innate and adaptive immunity, the activation and maturation of DCs is crucial for initiating effective anti-tumor immune responses. The acoustic immune remodeling strategy reveals its significant potential as an “*in situ* vaccine”, precisely initiating and amplifying DC-centered immune cascades in a multi-dimensional, spatiotemporally controlled manner.

The cornerstone of this strategy is the utilization of acoustic effects to achieve “*in situ* destruction” of tumors and “immunogenic release”. Simple cellular necrosis is inadequate for eliciting a robust immune response. Consequently, research has shifted its focus toward directing cell death pathways to induce ICD and activate more robust forms of programmed necrosis, such as necroptosis, through sophisticated nanodesign. Both the mechanical damage caused by HIFU and the chemical destruction resulting from SDT can lead to significant tumor cell death, thereby releasing abundant TAAs (49, 158). A particularly notable approach involves loading the necroptosis inducer shikonin into polymeric nanobubbles. When ultrasound triggers the rupture of these bubbles at the tumor site, shikonin is precisely released, effectively converting an originally non-immunogenic cell death process into a programmed necrosis that releases substantial amounts of DAMPs, thus providing a higher-quality danger signal for DCs (159).

Building on the induction of endogenous signals, a more proactive strategy involves designing an “*in situ* nano-vaccine” that integrates “ultrasound-sensitive agents-adjuvants” to actively amplify the activation signals of DCs. Among these, MOFs and their derivatives have garnered significant attention due to their structural plasticity and functional diversity. In a study focused on pancreatic cancer, researchers utilized a zeolitic imidazolate framework (ZIF-8) as a template, co-loading it with the immunoadjuvant R837, and subsequently carbonizing it at high temperatures to produce hollow mesoporous carbon nanospheres. This unique structure efficiently loads the adjuvant while its carbon shell functions as an excellent ultrasound-sensitive agent. Upon ultrasound activation, it generates ROS to induce ICD while concurrently releasing R837, achieving optimal spatiotemporal synchronization between “antigen generation” and “adjuvant activation”, effectively transforming the “cold” pancreatic tumor into a “hot” immune-responsive tumor (118). In a separate study, a “biomimetic” design was implemented for the MOF platform: researchers encapsulated MOFs loaded with CpG adjuvants within cancer cell membranes. This biomimetic design facilitates effective evasion of immune system clearance and precise targeting of homologous tumors. Ultrasound not only triggers adjuvant release but also disrupts the membrane camouflage, exposing the internal MOF and further amplifying DC activation (63).

Other studies have concentrated on actively remodeling the TME to “remove obstacles” to DC functionality. Tumor hypoxia represents a significant barrier to DC maturation and function. To address this challenge, researchers have developed a range of “oxygen-supplying” nanosensitizers. For example, a hypoxia-responsive molybdenum oxide nanosensitizer reduces internal Mo(VI) to Mo(IV) in the acidic and hypoxic microenvironment of tumors. This reduction process not only consumes protons that inhibit the immune response but also produces oxygen directly. This “waste-to-resource” design, triggered by ultrasound, achieves a threefold synergy of “alleviating hypoxia”, “sonodynamic therapy”, and “metal ion immune stimulation” (160). Ultimately, the distinct advantage of acoustic remodeling lies in its exceptional spatiotemporal precision, which can extend beyond the primary lesion to directly modulate immune organs. A pioneering study employed a FUS-responsive nanocomposite with mechanical luminescence properties. By applying FUS precisely to the draining lymph nodes, the nanomaterial emits light *in situ* under mechanical stress, thereby activating photosensitizers or other immune molecules. This results in the remote and non-invasive direct activation of the lymph nodes, referred to as the “immune command center”, thereby opening new dimensions for immune regulation (115).

In summary, acoustic immune remodeling provides comprehensive coverage of the entire DC activation pathway by precisely regulating cell death mechanisms, ingeniously designing nano-vaccines, proactively modifying the suppressive microenvironment, and directly intervening in immune centers. This approach offers a robust theoretical foundation and an innovative technological paradigm for igniting a widespread adaptive anti-tumor immune response.

### 3.5 Neutrophils: functional remodeling and carrier utilization transforming cancer promoters into anti-cancer pioneers

Neutrophils, the most abundant immune cells in the blood, exhibit notable duality within the TME. They can be polarized by tumors into the N2 phenotype, which promotes angiogenesis and suppresses T cell activity, or they may differentiate into the N1 anti-tumor phenotype, which directly kills tumors or assists T cell responses upon receiving specific signals (161–163). Acoustic immunomodulation strategies targeting neutrophil regulation have developed innovative approaches across various dimensions, including functional suppression, vector utilization, and direct armament.

Inhibiting the formation of neutrophil extracellular traps (NETs) constitutes a crucial strategy in acoustic remodeling. Research indicates that NETs can capture circulating tumor cells and promote distant metastasis, thereby serving as a key mechanism underlying the pro-cancer role of neutrophils. A notable study co-encapsulated the ultrasound sensitizer ICG and the PAD4 inhibitor GSK484, an enzyme critical for NET formation, within layered double hydroxide (LDH) nanosheets. Ultrasound-triggered PDT not only directly kills primary tumor cells but also effectively dismantles NET structures within the TME and the circulatory system. This dual action significantly inhibits distant metastasis following the detachment of tumor cells induced by the therapy, thereby achieving the objectives of “treating the primary site” and “preventing metastasis” (164). The use of neutrophils as “intelligent carriers” represents another significant advancement in acoustic immune remodeling. The sterile inflammation generated after FUS ablation can recruit numerous neutrophils to the residual tumor area. Based on this biological phenomenon, researchers have developed a neutrophil-mediated nanodrug delivery system (PLD@NEs). Following the co-culture of clinically approved liposomal doxorubicin (PLD) with neutrophils *in vitro*, PLD is phagocytosed by neutrophils and effectively transported to residual lesions that are typically difficult for drugs to penetrate within four hours post-FUS ablation, thereby greatly enhancing the efficiency of residual tumor clearance and offering a novel solution for adjuvant chemotherapy following clinical HIFU treatment (165).

Directly arming neutrophils as “cellular sonosensitizers” represents a groundbreaking technological advance in this field. Researchers have, for the first time, developed cRGD peptide-modified multilayer liposomes (C-ML/HPT/O2) loaded with oxygen-carrying PFC and the sonosensitizer temoporfin. These liposomes were co-cultured with live neutrophils to produce “acoustic neutrophils” (Acouscyte/O2). These engineered neutrophils not only retain their natural abilities for inflammatory chemotaxis and tumor recruitment but are also endowed with additional functions, including oxygen delivery, ultrasound imaging, fluorescent imaging, and PDT. In *in vivo* studies, Acouscyte/O2 selectively accumulates at tumor sites, releasing oxygen via PFC to alleviate tumor hypoxia. Simultaneously, ultrasound-triggered activation of temoporfin generates substantial amounts of ROS, inducing apoptosis in tumor cells (166). A more innovative strategy leverages the innate ability of neutrophils to cross the BBB, addressing the delivery challenges in central nervous system tumor treatment. Li et al. designed a persistent luminescent nanosensitizer consisting of a ZnGa2O4:Cr3+ (ZGO) core and a hollow TiO2 shell, loaded with paclitaxel (PTX) liposomes and anti-PD-1 antibodies, forming the ZGO@TiO2@ALP complex. By co-culturing with isolated neutrophils *in vitro*, ZGO@TiO2@ALP-NEs were prepared. These armed neutrophils respond to inflammatory signals from glioblastoma (GBM), traverse the BBB, and accumulate at tumor sites. Ultrasound irradiation triggers the TiO2 shell to produce ROS, disrupting the liposome structure and releasing PTX and anti-PD-1 antibodies. This not only facilitates a synergistic effect of chemotherapy and immunotherapy but also further induces a local inflammatory response, recruiting additional ZGO@TiO2@ALP-NEs to the tumor site, amplifying the therapeutic effect through positive feedback. This strategy improved the survival rate of GBM mice from 0% to 40% and established long-term immune surveillance, effectively preventing tumor recurrence (167).

Enhancing the effectiveness of PDT through neutrophil regulation represents a significant research direction. Studies have demonstrated that encapsulating midazolam in self-assembled nanomicelles can improve the therapeutic effect of PDT on gliomas by modulating the functional state of neutrophils. This strategy not only directly kills tumor cells but also enhances the TME by regulating the metabolism and functions of neutrophils, thereby providing new insights for the clinical translation of PDT (168). In addition to anti-tumor applications, acoustic regulation of neutrophils has shown promise in anti-infection therapies. Researchers have developed neutrophil membrane-coated antioxidant nanoparticles loaded with ciclopirox olamine, utilizing the inherent targeting ability of the neutrophil membrane to precisely protect the endothelial cells in the blood vessels of sepsis patients (169). Furthermore, multifunctional lipid-based nanocarriers have exhibited excellent dual antibacterial and anti-inflammatory activities in the treatment of methicillin-resistant *Staphylococcus aureus* (MRSA) bacteremia through modulation of the inflammatory response of neutrophils (170).

In summary, the regulatory strategies for acoustic immune remodeling targeting neutrophils have transitioned from basic functional suppression to multidimensional, precise control, clearly illustrating the paradigm shift from traditional cytotoxic therapies to cellular functional programming and behavioral guidance. These innovative strategies not only provide new perspectives for cancer treatment but also open new avenues for managing infectious diseases and inflammation-related disorders.

### 3.6 Neuro-immune axis reprogramming

The cholinergic anti-inflammatory pathway serves as a crucial link between the nervous system and the immune system. Acoustic technology, which offers unique advantages of non-invasiveness and remote control capabilities, has emerged as an innovative tool for the precise regulation of this pathway. A key application of acoustic immuno-reprogramming aims to achieve non-invasive and precise interventions for autoimmune diseases and metabolic disorders by targeting critical nodes of the neuro-immune axis, thereby establishing a new treatment paradigm for traditionally challenging diseases.

#### 3.6.1 Spleen-specific neuro-immune regulation: precise targeting of local anti-inflammation

LIFU specifically targets the innervation of the spleen, selectively activating cholinergic neurons through mechanical effects to induce localized ACh release from nerve endings. The high-affinity binding of ACh to the α7nAChR on splenic macrophages directly inhibits the nuclear translocation of the NF-κB signaling pathway, significantly diminishing the transcription and secretion of pro-inflammatory factors such as TNF-α, IL-1β, and IL-6 (171). This strategy offers the dual advantages of high organ specificity and independence from implant dependency, thereby circumventing the off-target toxicity associated with systemic medications. Consequently, it paves the way for innovative local anti-inflammatory treatments for systemic autoimmune diseases, including IBD and RA.

#### 3.6.2 Acoustic intervention of the gut-brain axis: a new mechanism for central regulation of peripheral inflammation

IBD models, tFUS targeting the dorsal motor nucleus of the vagus nerve (DMV) regulates peripheral inflammation along the gut-brain axis by enhancing vagal efferent function (172). The primary mechanism involves the transmission of central ultrasound signals via the vagus nerve to the intestinal wall nerve plexus, inhibiting the hyperactivation of resident intestinal macrophages and the release of pro-inflammatory factors, while simultaneously reducing neutrophil infiltration. This process significantly alleviates colonic mucosal inflammation and tissue damage. This cross-domain regulatory model, termed the “central target-peripheral effect,” overcomes the limitations of conventional local treatments for IBD and offers a novel strategy for the systemic regulation of complex intestinal inflammation.

#### 3.6.3 Systemic inflammation regulation of metabolic diseases: multi-target interventions for the repair of the neuro-immune network

Diabetic peripheral neuropathy (DPN) is the most common disabling complication of diabetes, with a core pathological mechanism closely linked to disturbances in neuro-immune interactions. To address this challenge, Luo et al. developed Ccl2 chemokine-targeted, ultrasound-responsive lipid nanoparticles (Ccr2@TA@LNP) (173). This nanoparticle platform precisely targets the neurons and immune cells in DPN-affected regions, enabling dual regulation upon ultrasound activation. First, it protects the ACh secretion function of nerve cells within a hyperglycemic microenvironment, thereby maintaining the activity of the cholinergic anti-inflammatory pathway. Second, it activates the JAK2/STAT3 signaling pathway in macrophages via the ACh-α7nAChR axis, promoting M2 macrophage polarization while suppressing Ccl2 secretion. By synergistically improving neuronal metabolic disorders, oxidative stress, and endoplasmic reticulum stress through a multi-target approach, this strategy not only provides an innovative solution for DPN treatment but also offers new technical references for the intervention of related complications, such as diabetic wound healing.

#### 3.6.4 Acoustodynamic intervention for central nervous system inflammation: cross-system treatment for cardiovascular diseases

Ventricular arrhythmia (VA) is the leading cause of sudden death following myocardial infarction (MI) and is closely linked to excessive activation of the sympathetic nervous system and the cascading amplification of central nervous inflammation post-MI. Hu et al. developed an autophagy-enhancing nanosensitizer named BBTD-TPA NPs, which is microscopically delivered to the paraventricular nucleus, a critical central region for cardiovascular homeostasis (174). Upon LIFU activation, this sensitizer specifically generates ROS and induces autophagy activation in microglia through the ROS-AMPK-mTOR signaling pathway, thereby inhibiting central nervous inflammatory responses and overactivity of the sympathetic nervous system. *In vivo* experiments demonstrate that this central-targeted acoustic intervention significantly reduces the incidence of malignant arrhythmias post-myocardial infarction, showcasing mitochondrial targeting, exceptional penetration depth, and favorable biocompatibility. This study represents the first confirmation of the feasibility of employing acoustic technology to treat peripheral cardiovascular diseases by modulating central neuro-immune pathways, thereby greatly expanding the clinical translational boundaries and cross-system therapeutic potential of acoustic immuno-reprogramming.

### 3.7 The immune cell network driven by acoustic remodeling

The preceding sections examined the regulation of specific immune cells and extracellular matrix components by acoustic-responsive nanoplatforms. However, the TME represents a complex ecosystem formed by interactions among diverse cells and molecules. The true strength of acoustic immune remodeling lies in its capacity to initiate a series of interconnected immune events through precisely timed and localized interventions, thereby creating a synergistic amplification network effect. This chapter aims to integrate these perspectives to elucidate how acoustic remodeling strategies orchestrate the complex synergy and crosstalk among immune cells from a systems-level viewpoint to achieve therapeutic benefits characterized by a supra-additive synergy.

#### 3.7.1 Dismantling the synergies within the immunosuppressive network

The tumor immunosuppressive network is characterized by multiple nodes and high redundancy, making treatment strategies that can simultaneously target several inhibitory pathways particularly advantageous. Acoustic-responsive nanoplatforms facilitate the multipoint, multilevel dismantling of this network through their multifunctional integration. The platform is designed to concurrently block key immunosuppressive signaling pathways and cell populations. Research has shown that drug microbubbles, loaded with PTX and PD-L1 antibodies, can enhance drug penetration through ultrasonic stimulation, while simultaneously reducing MDSCs and blocking the PD-L1 pathway, thus synergistically enhancing T cell function (108). Similar strategies include the co-delivery of sonosensitizers with TGF-β inhibitors (22) or adenosine A2a receptor antagonists with CTLA-4 antibodies (119), enabling a coordinated attack on multiple immunosuppressive pathways. Additionally, gene-level regulation, such as the delivery of miR-195 via nanobubbles, allows for the downregulation of tumor cell PD-L1 expression during SDT (175). Furthermore, modulating key metabolic molecules and core biological processes within the TME can profoundly reshape the immune landscape. One study developed mitochondria-targeted, ultrasound-responsive nanoparticles that co-deliver O_2_ and NO. Upon ultrasonic activation, the release of O_2_ alleviates tumor hypoxia, while NO simultaneously inhibits the function of Tregs and promotes the polarization of M2 macrophages to M1 macrophages, systematically reversing the immunosuppressive microenvironment (176). Another study targeted the autophagy process by using ultrasound-visible nanodroplets to deliver autophagy inhibitors (3-MA), enabling spatiotemporal control over tumor autophagy and reshaping the tumor inflammatory microenvironment, thereby enhancing the efficacy of subsequent immune checkpoint blockade (ICB) therapy (177).

#### 3.7.2 “*In Situ* Vaccine” effect: a programmed cascade of multicellular activation

The “*in situ* vaccine” strategy seeks to convert the TME into a center of immune activation. Its core lies in the use of an acoustic-responsive nanoplatform to trigger and orchestrate an immune activation cascade, involving the sequential engagement of various immune cells and tightly linked signaling pathways. This process is not a straightforward two-step procedure; rather, it is a precisely engineered, self-amplifying network effect.

Stage One: Initiation of the Immune Cascade—Simultaneous Release of Antigens, Adjuvants, and Danger Signals. The strategy begins not with isolated cell death, but with a complex event orchestrated by nanoplatforms that integrates multiple immune signals. For instance, one study employed a bio-inspired MOF nanoplatform (63), where ultrasound activation prompts the sonosensitizer components to induce ICD in tumor cells. This process leads to the exposure of calreticulin (CRT) on the cell membrane and the release of high mobility group box protein 1 (HMGB1) and ATP. These endogenous molecules serve as “danger signals”, immediately recruiting and activating DCs. Importantly, almost simultaneously, the exogenous adjuvant (R837) embedded in the MOF platform is released. This enables a single intervention by the platform to deliver three signals to the DCs: antigens from the dying tumor cells, “danger signals” from the ICD process, and a potent “adjuvant” from the platform, resulting in unprecedented activation of the DCs. Similarly, cascade targeting strategies—such as selectively restricting oxidative stress to the mitochondria of tumor cells (178) or utilizing manganese porphyrin liposomes to induce ICD—aim to enhance the intensity and quality of this initial composite signal, thus establishing the foundation for subsequent immune responses (179).

Stage Two: Immune Amplification Hub—DC-mediated Adaptive Immune Programmatic Mobilization. Fully activated DCs, which serve as a pivotal link between innate and adaptive immunity, initiate the subsequent phase of the cascade. They present the captured TAAs to naïve T cells in the draining lymph nodes, thereby activating and clonally expanding tumor-specific CTLs and CD4+ helper T cells. The efficiency and magnitude of this process are directly influenced by the quality of the stimuli received by the DCs in the first stage. In a GBM study, researchers designed a sequentially targeted sonodynamic nanovaccine that crosses the BBB via endothelial cells to target tumor cells, ultimately delivering the adjuvant (CpG) precisely to the activation site. This ensured that DCs could elicit a sufficiently robust T cell response, generating a systemic immune reaction capable of inhibiting distal metastases (180).

Stage Three: Effector Remodeling—T Cell-driven Immune Microenvironment Reprogramming. Activated CTLs return to the tumor site, where they not only directly kill residual tumor cells but also, more crucially, act as “battlefield environment remodelers” by secreting cytokines such as IFN-γ. IFN-γ upregulates the expression of MHC-I molecules on tumor cell surfaces, making them more susceptible to recognition and attack by other CTLs. Additionally, it acts as a key signal for the polarization of immunosuppressive M2 macrophages to pro-inflammatory M1 macrophages and further recruits other immune effector cells, such as NK cells, to the tumor site. By this point, a local response initiated by the acoustic nanoplatform *in situ* has evolved into a systemic anti-tumor immune response, involving multiple immune players, including DCs, T cells, macrophages, and NK cells, which is self-sustaining and continually amplified. To ensure the effective triggering of this cascade within the dense, heterogeneous environment of solid tumors, advanced delivery technologies designed to overcome physical barriers are crucial. For instance, to address the challenge of uneven drug distribution within large solid tumors, which impedes immune activation, researchers have developed intelligent microrobots controlled by “whirlwind acoustic vortices” (181). This system does not rely on passive diffusion but actively and controllably moves within the tumor tissue under the precise influence of an external acoustic field, evenly dispersing immune adjuvants (such as CpG) throughout the tumor. This method addresses the bottleneck of insufficient or uneven DC activation due to poor distribution. Another strategy focuses on designing biomimetic nanodrugs capable of responding to multiple biological barriers. One such “bio-barrier adaptive” platform integrates several mechanisms: its surface hybrid cell membrane camouflage ensures long circulation and tumor targeting; its core material undergoes charge reversal in the acidic TME to enhance cellular uptake; and ultimately, ultrasound cavitation effects facilitate the disruption of dense extracellular matrices and deep tissue penetration (182). Additionally, another study developed “volumetric nanodroplets” that undergo an acoustic phase transition under ultrasound stimulation, generating micron-sized bubbles *in situ (*183). The cavitation effect produced by this phase transition is more intense and controllable than traditional microbubbles, enabling efficient formation of “pores” (i.e., sonoporation) on the tumor cell membrane, significantly enhancing the intracellular delivery of immunotherapeutic drugs.

#### 3.7.3 Cell reprogramming and functional enhancement: immune cells as pivotal network control nodes

By reprogramming or functionally enhancing key immune cells within the TME, these cells can be transformed into pivotal nodes that regulate the entire immune network, thereby triggering a downstream domino effect.

Macrophage reprogramming serves as a typical example of this strategy. Studies have demonstrated that by delivering TLR7/8 agonists (R848) (111) or targeting CD47 (184) and Siglec-G (185) with siRNA, ultrasound-responsive nanoplatforms can effectively reprogram pro-tumor M2 macrophages into anti-tumor M1 phenotypes. This phenotypic conversion not only enhances the macrophages’ phagocytosis and antigen presentation capabilities but also increases the secretion of pro-inflammatory factors, which further recruit and activate T cells and NK cells, resulting in an amplification of the effect from single-cell reprogramming to multi-cellular network activation. Notably, the use of a mechanically responsive chemiluminescent nanoplatform driven by FUS has achieved closed-loop control and real-time feedback for engineered macrophages, representing a cutting-edge approach in this field (186).

Utilizing immune cells as therapeutic carriers or functionally enhancing them represents an advanced area of research. For instance, the “macrophage hitchhiking” strategy delivers drugs, such as β-caryophyllene, and exploits the tumor chemotaxis of macrophages to achieve targeted therapy (187). M1 macrophage membrane-camouflaged nanoplatforms effectively target tumors through their natural affinity, subsequently activating multiple immune responses via photoacoustic sensitization (188). Recent studies have also developed bio-hybrid microrobots based on neutrophils for oral targeted therapy in colorectal cancer (189). Additionally, the creation of “artificial killer cells” that simulate the cytotoxic functions of CTLs and NK cells upon ultrasound activation offers a novel engineered solution for cancer immunotherapy (190). The principles underlying these strategies have been successfully applied in sonodynamic and multi-immune synergistic therapies aimed at bacterial biofilm infections, illustrating their broad application potential (191). Furthermore, utilizing nanoplatforms to deliver the natural product fraxinellone can enhance the efficacy of therapeutic vaccinations by reshaping the balance of various immune cells within the TME (192).

## 4 Key challenges and future directions

**Figure 4:**
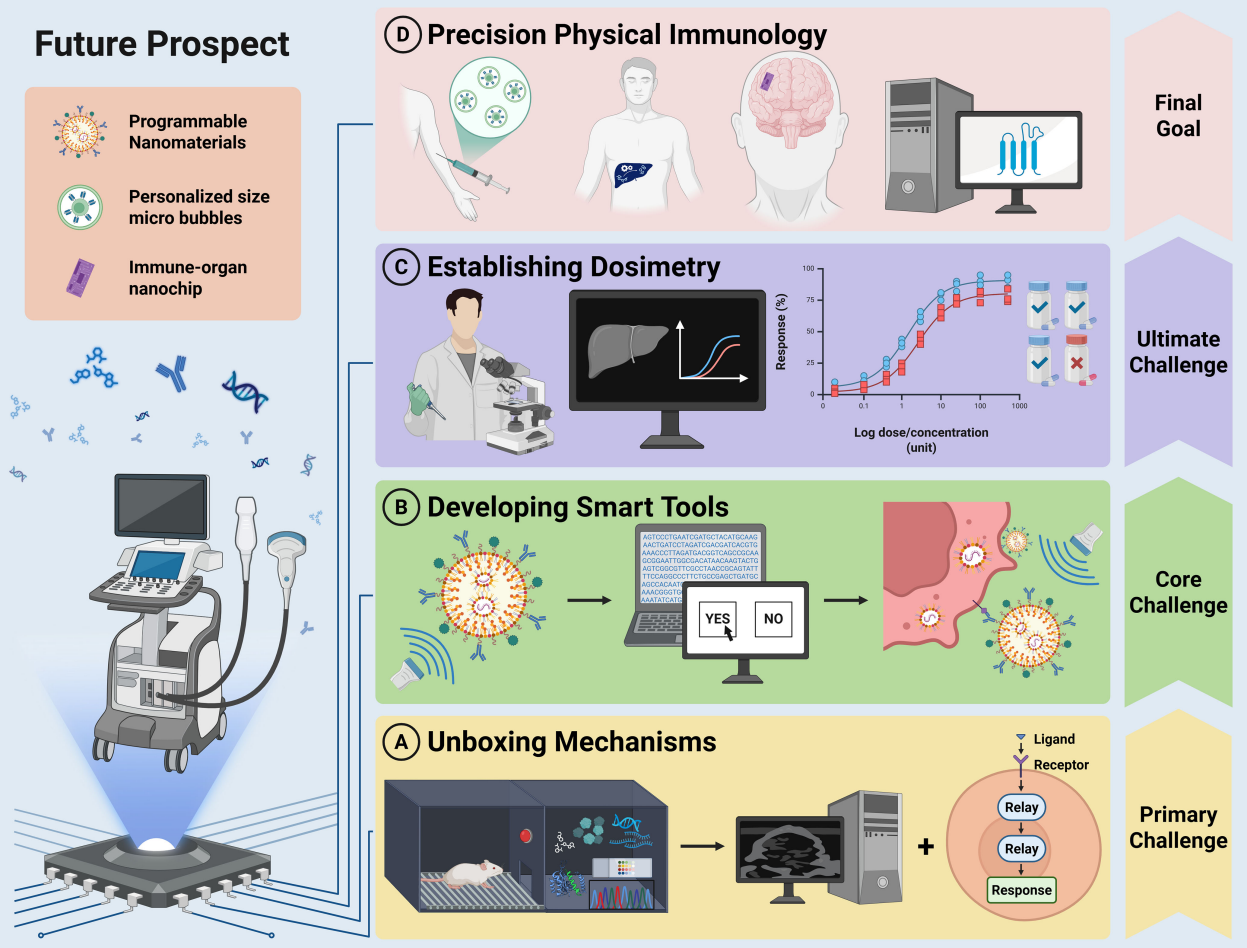
The key challenges and paths leading to clinical transformation (Created in https://BioRender.com): **(A)** Primary Challenge—Unboxing Mechanisms: Deciphering the mechanisms underlying the “black box” is essential for achieving interface visualization and establishing a standardized response map. **(B)** Core Challenge—Developing Smart Tools: The construction of intelligent models and feedback control systems will facilitate precise regulation of acoustic outputs. **(C)** Ultimate Challenge—Establishing Dosimetry: The development of a “dose-safety relationship map” is critical for quantifying the boundaries between treatment and toxicity. **(D)** Final Goal—Precision Physical Immunology: The objectives include designing personalized microbubbles, creating individualized digital twin organs, developing “immune-organ chips,” and establishing a repository for nanocarriers.

The “acoustic immune remodeling” paradigm offers unprecedented opportunities for remote interaction between physical energy and the immune system (193). However, to translate this promising concept into predictable, controllable, and broadly applicable clinical practices, we must address several major challenges inherent in its core principles. This chapter will systematically examine the key challenges in this field from both basic science and engineering perspectives, while also exploring its transformative future (**Figure 4**). Before fully harnessing this powerful technology, three fundamental scientific questions must be answered: What is it? How much is required? Is it safe? These questions logically map onto a sequence of challenges that escalate from fundamental science to clinical application, which we define as the “Primary Challenge” (decoding mechanisms), the “Core Challenge” (establishing dosimetry), and the “Ultimate Challenge” (ensuring safety).

### 4.1 Primary challenge: decoding the mechanisms of the “black box”

#### 4.1.1 Existing progress: key mechanistic discoveries

Recent studies have started to elucidate the molecular transducers that link sound energy to immune responses. Ultrasound-induced mechanical perturbations activate mechanosensitive channels (pannexin 1 [PANX1], Piezo1), resulting in Ca²^+^ influx that coordinates downstream immune signaling (194, 195). Focused ultrasound directly stimulates PANX1 channels, inducing intracellular calcium waves that enhance T cell activation and macrophage polarization (194, 196). Consequently, this Ca²^+^ surge drives the activation of the STING pathway in DCs, promoting the secretion of type I interferons and connecting innate immunity to adaptive immunity (197).

ICD markers—specifically, surface exposure of CRT, release of HMGB1, and secretion of extracellular ATP—have consistently been observed across various acoustic modalities, including HIFU, low-intensity ultrasound, and sonodynamic therapy. Pioneering research conducted by Sethuraman et al. demonstrated that CRT nanoparticles, when combined with FUS, can upregulate CRT expression, enhance HMGB1 release, and facilitate antigen release in melanoma, thereby significantly boosting anti-tumor immunity (198). Additionally, another study involving HIFU monitored the release of ICD markers through the optimization of acoustic parameters (199). Together, these findings establish sound energy as a biological signaling modality capable of reprogramming cellular immune status.

Furthermore, the pioneering research conducted by Hope et al. demonstrated that fluid shear stress enhances T cell activation via the mechanosensitive ion channel Piezo1 (196). The study established that shear stress-induced calcium signaling is reliant on Piezo1, and that GsMTx-4 (a Piezo1 inhibitor) can effectively block this effect, thereby providing direct evidence for ultrasound-mediated mechanotransduction in immune responses.

#### 4.1.2 Core bottlenecks: mechanistic ambiguities and the heterogeneity crisis

Yet the fundamental question—”What exactly are we activating, and through what pathways?”—remains inadequately answered due to two critical deficits:

First, mechanistic unpredictability: As demonstrated in pioneering studies, fine acoustic parameter control (e.g., FUS at 42°C vs. 46°C) elicits diametrically opposite immune outcomes—the former promoting pro-inflammatory M1 macrophages, the latter inducing anti-inflammatory M2 phenotypes (200). Moreover, introduction of ultrasound-responsive nanomaterials creates mechanistic ambiguity: it remains unclear how much immune activation results from mechanical effects (sonoporation, cavitation) versus chemical contributions (ROS-induced oxidative stress) (193, 201, 202). Non-classical cellular responses—such as microbubble-mediated rapid, non-apoptotic cell death (203) and acoustic-driven stem cell differentiation without chemical inducers (204)—further suggest unfamiliar biological pathways requiring clarification.

Secondly, defects in standardization: A systematic analysis reveals significant parameter heterogeneity. Martins et al. examined the variation in parameters reported in the literature, including ultrasound frequency ranges from 0.5 to 3.0 MHz (a 6-fold variation), intensity from 0.5 to 10.0 W/cm² (a 20-fold variation), duty cycles from 20% to 50%, and exposure times from 30 seconds to 10 minutes (also 20-fold variation), with minimal justification provided (205). This variation is further aggravated by the use of different animal models (heterotopic transplants versus isogenic models, yielding fundamentally distinct immune readouts), variations in the composition of nanoplatforms (microbubbles versus liposomes versus inorganic nanoparticles, each displaying different cavitation thresholds), and inconsistencies in timing (6-hour versus 7-day immune phenotype analyses that lack consensus). Such fragmentation obstructs meta-analysis and the formation of a unified mechanistic framework, raising a critical question: are different groups investigating the same phenomenon, or are they concealing fundamentally different acoustic-immune coupling mechanisms under a common nomenclature? As highlighted, even in conventional diagnostic ultrasound, the precise biological mechanisms remain a “mystery” for most practitioners (206), an issue that becomes amplified in complex therapeutic applications.

#### 4.1.3 Potential solutions: standardization as a prerequisite for disciplinary maturation

Resolving the “black box” demands coordinated strategies prioritizing consensus-building over premature unification:

First, establishing reporting standards: The field requires CONSORT-analogous guidelines specifying a minimal datasets: (1) comprehensive acoustic parameters (frequency, mechanical index, cavitation dose confirmed via passive detection); (2) nanoplatform characterization (size, ζ-potential, acoustic response); (3) standardized immune readouts at pre-defined timepoints (24h, 72h, 7d) including ICD markers, cytokine panels, and immune cell phenotyping. Recent CONSORT 2025 guidelines (207) and ultrasound reporting checklists (67) provide methodological blueprints requiring field-specific adaptation.

Second, multi-omics mechanistic mapping: Integrating single-cell transcriptomics, proteomics, and metabolomics across multiple acoustic modalities in standardized systems can construct comprehensive databases linking acoustic “inputs” to immune “outputs,” distinguishing core mechanisms from modality-specific epiphenomena. Stuart and Satija established a technical framework utilizing integrative single-cell analysis methods (208). When combined with real-time visualization techniques (such as FRET biosensors (209) and ultrafast microscopy (210)) and multiscale computational models that quantify energy transfer from the molecular to tissue levels (211, 212), these approaches are poised to facilitate the rational optimization of parameters.

Establishing “acoustic-immune biology” as a systematic sub-discipline hinges critically on achieving essential standardization milestones. The field is presently in a paradigm-exploratory phase, necessitating foundational consensus-building through multi-institutional collaboration, standardized protocols, and open data sharing. Only after demonstrating reproducible inter-laboratory validations under standardized conditions can acoustic immune reprogramming progress from heterogeneous observations to a formally recognized, mechanistically grounded discipline. This transformation requires both patience and intellectual humility; once realized, it is poised to generate transformative impacts.

### 4.2 Dosimetry challenge: bridging physical “input” to biological “output” existing progress: from physical metrics to biological dose indicators

The clinical translation of physical energy therapies fundamentally depends on dosimetry—yet the therapeutic ultrasound field urgently needs a comprehensive framework linking acoustic exposure to biological effects (213, 214). Pioneering advances have begun addressing this gap through cavitation-based dosimetry.

McDannold et al. established a correlation between the acoustic emissions of oscillating microbubbles, quantified by passive cavitation detection (PCD), and the permeability of the BBB (215). Subsequent studies demonstrated that cavitation dosage is a more reliable predictor of therapeutic effects than acoustic pressure applied in isolation. Pioneering research by Maciulevičius et al. confirmed that in ultrasound-enhanced bleomycin cytotoxicity, the correlation between cytotoxicity and the real-time measured “cavitation dosage” (R² = 0.89) significantly surpasses the correlation with acoustic intensity or mechanical index (216). This validates the concept that measuring direct effects (cavitation activity) is superior to measuring inputs (acoustic parameters) for biological dose quantification.

Furthermore, the systematic evaluation by Song et al. demonstrated that each acoustic parameter plays a critical role in disrupting the BBB: not only pressure, but also pulse length, duty cycle, and repetition frequency significantly influence the size, uniformity, and reversibility of the barrier opening. The gas volume dosage of microbubbles (rather than their size) shows a strong correlation with the degree of BBB permeability (R² = 0.90) (217). This emphasizes the significance of a comprehensive “acoustic prescription”—rather than relying on a single value, such as the mechanical index—in determining biological outcomes.

#### 4.2.1 Core bottlenecks: the “physical input–biological output” translation gap

Yet fundamental challenges persist: “How much acoustic energy produces specific immune responses, and what is the therapeutic window?” This question remains unanswered due to three critical gaps:

Firstly, inter-patient variability: due to tissue acoustic heterogeneity (including density, vascularization, and cellularity) and variations in physiological states, the same physical input yields markedly different biological outputs among patients (212, 218). A comprehensive analysis by Darmani et al. suggests that identical acoustic parameters in transcranial ultrasound neuromodulation can induce excitation or inhibition depending on the brain region and neuronal state, making the definition of a universal “neuromodulation dosage” extremely challenging (219). Similarly, in acoustic immunotherapy, the heterogeneity of the TME—characterized by a 10 to 100-fold variation in stromal density and immune infiltration among patients—may necessitate personalized dosage adjustments; however, personalized predictive biomarkers remain lacking.

Secondly, nonlinear dose-response relationships indicate that there is no straightforward linear correlation between acoustic pressure and biological effects. As demonstrated by Maciulevičius et al., cell viability displays a complex non-monotonic relationship with intensity, necessitating the integration of multi-parameter dose quantification that incorporates cavitation activity, thermal effects, and mechanical stress (216).

Third, lack of standardized biological endpoints: Should immune “dose” be defined by magnitude of tumor-infiltrating lymphocyte expansion? Peak systemic cytokine levels? Durability of immune memory? Different endpoints may require distinct acoustic prescriptions, yet no consensus exists on clinically relevant surrogate markers for immune activation intensity.

#### 4.2.2 Potential solutions: toward “precision acoustic medicine”

Addressing the dosimetry challenge requires coordinated strategies integrating real-time monitoring with patient-specific modeling:

Firstly, the standardization of biologically informed dose metrics is essential: beyond traditional physical doses (such as mechanical index and spatial peak temporal average intensity), the field must develop and validate biological dose metrics that accurately reflect immune processes. A promising approach involves standardizing cavitation dosage by quantifying the total cavitation activity within the treatment area using real-time PCD, thereby directly linking it to immune outcomes (including the release of ICD markers and levels of specific cytokines) (216, 220). Combining cavitation dosage with thermal dosage (cumulative equivalent minutes at 43°C) provides a complex dosimetry that incorporates multiple biophysical mechanisms.

Secondly, the development of patient-specific treatment plans through digital twins: By integrating medical imaging (such as CT-derived tissue density and MRI-derived vascular maps) with genomic data, AI-driven “computational clinical trials” can predict the propagation, attenuation, and biological effect hotspots of sound waves for each patient. Specifically, computed tomography (CT) provides high-resolution data on tissue density and atomic composition (via Hounsfield units), which are critical for estimating the acoustic impedance and nonlinearity parameter of tissues—key determinants of ultrasound reflection and harmonic generation. Conversely, magnetic resonance imaging (MRI), particularly techniques like MR elastography or T1/T2 mapping, can characterize tissue stiffness, viscosity, and perfusion. These mechanical and microstructural properties directly influence cavitation thresholds, thermal conductivity, and the efficiency of acoustic energy transfer to immune cells (221). Research conducted by Saratkar et al. and Vallée et al. illustrates how digital twins can synthesize multimodal data to enhance patient-specific treatment planning, predictive simulations, and personalized treatment optimization (222, 223). The feasibility of this direct mapping lies in established acoustic propagation models (e.g., the Khokhlov–Zabolotskaya–Kuznetsov equation) which can incorporate patient-specific maps of acoustic absorption and sound speed derived from CT and MRI. This integrated model enables pre-treatment dose optimization, similar to radiotherapy planning systems, effectively transitioning acoustic immunotherapy from empirical parameter selection to rational personalized prescriptions. This digital twin model enables pre-treatment dose optimization, similar to radiotherapy planning systems, effectively transitioning acoustic immunotherapy from empirical parameter selection to rational personalized prescriptions.

Thirdly, closed-loop adaptive control systems: Real-world implementation necessitates the integration of precise “ammunition” with real-time feedback. Martinez et al. demonstrated that size-separated microbubbles (2 μm vs. 6 μm) exhibit predictable cavitation behavior at specific frequencies, with monodisperse microbubbles facilitating more accurate cavitation control (224). In conjunction with real-time feedback obtained through PCD, Sun et al. successfully achieved closed-loop feedback control in a rat glioma model for the first time, maintaining stable cavitation and suppressing inertial cavitation through real-time monitoring (225). This approach allows for reliable and non-invasive delivery of predetermined drug concentrations, providing proof of concept for clinical applications.

Crucially, these dosimetry advances must be integrated with the standardization roadmap outlined in Section 4.1. Achieving reproducible dose-response relationships requires consensus on: (1) which biological endpoints define therapeutic “dose”; (2) how to measure these endpoints reliably across institutions; (3) acceptable variability thresholds for declaring “equivalent doses.” Only through coordinated multi-institutional validation studies—applying standardized acoustic protocols with comprehensive PCD monitoring and unified immune phenotyping—can the field establish the dose-response curves foundational to evidence-based acoustic immunotherapy.

The intrinsic theranostic nature of ultrasound is pivotal for overcoming the dosimetry challenge. Real-time ultrasound imaging provides anatomical context, while advanced techniques like PCD or contrast-enhanced imaging can monitor the therapeutic process itself (e.g., microbubble cavitation, drug release). This fusion of anatomical and functional data enables a closed-loop feedback system. The ultrasound device can thus automatically adjust its parameters in response to the observed biological effects, ensuring the delivery of the prescribed ‘biological dose’ to the target while sparing surrounding healthy tissue. This seamless integration of imaging and therapy is the ultimate embodiment of the spatiotemporal control promised by acoustic immune reprogramming.

### 4.3 Safety challenge: navigating the immune “double-edged sword”

#### 4.3.1 Existing progress: established safety profiles in controlled applications

Decades of clinical experience with ultrasound therapy have established baseline safety parameters. FDA-approved microbubble contrast agents exhibit a favorable safety profile. A retrospective analysis by Dézsi et al. on sulfur hexafluoride microbubbles (SonoVue) involving a large patient cohort revealed that the incidence of severe adverse events is below 0.01%, primarily presenting as mild complement activation-related pseudoallergy (CARPA), characterized by transient back pain or flushing (226). The pig CARPA model developed by Szebeni et al. offers a highly sensitive and reproducible method for assessing the safety of nanomedicines (227).

Similarly, a study by Downs et al. in non-human primates indicated that when parameters are maintained within established thresholds, repeated FUS treatments for BBB opening over 20 months do not produce any long-term adverse effects (228).

Additionally, cavitation monitoring technology facilitates real-time safety assessments. A novel safety index developed by Novell et al. that is based on pulse-internal superharmonic cavitation monitoring provides quantitative thresholds to differentiate safe BBB opening from hemorrhagic injury. These advancements establish proof of concept: under appropriate monitoring, controlled acoustic exposure presents an acceptable risk profile (229).

#### 4.3.2 Core bottlenecks: the multifactorial risk “triple threat”

Yet translating acoustic immunotherapy to complex clinical environments confronts three interconnected safety challenges that compound non-linearly:

Firstly, the dilemma of cavitation control: The transition from stable cavitation (therapeutic, controlled membrane permeabilization) to inertial cavitation (destructive bubble collapse producing micro-jets, shock waves, and localized high temperatures) is inherently random and context-dependent. Church’s classic studies indicate that near the inertial cavitation threshold, even a 5-10% increase in sound pressure can lead to a significant rise in collapse pressure and tissue damage (230). In the context of BBB opening applications, reviews by Gandhi et al. and Angolano et al. highlight that exceeding the cavitation threshold carries risks of microbleeding, edema, and neuronal damage—potentially catastrophic consequences in immunotherapy, where systemic immune activation may exacerbate local inflammation (231, 232). The physical parameter space between “effective” and “harmful” remains poorly defined across various tissue types and patient-specific acoustic environments.

Secondly, the immunogenicity of nanoplatforms: The toxicity of nanoparticles is not an inherent material property; rather, it dynamically arises from the interaction between physicochemical parameters (such as size, shape, surface charge, and composition) and the biological environment. A review by Corbo et al. indicates that protein corona formation fundamentally alters the biological identity of nanoparticles, influencing cytotoxicity and immune responses (233). Rampado et al. further elucidate that the protein corona can either mask or enhance the targeting capabilities and immunogenicity of nanoparticles (234).

Lipid nanoparticles (LNPs), which are increasingly being explored for ultrasound-responsive drug delivery, can circumvent conventional immune recognition and directly activate the complement system, triggering CARPA within minutes. Szebeni et al. noted that in the analysis of rare hypersensitivity reactions to mRNA vaccines, clinical manifestations vary substantially from mild rashes to life-threatening allergic reactions, demonstrating significant individual variability (235). This suggests that even “biocompatible” nanomaterials may pose unpredictable immunogenic risks when applied to heterogeneous patient populations.

Thirdly, the convergence of cytokine storms: Acoustic immunotherapy aims to induce controlled “therapeutic inflammation” to activate anti-tumor immunity. However, when the risk of CARPA pre-activation coincides with the substantial release of inflammatory mediators (IL-1β, IL-18) induced by sonodynamic therapy, this “coordinated immune activation” may escalate into a catastrophic and life-threatening cytokine release syndrome (CRS). Reviews by Wang et al. and Zhang et al. indicate that 20-30% of patients undergoing CAR-T therapy develop severe CRS, necessitating intensive care (236, 237). A critical question remains: at what intensity of immune activation do the treatment benefits transition into systemic toxicity? Current preclinical models lack predictive capability for human susceptibility to CRS.

#### 4.3.3 Potential solutions: risk mitigation through intelligent monitoring and rational design

Navigating the immune double-edged sword demands proactive, multi-level safeguards:

Firstly, intelligent cavitation control systems: Future ultrasound platforms must evolve into “perception-response” systems that integrate real-time PCD with adaptive feedback control. Research by Novell et al. and Sun et al. demonstrates that by quantitatively linking cavitation signals to biological effects (such as drug release kinetics and BBB permeability), the system can automatically adjust acoustic output when inertial cavitation signals exceed safety thresholds, thereby maintaining energy delivery within an efficient and low-toxicity treatment window (225, 229). Clinical implementation requires multi-institutional validation studies to standardize cavitation dosage thresholds across various tissue types and patient populations.

Secondly, the design of immuno-programmable nanoplatforms: By moving beyond traditional concepts of “biological inertness” and “biocompatibility,” next-generation platforms must integrate systematic immunoengineering. High-throughput screening for constructing a comprehensive library of nanocarriers with varying parameters, combined with multi-omics analyses (such as proteomics and metabolomics), can elucidate the structure-activity relationships that link physicochemical properties to immunological events, including CARPA, inflammasome activation, and macrophage polarization (226, 238). This approach facilitates the rational design of platforms aimed at achieving either “immune silent” delivery (via suppression of CARPA and complement activation through surface modification) or selective pathway activation (e.g., STING but not the NLRP3 inflammasome), depending on therapeutic goals.

Thirdly, predictive patient stratification and early warning systems: There is considerable variability in individual susceptibility to CRS. Pre-treatment biomarkers—elevated baseline inflammatory markers (C-reactive protein >10 mg/L) and genetic polymorphisms in cytokine genes (variants of IL-6 and TNF-α)—can identify high-risk patients who require dosage adjustments or exclusion from early clinical trials (239, 240). Real-time immune monitoring through liquid biopsies that measure serum cytokine panels (IL-6, TNF-α, IFN-γ) at 2, 6, and 24 hours post-treatment facilitates ultra-early detection of CRS (within a 24- to 72-hour window), triggering preventive interventions (such as tocilizumab and corticosteroids) before the syndrome escalates.

Crucially, safety advances must integrate with the dosimetry framework (Section 4.2) and standardization roadmap (Section 4.1). Defining therapeutic windows requires consensus on: (1) acceptable adverse event rates for specific indications; (2) standardized cavitation dose thresholds across institutions; (3) immune activation biomarker panels qualifying as safety signals. Only through coordinated translational research—combining physics-based cavitation control, immunology-informed nanodesign, and real-world patient monitoring—can acoustic immunotherapy safely fulfill its transformative potential.

### 4.4 Translation challenge: from laboratory to clinic

#### 4.4.1 Existing progress

The clinical translation of acoustic immunotherapy reveals significant platform-dependent differences. A systematic review by Izadifar et al. indicates that HIFU has received regulatory approval for tissue ablation, with over 300,000 procedures conducted worldwide (241); however, the thermal destruction paradigm on which it relies fundamentally differs from mechanisms of immune modulation. Ashar et al. observed that sub-ablative HIFU applications for mechanical tumor destruction and BBB opening have progressed to multicenter trials, yet regulatory approval has not been secured while awaiting evidence of efficacy (242).

Microbubble-mediated drug delivery employs FDA/EMA-approved ultrasound contrast agents (Definity, SonoVue, Sonazoid), which were originally developed for diagnostic echocardiography. Their repurposing for therapeutic cavitation-enhanced extravasation has facilitated the initiation of phase I/II trials aimed at enhancing chemotherapy delivery, although published efficacy data remain limited. A review by Gong and Dai highlights that sonodynamic therapy progresses the slowest due to the absence of approved sonosensitizers and unaddressed dosimetry standardization issues (243).

#### 4.4.2 Core bottlenecks

##### 4.4.2.1 The EPR heterogeneity crisis

The EPR effect, which underpins the concept of passive nanoparticle targeting, demonstrates significant clinical unpredictability. A groundbreaking meta-analysis by Golombek et al. revealed that while preclinical mouse models consistently show an accumulation of 3-10% of the injected dose per gram of tumor tissue (%ID/g), the median clinical delivery is only 0.7% ID/g (95% CI: 0.3-1.5%) (244). Hua et al. further confirmed that inter-patient variability can reach up to 50-fold (245). This heterogeneity arises from variations in tumor vascular architecture, including vascular density, pericyte coverage, and basement membrane integrity, as well as dynamic IFP that opposes convective transport and patient-specific factors such as prior treatment-induced vascular normalization or fibrosis.

In ultrasound-triggered systems, baseline uncertainties interact multiplicatively with ultrasound-specific variables through three mechanisms. Early drug leakage can result from non-targeted acoustic exposure due to diagnostic ultrasound or arterial pulsation, as well as formulation instability under fluctuating tumor conditions, such as an acidic pH of 6.5-6.8 and localized hyperthermia. Context-dependent release kinetics vary with real-time microbubble concentrations, which are regulated by cardiac output and pulmonary filtration, tissue acoustic impedance, influenced by fat content, fibrosis, and edema, and the formation of standing waves. Rapid reticuloendothelial clearance, combined with a circulation half-life of less than 6 hours for conventional lipid nanoparticles, creates a narrow time window for coordinating ultrasound with peak intratumoral accumulation (246).

These factors result in significant differences in bioavailability among patients, despite the use of standardized dosing regimens, which directly undermines the pharmacokinetic-pharmacodynamic (PK-PD) relationship. A review by Entzian and Aigner indicates that this unpredictability contributed to the failure of key trials for thermosensitive liposomal doxorubicin (ThermoDox, LTSL-DOX), wherein the specified trigger in the protocol did not yield consistent therapeutic concentrations. Although Phase II signals were promising, regulatory approval was ultimately hindered (247). The primary challenge lies in establishing a reproducible dose-response relationship when nanoparticle delivery and acoustic triggering vary independently and non-linearly. Patients with low baseline EPR may require higher acoustic intensities to achieve equivalent drug release; however, such escalation may exceed the cavitation safety threshold. Conversely, premature ultrasound application before peak accumulation or delayed application after clearance fundamentally limits the achievable concentration, regardless of intensity.

##### 4.4.2.2 Device-drug synergy optimization without validated frameworks

Unlike radiation oncology with established dose-response relationships enabling systematic treatment planning, acoustic immunotherapy operates within under-characterized multidimensional parameter space. Biological outcomes depend on interdependent variables spanning nanoparticle formulation (size, surface chemistry, acoustic responsiveness), ultrasound exposure (frequency, intensity, pulse parameters, duration), microbubble properties (size distribution, shell composition), and temporal coordination relative to nanoparticle pharmacokinetics.

Device architectural heterogeneity compounds this challenge. Contemporary systems span single-element focused transducers (0.5–2 MHz) to multi-element phased arrays (128–1024 channels) and extracorporeal versus interstitial applicators, generating multi-center reproducibility crises. Nominally equivalent acoustic protocols produce divergent biological outcomes due to differences in beam focusing quality, standing wave patterns, or tissue-specific energy absorption. The interaction between device-specific acoustic fields and patient-specific tumor acoustic properties creates combinatorial complexity beyond systematic exploration through traditional dose-escalation designs.

Critically, the coupling between pharmacokinetic variability and acoustic optimization is non-linear and patient-specific. The absence of validated dosimetry frameworks analogous to radiation oncology’s biologically effective dose (BED) leaves clinicians without rational guidance for individualized optimization. Current population-averaged dosing paradigms fail to account for multiplicative interactions between EPR variability and acoustic parameter responsiveness.

#### 4.4.3 Potential solutions

##### 4.4.3.1 Precision medicine approaches for EPR heterogeneity

Addressing the unpredictability of the EPR effect necessitates patient stratification using functional imaging biomarkers. Golombek et al. proposed that pre-treatment PET imaging utilizing radiolabeled reporting nanoparticles (e.g., 89Zr-labeled liposomes that replicate therapeutic carrier characteristics) can quantitatively assess individual tumor EPR capabilities (244). Patients exhibiting inadequate passive accumulation (<1% ID/g) can be redirected to alternative treatment options or EPR enhancement strategies, such as anti-angiogenic normalization or transient increases in blood pressure. In contrast, patients demonstrating robust EPR receive dosages determined by quantitative uptake values, similar to the workflow for radiopharmaceuticals.

Real-time adaptive control integrating population PK-PD modeling with ultrasound feedback represents the critical translational pathway. Bayesian algorithms incorporating patient covariates (body surface area, hepatic function, prior therapy) and sparse early blood sampling predict optimal ultrasound timing windows corresponding to peak intratumoral nanoparticle concentrations. Simultaneous PCD quantifying acoustic emissions confirms effective mechanical disruption while maintaining safety margins below inertial cavitation thresholds (Section 4.2), enabling closed-loop dose optimization.

Engineering acoustically inert nanoformulations with sharp activation thresholds minimizes premature leakage while preserving on-demand release. High-transition-temperature lipids remain stable at 37°C but release cargo above 42°C achievable with FUS. Perfluorocarbon nanodroplets undergo acoustic droplet vaporization only above mechanical index >0.4, providing binary switching between passive circulation and active extravasation states.

##### 4.4.3.2 Systems standardization for device-drug optimization

Device-drug synergy optimization fundamentally requires establishing a unified dosimetry framework analogous to radiation oncology’s absorbed dose paradigm. Current acoustic immunotherapy lacks the conceptual equivalent of Gray (Gy) as a universally quantifiable biological dose unit. Reported parameters such as mechanical index or spatial-peak temporal-average intensity describe physical energy delivery but fail to account for tissue-specific acoustic energy absorption, nanoparticle concentration variability, or temporal coordination effects. This absence prevents meaningful cross-institutional outcome comparisons and rational treatment planning.

Establishing such a framework demands integration of three validated metrics into a composite biological dose descriptor. First, cavitation dose quantified through PCD (Section 4.2) provides real-time measurement of mechanical tissue disruption. Second, nanoparticle delivery efficiency measured via baseline EPR imaging establishes patient-specific delivery capacity. Third, spatiotemporal drug release kinetics captured through pharmacokinetic modeling predicts achievable intratumoral concentrations. A validated composite metric integrating these components, analogous to radiotherapy’s BED formulation accounting for dose-rate and fractionation effects, would enable systematic dose-response relationship establishment across heterogeneous patient populations and device platforms.

Implementation requires pre-competitive industry consortia establishing consensus reporting standards mandating modular transducer architectures with calibrated acoustic output specifications (Section 4.1) and standardized nanoparticle characterization protocols enabling cross-institutional data harmonization. Multi-institutional registry studies prospectively correlating composite biological dose with clinical outcomes would populate training datasets for machine learning algorithms identifying optimal parameter combinations. Critically, such registries must capture not only treatment parameters but also failure modes, as current publication bias toward positive results obscures understanding of parameter ranges producing subtherapeutic or toxic outcomes.

Regulatory acceleration depends on qualifying imaging biomarkers as surrogate endpoints validated through prospective demonstration that early changes correlate with long-term clinical benefit. Contrast-enhanced ultrasound quantifying drug extravasation kinetics or PET imaging of immune cell trafficking represent promising candidates requiring structured validation through FDA’s Biomarker Qualification Program. Platform trial designs evaluating multiple formulations within master protocols sharing control arms improve efficiency while embedded Bayesian adaptive randomization enables real-time optimization based on accumulating efficacy and safety data.

These translational solutions necessitate vertical integration with standardization frameworks (Section 4.1), dosimetry metrics (Section 4.2), and safety monitoring architectures (Section 4.3). Coordinated research integrating patient stratification, real-time adaptive dosing, and validated device standards is essential for overcoming barriers impeding clinical realization.

## 5 Conclusion

The essence of acoustic immunoremodulation lies in transforming the “physical field” from a secondary role in diagnostic imaging to the primary function of remotely programming and regulating immune responses in deep tissues. It offers modern medicine a non-invasive “acoustic scalpel” capable of overcoming physiological barriers, establishing cross-scale regulatory pathways between the physical, chemical, and biological domains, and enabling precise cellular and molecular-level reconstruction of disease fortresses that have long eluded conventional drugs. This not only signals the emergence of a new therapeutic paradigm that seamlessly integrates physics and immunology, but also opens vast opportunities for Precision Physical Immunology. Looking ahead, the development of biological acoustics dosimetry, therapeutic safety, and the resolution of the mechanism black box will be pivotal in advancing the field of “Precision Physical Immunology”.

## References

[1] Yuan X Xiao Y Yu D . Turn cold tumors hot by reprogramming the tumor microenvironment. Nat Biotechnol. (2025) 43:466–70. doi: 10.1038/s41587-025-02597-w, PMID: 40229361

[2] He Y Aoun M Xu Z Holmdahl R . Shift in perspective: autoimmunity protecting against rheumatoid arthritis. Ann Rheum Dis. (2024) 83:550–5. doi: 10.1136/ard-2023-225237, PMID: 38413169

[3] Li M Sun X Zeng L Sun A Ge J . Metabolic homeostasis of immune cells modulates cardiovascular diseases. Res (Wash D C). (2025) 8:679. doi: 10.34133/research.0679, PMID: 40270694 PMC12015101

[4] Neurath MF Sands BE Rieder F . Cellular immunotherapies and immune cell depleting therapies in inflammatory bowel diseases: the next magic bullet? Gut. (2024) 74:9–14. doi: 10.1136/gutjnl-2024-332919, PMID: 39025492 PMC11671923

[5] Erickson MA Wilson ML Banks WA . *In vitro* modeling of blood-brain barrier and interface functions in neuroimmune communication. Fluids Barriers CNS. (2020) 17:26. doi: 10.1186/s12987-020-00187-3, PMID: 32228633 PMC7106666

[6] Boyman O Comte D Spertini F . Adverse reactions to biologic agents and their medical management. Nat Rev Rheumatol. (2014) 10:612–27. doi: 10.1038/nrrheum.2014.123, PMID: 25112605

[7] Duffy AG Greten TF . Immunological off-target effects of standard treatments in gastrointestinal cancers. Ann Oncol. (2014) 25:24–32. doi: 10.1093/annonc/mdt349, PMID: 24201974 PMC3868318

[8] Moreb JS . Off-target effects of carfilzomib that cause cardiotoxicity. Blood. (2019) 133:626–8. doi: 10.1182/blood-2018-12-889758, PMID: 30765494

[9] Papamichael K Afif W Drobne D Dubinsky MC Ferrante M Irving PM . Therapeutic drug monitoring of biologics in inflammatory bowel disease: unmet needs and future perspectives. Lancet Gastroenterol Hepatol. (2022) 7:171–85. doi: 10.1016/S2468-1253(21)00223-5, PMID: 35026171 PMC10187071

[10] Liston DR Davis M . Clinically relevant concentrations of anticancer drugs: A guide for nonclinical studies. Clin Cancer Res. (2017) 23:3489–98. doi: 10.1158/1078-0432.CCR-16-3083, PMID: 28364015 PMC5511563

[11] Ramos-Casals M Siso-Almirall A . Immune-related adverse events of immune checkpoint inhibitors. Ann Intern Med. (2024) 177:ITC17–32. doi: 10.7326/AITC202402200, PMID: 38346306

[12] Durbin SM Zubiri L Perlman K Wu CY Lim T Grealish K . Late-onset immune-related adverse events after immune checkpoint inhibitor therapy. JAMA Netw Open. (2025) 8:e252668. doi: 10.1001/jamanetworkopen.2025.2668, PMID: 40146104 PMC11950896

[13] Cho KI Yu J Hayashi T Han SH Koh KK . Strategies to overcome residual risk during statins era. Circ J. (2019) 83:1973–9. doi: 10.1253/circj.CJ-19-0624, PMID: 31391351

[14] Sanchez-Dengra B Gonzalez-Alvarez I Bermejo M Gonzalez-Alvarez M . Access to the CNS: strategies to overcome the BBB. Int J Pharm. (2023) 636:122759. doi: 10.1016/j.ijpharm.2023.122759, PMID: 36801479

[15] Luo H Cao G Luo C Tan D Vong CT Xu Y . Emerging pharmacotherapy for inflammatory bowel diseases. Pharmacol Res. (2022) 178:106146. doi: 10.1016/j.phrs.2022.106146, PMID: 35227890

[16] Wang S Hossack JA Klibanov AL . From anatomy to functional and molecular biomarker imaging and therapy: ultrasound is safe, ultrafast, portable, and inexpensive. Invest Radiol. (2020) 55:559–72. doi: 10.1097/RLI.0000000000000675, PMID: 32776766 PMC10290890

[17] Tian M Xin X Wu R Guan W Zhou W . Advances in intelligent-responsive nanocarriers for cancer therapy. Pharmacol Res. (2022) 178:106184. doi: 10.1016/j.phrs.2022.106184, PMID: 35301111

[18] Zhang Q Kuang G Li W Wang J Ren H Zhao Y . Stimuli-responsive gene delivery nanocarriers for cancer therapy. Nanomicro Lett. (2023) 15:44. doi: 10.1007/s40820-023-01018-4, PMID: 36752939 PMC9908819

[19] Zhuang F Xiang H Huang B Chen Y . Ultrasound-triggered cascade amplification of nanotherapy. Adv Mater. (2023) 35:e2303158. doi: 10.1002/adma.202303158, PMID: 37222084

[20] Arrieta VA Gould A Kim KS Habashy KJ Dmello C Vazquez-Cervantes GI . Ultrasound-mediated delivery of doxorubicin to the brain results in immune modulation and improved responses to PD-1 blockade in gliomas. Nat Commun. (2024) 15:4698. doi: 10.1038/s41467-024-48326-w, PMID: 38844770 PMC11156895

[21] Baez A Singh D He S Hajiaghayi M Gholizadeh F Darlington PJ . Immunomodulation of human T cells by microbubble-mediated focused ultrasound. Front Immunol. (2024) 15:1486744. doi: 10.3389/fimmu.2024.1486744, PMID: 39502696 PMC11534865

[22] Liao Z Liao H Luo Y Chen S Chen M He J . Red blood cell membrane-camouflaged nanoparticles for synergistic sonodynamic therapy and TGF-beta inhibition to reprogram immunosuppressive tumor microenvironment. ACS Appl Mater Interfaces. (2025) 17:41680–95. doi: 10.1021/acsami.5c07008, PMID: 40637538

[23] Thanh Nguyen T Asakura Y Koda S Yasuda K . Dependence of cavitation, chemical effect, and mechanical effect thresholds on ultrasonic frequency. Ultrason Sonochem. (2017) 39:301–6. doi: 10.1016/j.ultsonch.2017.04.037, PMID: 28732949

[24] Nowicki A . Safety of ultrasonic examinations; thermal and mechanical indices. Med Ultrason. (2020) 22:203–10. doi: 10.11152/mu-2372, PMID: 32399527

[25] Wu P Wang X Lin W Bai L . Acoustic characterization of cavitation intensity: A review. Ultrason Sonochem. (2022) 82:105878. doi: 10.1016/j.ultsonch.2021.105878, PMID: 34929549 PMC8799601

[26] Stride EP Coussios CC . Cavitation and contrast: the use of bubbles in ultrasound imaging and therapy. Proc Inst Mech Eng H. (2010) 224:171–91. doi: 10.1243/09544119JEIM622, PMID: 20349814

[27] Fant C Lafond M Rogez B Castellanos IS Ngo J Mestas JL . *In vitro* potentiation of doxorubicin by unseeded controlled non-inertial ultrasound cavitation. Sci Rep. (2019) 9:15581. doi: 10.1038/s41598-019-51785-7, PMID: 31666639 PMC6821732

[28] Coussios CC Roy RA . Applications of acoustics and cavitation to noninvasive therapy and drug delivery. Annu Rev Fluid Mechanics. (2008) 40:395–420. doi: 10.1146/annurev.fluid.40.111406.102116

[29] Hoogenboom M Eikelenboom D den Brok MH Heerschap A Futterer JJ Adema GJ . Mechanical high-intensity focused ultrasound destruction of soft tissue: working mechanisms and physiologic effects. Ultrasound Med Biol. (2015) 41:1500–17. doi: 10.1016/j.ultrasmedbio.2015.02.006, PMID: 25813532

[30] Khokhlova TD Haider YA Maxwell AD Kreider W Bailey MR Khokhlova VA . Dependence of boiling histotripsy treatment efficiency on HIFU frequency and focal pressure levels. Ultrasound Med Biol. (2017) 43:1975–85. doi: 10.1016/j.ultrasmedbio.2017.04.030, PMID: 28641910 PMC5547902

[31] Baek H Yang Y Pacia CP Xu L Yue Y Bruchas MR . Mechanical and mechanothermal effects of focused ultrasound elicited distinct electromyographic responses in mice. Phys Med Biol. (2021) 66:10.1088/1361-6560/ac08b. doi: 10.1088/1361-6560/ac08b1, PMID: 34098539 PMC8822499

[32] Church CC Labuda C Nightingale K . A theoretical study of inertial cavitation from acoustic radiation force impulse imaging and implications for the mechanical index. Ultrasound Med Biol. (2015) 41:472–85. doi: 10.1016/j.ultrasmedbio.2014.09.012, PMID: 25592457 PMC4297318

[33] Xu Z Ludomirsky A Eun LY Hall TL Tran BC Fowlkes JB . Controlled ultrasound tissue erosion. IEEE Trans Ultrason Ferroelectr Freq Control. (2004) 51:726–36. doi: 10.1109/tuffc.2004.1308731, PMID: 15244286 PMC2669757

[34] Pahk KJ Shin CH Bae IY Yang Y Kim SH Pahk K . Boiling histotripsy-induced partial mechanical ablation modulates tumour microenvironment by promoting immunogenic cell death of cancers. Sci Rep. (2019) 9:9050. doi: 10.1038/s41598-019-45542-z, PMID: 31227775 PMC6588624

[35] Khokhlova VA Fowlkes JB Roberts WW SChade GR Xu Z Khokhlova TD . Histotripsy methods in mechanical disintegration of tissue: towards clinical applications. Int J Hyperthermia. (2015) 31:145–62. doi: 10.3109/02656736.2015.1007538, PMID: 25707817 PMC4448968

[36] Nandi T Kop BR Naftchi-Ardebili K Stagg CJ Pauly KB Verhagen L . Biophysical effects and neuromodulatory dose of transcranial ultrasonic stimulation. Brain Stimul. (2025) 18:659–64. doi: 10.1016/j.brs.2025.02.019, PMID: 40054576 PMC12464205

[37] Bader KB Holland CK . Gauging the likelihood of stable cavitation from ultrasound contrast agents. Phys Med Biol. (2013) 58:127–44. doi: 10.1088/0031-9155/58/1/127, PMID: 23221109 PMC4467591

[38] Xu Z Hall TL Vlaisavljevich E Lee FT Jr. Histotripsy: the first noninvasive, non-ionizing, non-thermal ablation technique based on ultrasound. Int J Hyperthermia. (2021) 38:561–75. doi: 10.1080/02656736.2021.1905189, PMID: 33827375 PMC9404673

[39] Yang Y Li Q Guo X Tu J Zhang D . Mechanisms underlying sonoporation: Interaction between microbubbles and cells. Ultrason Sonochem. (2020) 67:105096. doi: 10.1016/j.ultsonch.2020.105096, PMID: 32278246

[40] van Elburg B Deprez J van den Broek M De Smedt SC Versluis M Lajoinie G . Dependence of sonoporation efficiency on microbubble size: An *in vitro* monodisperse microbubble study. J Control Release. (2023) 363:747–55. doi: 10.1016/j.jconrel.2023.09.047, PMID: 37778466

[41] Theek B Baues M Ojha T Mockel D Veettil SK Steitz J . Sonoporation enhances liposome accumulation and penetration in tumors with low EPR. J Control Release. (2016) 231:77–85. doi: 10.1016/j.jconrel.2016.02.021, PMID: 26878973 PMC5404719

[42] Castle J Kotopoulis S Forsberg F . Sonoporation for augmenting chemotherapy of pancreatic ductal adenocarcinoma. Methods Mol Biol. (2020) 2059:191–205. doi: 10.1007/978-1-4939-9798-5_9, PMID: 31435922 PMC7418147

[43] Thim EA Kitelinger LE Rivera-Escalera F Mathew AS Elliott MR Bullock TNJ . Focused ultrasound ablation of melanoma with boiling histotripsy yields abscopal tumor control and antigen-dependent dendritic cell activation. Theranostics. (2024) 14:1647–61. doi: 10.7150/thno.92089, PMID: 38389838 PMC10879863

[44] Pavlov VA Tracey KJ . The cholinergic anti-inflammatory pathway. Brain Behav Immun. (2005) 19:493–9. doi: 10.1016/j.bbi.2005.03.015, PMID: 15922555

[45] Yu K Niu X Krook-Magnuson E He B . Intrinsic functional neuron-type selectivity of transcranial focused ultrasound neuromodulation. Nat Commun. (2021) 12:2519. doi: 10.1038/s41467-021-22743-7, PMID: 33947867 PMC8097024

[46] Chu PC Huang CS Chang PK Chen RS Chen KT Hsieh TH . Weak ultrasound contributes to neuromodulatory effects in the rat motor cortex. Int J Mol Sci. (2023) 24:2578. doi: 10.3390/ijms24032578, PMID: 36768901 PMC9917173

[47] Seasons GM Pellow C Kuipers HF Pike GB . Ultrasound and neuroinflammation: immune modulation via the heat shock response. Theranostics. (2024) 14:3150–77. doi: 10.7150/thno.96270, PMID: 38855178 PMC11155413

[48] Gigliotti JC Huang L Bajwa A Ye H Mace EH Hossack JA . Ultrasound modulates the splenic neuroimmune axis in attenuating AKI. J Am Soc Nephrol. (2015) 26:2470–81. doi: 10.1681/ASN.2014080769, PMID: 25644106 PMC4587697

[49] Yang J Liao M Wu Z Liu X Zheng Z Wang W . Perfluorohexane nanodroplet-assisted mechanical high intensity focused ultrasound cavitation: A strategy for hepatocellular carcinoma treatment. Acta Biomater. (2025) 195:297–308. doi: 10.1016/j.actbio.2025.01.061, PMID: 39894325

[50] Liu HL Hsieh HY Lu LA Kang CW Wu MF Lin CY . Low-pressure pulsed focused ultrasound with microbubbles promotes an anticancer immunological response. J Transl Med. (2012) 10:221. doi: 10.1186/1479-5876-10-221, PMID: 23140567 PMC3543346

[51] Zhang M Hu W Cai C Wu Y Li J Dong S . Advanced application of stimuli-responsive drug delivery system for inflammatory arthritis treatment. Mater Today Bio. (2022) 14:100223. doi: 10.1016/j.mtbio.2022.100223, PMID: 35243298 PMC8881671

[52] Yang F Lv J Huang Y Ma W Yang Z . A supramolecular assembly strategy for the treatment of rheumatoid arthritis with ultrasound-augmented inflammatory microenvironment reprograming. Biomaterials. (2025) 316:123006. doi: 10.1016/j.biomaterials.2024.123006, PMID: 39675142

[53] Li W Song Y Liang X Zhou Y Xu M Lu Q . Mutual-reinforcing sonodynamic therapy against Rheumatoid Arthritis based on sparfloxacin sonosensitizer doped concave-cubic rhodium nanozyme. Biomaterials. (2021) 276:121063. doi: 10.1016/j.biomaterials.2021.121063, PMID: 34391020

[54] Darrow DP . Focused ultrasound for neuromodulation. Neurotherapeutics. (2019) 16:88–99. doi: 10.1007/s13311-018-00691-3, PMID: 30488340 PMC6361056

[55] Yang H Sun Y Wei J Xu L Tang Y Yang L . The effects of ultrasound-targeted microbubble destruction (UTMD) carrying IL-8 monoclonal antibody on the inflammatory responses and stability of atherosclerotic plaques. BioMed Pharmacother. (2019) 118:109161. doi: 10.1016/j.biopha.2019.109161, PMID: 31545223

[56] Yang L Chen L Fang Y Ma S . Downregulation of GSK-3beta expression via ultrasound-targeted microbubble destruction enhances atherosclerotic plaque stability in New Zealand rabbits. Ultrasound Med Biol. (2021) 47:710–22. doi: 10.1016/j.ultrasmedbio.2020.11.002, PMID: 33261913

[57] Su Y Xu C Li K Wang B Chen J Liu L . TGF-beta1 and TIMP1 double directional rAAV targeted by UTMD in atherosclerotic vulnerable plaque. Exp Ther Med. (2017) 13:1465–9. doi: 10.3892/etm.2017.4101, PMID: 28413493 PMC5377323

[58] Liu J Chen Y Wang G Jin Q Sun Z Lv Q . Improving acute cardiac transplantation rejection therapy using ultrasound-targeted FK506-loaded microbubbles in rats. Biomater Sci. (2019) 7:3729–40. doi: 10.1039/c9bm00301k, PMID: 31403142

[59] Wang Z Jiang S Li S Yu W Chen J Yu D . Targeted galectin-7 inhibition with ultrasound microbubble targeted gene therapy as a sole therapy to prevent acute rejection following heart transplantation in a Rodent model. Biomaterials. (2020) 263:120366. doi: 10.1016/j.biomaterials.2020.120366, PMID: 32950914

[60] Bao H Dai L Wang H Jiang T . Ultrasound-targeted sirolimus-loaded microbubbles improves acute rejection of heart transplantation in rats by inhibiting TGF-beta1-Smad signaling pathway, promoting autophagy and reducing inflammation. Int J Pharm X. (2024) 8:100300. doi: 10.1016/j.ijpx.2024.100300, PMID: 39624342 PMC11609676

[61] Ai C Sun X Xiao S Guo L Shang M Shi D . CAFs targeted ultrasound-responsive nanodroplets loaded V9302 and GLULsiRNA to inhibit melanoma growth via glutamine metabolic reprogramming and tumor microenvironment remodeling. J Nanobiotechnology. (2023) 21:214. doi: 10.1186/s12951-023-01979-z, PMID: 37420266 PMC10329298

[62] Wang X Shi Z Luo J Zeng Y He L Chen L . Ultrasound improved immune adjuvant delivery to induce DC maturation and T cell activation. J Control Release. (2022) 349:18–31. doi: 10.1016/j.jconrel.2022.06.054, PMID: 35780954

[63] Lu Z Bai S Jiang Y Wu S Xu D Zhang J . Amplifying dendritic cell activation by bioinspired nanometal organic frameworks for synergistic sonoimmunotherapy. Small. (2022) 18:e2203952. doi: 10.1002/smll.202203952, PMID: 36148843

[64] Wegierak D Nittayacharn P Cooley MB Berg FM Kosmides T Durig D . Nanobubble contrast enhanced ultrasound imaging: A review. Wiley Interdiscip Rev Nanomed Nanobiotechnol. (2024) 16:e2007. doi: 10.1002/wnan.2007, PMID: 39511794 PMC11567054

[65] Sheth M Knight C Wu Q Vasilyeva A Upadhyay A Bau L . Size matters: Micro- versus nanobubbles in ultrasound imaging and therapy. Sci Adv. (2025) 11:eads2177. doi: 10.1126/sciadv.ads2177, PMID: 40668930 PMC12266114

[66] Hawley JJ Allen SL Thompson DM Schwarz AJ Tranquart FJM . Commercially available ultrasound contrast agents: factors contributing to favorable outcomes with ultrasound-mediated drug delivery and ultrasound localization microscopy imaging. Invest Radiol. (2025) 60:813–22. doi: 10.1097/RLI.0000000000001197, PMID: 40262129

[67] Sirsi SR Borden MA . State-of-the-art materials for ultrasound-triggered drug delivery. Adv Drug Delivery Rev. (2014) 72:3–14. doi: 10.1016/j.addr.2013.12.010, PMID: 24389162 PMC4041842

[68] Batchelor DVB Armistead FJ Ingram N Peyman SA McLaughlan JR Coletta PL . The influence of nanobubble size and stability on ultrasound enhanced drug delivery. Langmuir. (2022) 38:13943–54. doi: 10.1021/acs.langmuir.2c02303, PMID: 36322191 PMC9671049

[69] Su C Ren X Nie F Li T Lv W Li H . Current advances in ultrasound-combined nanobubbles for cancer-targeted therapy: a review of the current status and future perspectives. RSC Adv. (2021) 11:12915–28. doi: 10.1039/d0ra08727k, PMID: 35423829 PMC8697319

[70] Xing Z Wang J Ke H Zhao B Yue X Dai Z . The fabrication of novel nanobubble ultrasound contrast agent for potential tumor imaging. Nanotechnology. (2010) 21:145607. doi: 10.1088/0957-4484/21/14/145607, PMID: 20220227

[71] Chen X Xu L Chen C Huang Q Hu J . Recent advances in ultrasound-targeted nanobubbles combined with cancer immunotherapy: Mechanisms, applications, and challenges. Fundam Res. (2024) 367:135–47. doi: 10.1016/j.fmre.2024.10.017 PMC12869779 41647554

[72] Nittayacharn P Abenojar E Cooley M Berg F Counil C Sojahrood AJ . Efficient ultrasound-mediated drug delivery to orthotopic liver tumors - Direct comparison of doxorubicin-loaded nanobubbles and microbubbles. bioRxiv. (2023). doi: 10.1101/2023.09.01.555196, PMID: 38237687 PMC11700473

[73] Terlikowska KM Dobrzycka B Terlikowski SJ . Modifications of nanobubble therapy for cancer treatment. Int J Mol Sci. (2024) 25:7292. doi: 10.3390/ijms25137292, PMID: 39000401 PMC11242568

[74] Patel PB Latt S Ravi K Razavi M . Clinical applications of micro/nanobubble technology in neurological diseases. Biomimetics. (2024) 9:645. doi: 10.3390/biomimetics9100645, PMID: 39451851 PMC11506587

[75] Sharma D Petchiny TN Czarnota GJ . A promising therapeutic strategy of combining acoustically stimulated nanobubbles and existing cancer treatments. Cancers (Basel) 16(18). (2024) 16:3181. doi: 10.3390/cancers16183181, PMID: 39335153 PMC11431001

[76] Lee JY Carugo D Crake C Owen J de Saint Victor M Seth A . Nanoparticle-loaded protein-polymer nanodroplets for improved stability and conversion efficiency in ultrasound imaging and drug delivery. Adv Mater. (2015) 27:5484–92. doi: 10.1002/adma.201502022, PMID: 26265592

[77] Qin D Zou Q Lei S Wang W Li Z . Predicting initial nucleation events occurred in a metastable nanodroplet during acoustic droplet vaporization. Ultrason Sonochem. (2021) 75:105608. doi: 10.1016/j.ultsonch.2021.105608, PMID: 34119737 PMC8207230

[78] Hou J Zhou J Chang M Bao G Xu J Ye M . LIFU-responsive nanomedicine enables acoustic droplet vaporization-induced apoptosis of macrophages for stabilizing vulnerable atherosclerotic plaques. Bioact Mater. (2022) 16:120–33. doi: 10.1016/j.bioactmat.2022.02.022, PMID: 35386311 PMC8958425

[79] Zhao Y Qin D Chen J Hou J Ilovitsh T Wan M . On-demand regulation and enhancement of the nucleation in acoustic droplet vaporization using dual-frequency focused ultrasound. Ultrason Sonochem. (2022) 90:106224. doi: 10.1016/j.ultsonch.2022.106224, PMID: 36368292 PMC9649937

[80] Amin M Lammers T Ten Hagen TLM . Temperature-sensitive polymers to promote heat-triggered drug release from liposomes: Towards bypassing EPR. Adv Drug Delivery Rev. (2022) 189:114503. doi: 10.1016/j.addr.2022.114503, PMID: 35998827

[81] Cressey P Amrahli M So PW Gedroyc W Wright M Thanou M . Image-guided thermosensitive liposomes for focused ultrasound enhanced co-delivery of carboplatin and SN-38 against triple negative breast cancer in mice. Biomaterials. (2021) 271:120758. doi: 10.1016/j.biomaterials.2021.120758, PMID: 33774525

[82] Wei D Sun Y Zhu H Fu Q . Stimuli-responsive polymer-based nanosystems for cancer theranostics. ACS Nano. (2023) 17:23223–61. doi: 10.1021/acsnano.3c06019, PMID: 38041800

[83] Handa M Singh A Flora SJS Shukla R . Stimuli-responsive polymeric nanosystems for therapeutic applications. Curr Pharm Des. (2022) 28:910–21. doi: 10.2174/1381612827666211208150210, PMID: 34879797

[84] Wu S Wang T Xu H . Regulating heterogeneous catalysis of gold nanoparticles with polymer mechanochemistry. ACS Macro Lett. (2020) 9:1192–7. doi: 10.1021/acsmacrolett.0c00451, PMID: 35638615

[85] Li S He N Wu X Chen F Xue Q Li S . Characteristics of ultrasound-driven barium titanate nanoparticles and the mechanism of action on solid tumors. Int J Nanomed. (2024) 19:12769–91. doi: 10.2147/ijn.S491816, PMID: 39624116 PMC11610387

[86] Chen H Zhou X Gao Y Zheng B Tang F Huang J . Recent progress in development of new sonosensitizers for sonodynamic cancer therapy. Drug Discov Today. (2014) 19:502–9. doi: 10.1016/j.drudis.2014.01.010, PMID: 24486324

[87] Mehta M Bui TA Yang X Aksoy Y Goldys EM Deng W . Lipid-based nanoparticles for drug/gene delivery: an overview of the production techniques and difficulties encountered in their industrial development. ACS Mater Au. (2023) 3:600–19. doi: 10.1021/acsmaterialsau.3c00032, PMID: 38089666 PMC10636777

[88] Ciofani G Danti S D’Alessandro D Ricotti L Moscato S Bertoni G . Enhancement of neurite outgrowth in neuronal-like cells following boron nitride nanotube-mediated stimulation. ACS Nano. (2010) 4:6267–77. doi: 10.1021/nn101985a, PMID: 20925390

[89] Marino A Arai S Hou Y Sinibaldi E Pellegrino M Chang YT . Piezoelectric nanoparticle-assisted wireless neuronal stimulation. ACS Nano. (2015) 9:7678–89. doi: 10.1021/acsnano.5b03162, PMID: 26168074 PMC9003232

[90] Pu Y Yin H Dong C Xiang H Wu W Zhou B . Sono-controllable and ROS-sensitive CRISPR-cas9 genome editing for augmented/synergistic ultrasound tumor nanotherapy. Adv Mater. (2021) 33:e2104641. doi: 10.1002/adma.202104641, PMID: 34536041

[91] Beebe MA Paredes-Sabja D Kociolek LK Rodriguez C Sorg JA . Phenotypic analysis of various Clostridioides difficile ribotypes reveals consistency among core processes. bioRxiv. (2025) 91:e0096425. doi: 10.1101/2025.01.10.632434, PMID: 40552806 PMC12285255

[92] Zhu S Xu W Li H Sun Z Zhu Y Liu W . Metal-organic frameworks activate the cGAS-STING pathway for cancer immunotherapy. J Nanobiotechnology. (2025) 23:578. doi: 10.1186/s12951-025-03669-4, PMID: 40841898 PMC12369232

[93] Chen WH Luo GF Zhang XZ . Recent advances in subcellular targeted cancer therapy based on functional materials. Adv Mater. (2019) 31:e1802725. doi: 10.1002/adma.201802725, PMID: 30260521

[94] Cai M Fu T Zhu R Hu P Kong J Liao S . An iron-based metal-organic framework nanoplatform for enhanced ferroptosis and oridonin delivery as a comprehensive antitumor strategy. Acta Pharm Sin B. (2024) 14:4073–86. doi: 10.1016/j.apsb.2024.05.015, PMID: 39309488 PMC11413704

[95] Lu K Aung T Guo N Weichselbaum R Lin W . Nanoscale metal-organic frameworks for therapeutic, imaging, and sensing applications. Adv Mater. (2018) 30:e1707634. doi: 10.1002/adma.201707634, PMID: 29971835 PMC6586248

[96] Khulood MT Jijith US Naseef PP Kallungal SM Geetha VS Pramod K . Advances in metal-organic framework-based drug delivery systems. Int J Pharm. (2025) 673:125380. doi: 10.1016/j.ijpharm.2025.125380, PMID: 39988215

[97] Huang C Shao N Huang Y Chen J Wang D Hu G . Overcoming challenges in the delivery of STING agonists for cancer immunotherapy: A comprehensive review of strategies and future perspectives. Mater Today Bio. (2023) 23:100839. doi: 10.1016/j.mtbio.2023.100839, PMID: 38024837 PMC10630661

[98] Liu Y Crowe WN Wang L Lu Y Petty WJ Habib AA . An inhalable nanoparticulate STING agonist synergizes with radiotherapy to confer long-term control of lung metastases. Nat Commun. (2019) 10:5108. doi: 10.1038/s41467-019-13094-5, PMID: 31704921 PMC6841721

[99] Zhang F Lu G Wen X Li F Ji X Li Q . Magnetic nanoparticles coated with polyphenols for spatio-temporally controlled cancer photothermal/immunotherapy. J Control Release. (2020) 326:131–9. doi: 10.1016/j.jconrel.2020.06.015, PMID: 32580043

[100] Rastegari E Hsiao YJ Lai WY Lai YH Yang TC Chen SJ . An update on mesoporous silica nanoparticle applications in nanomedicine. Pharmaceutics. (2021) 13:1067. doi: 10.3390/pharmaceutics13071067, PMID: 34371758 PMC8309088

[101] Manzano M Vallet-Regí M . Mesoporous silica nanoparticles for drug delivery. Advanced Funct Mater. (2019) 30:1902634. doi: 10.1002/adfm.201902634

[102] Qian X Zheng Y Chen Y . Micro/nanoparticle-augmented sonodynamic therapy (SDT): breaking the depth shallow of photoactivation. Adv Mater. (2016) 28:8097–129. doi: 10.1002/adma.201602012, PMID: 27384408

[103] Li X Lovell JF Yoon J Chen X . Clinical development and potential of photothermal and photodynamic therapies for cancer. Nat Rev Clin Oncol. (2020) 17:657–74. doi: 10.1038/s41571-020-0410-2, PMID: 32699309

[104] Chen J Luo H Liu Y Zhang W Li H Luo T . Oxygen-self-produced nanoplatform for relieving hypoxia and breaking resistance to sonodynamic treatment of pancreatic cancer. ACS Nano. (2017) 11:12849–62. doi: 10.1021/acsnano.7b08225, PMID: 29236476

[105] Son S Kim JH Wang X Zhang C Yoon SA Shin J . Multifunctional sonosensitizers in sonodynamic cancer therapy. Chem Soc Rev. (2020) 49:3244–61. doi: 10.1039/c9cs00648f, PMID: 32337527

[106] Zhu P Chen Y Shi J . Nanoenzyme-augmented cancer sonodynamic therapy by catalytic tumor oxygenation. ACS Nano. (2018) 12:3780–95. doi: 10.1021/acsnano.8b00999, PMID: 29613770

[107] McHale AP Callan JF Nomikou N Fowley C Callan B . Sonodynamic therapy: concept, mechanism and application to cancer treatment. Adv Exp Med Biol. (2016) 880:429–50. doi: 10.1007/978-3-319-22536-4_22, PMID: 26486350

[108] Zheng J Huang J Zhang L Wang M Xu L Dou X . Drug-loaded microbubble delivery system to enhance PD-L1 blockade immunotherapy with remodeling immune microenvironment. Biomater Res. (2023) 27:9. doi: 10.1186/s40824-023-00350-5, PMID: 36759928 PMC9909878

[109] Han H Kim D Jang Y Seo M Kim K Lee JB . Focused ultrasound-triggered chemo-gene therapy with multifunctional nanocomplex for enhancing therapeutic efficacy. J Control Release. (2020) 322:346–56. doi: 10.1016/j.jconrel.2020.03.041, PMID: 32243982

[110] Huang FY Lei J Sun Y Yan F Chen B Zhang L . Induction of enhanced immunogenic cell death through ultrasound-controlled release of doxorubicin by liposome-microbubble complexes. Oncoimmunology. (2018) 7:e1446720. doi: 10.1080/2162402X.2018.1446720, PMID: 29900064 PMC5993503

[111] Han S Wang W Wang S Yang T Zhang G Wang D . Tumor microenvironment remodeling and tumor therapy based on M2-like tumor associated macrophage-targeting nano-complexes. Theranostics. (2021) 11:2892–916. doi: 10.7150/thno.50928, PMID: 33456579 PMC7806477

[112] Zhang N Foiret J Kheirolomoom A Liu P Feng Y Tumbale S . Optimization of microbubble-based DNA vaccination with low-frequency ultrasound for enhanced cancer immunotherapy. Adv Ther (Weinh). (2021) 4:2100033. doi: 10.1002/adtp.202100033, PMID: 34632048 PMC8494128

[113] Shi C Zhang Y Yang H Dong T Chen Y Xu Y . Ultrasound-targeted microbubble destruction-mediated Foxp3 knockdown may suppress the tumor growth of HCC mice by relieving immunosuppressive Tregs function. Exp Ther Med. (2018) 15:31–8. doi: 10.3892/etm.2017.5421, PMID: 29387180 PMC5769241

[114] Liang M Kang X Liu H Zhang L Wang T Ye M . Ultrasound-energized OX40L-expressing biohybrid for multidimensional mobilization of sustained T cell-mediated antitumor immunity and potent sono-immunotherapy. J Am Chem Soc. (2025) 147:13833–50. doi: 10.1021/jacs.5c02025, PMID: 40200836

[115] Xu S Li X Hu Q Zhang J Li R Meng L . Focused ultrasound-responsive nanocomposite with near-infrared II mechanoluminescence for spatiotemporally selective immune activation in lymph nodes. Chemistry. (2024) 30:e202304066. doi: 10.1002/chem.202304066, PMID: 38289154

[116] Wu Y Li J Shu L Tian Z Wu S Wu Z . Ultrasound combined with microbubble mediated immunotherapy for tumor microenvironment. Front Pharmacol. (2024) 15:1304502. doi: 10.3389/fphar.2024.1304502, PMID: 38487163 PMC10937735

[117] Zhang J Sun L Jiang L Xie X Wang Y Wu R . Regulation of CTLs/tregs via highly stable and ultrasound-responsive cerasomal nano-modulators for enhanced colorectal cancer immunotherapy. Adv Sci (Weinh). (2024) 11:e2400485. doi: 10.1002/advs.202400485, PMID: 38552151 PMC11165532

[118] Chen L Li H Liu J Wang Y Zhang S . Hollow mesoporous carbon nanospheres derived from metal-organic frameworks for efficient sono-immunotherapy against pancreatic cancer. Cyborg Bionic Syst. (2025) 6:247. doi: 10.34133/cbsystems.0247, PMID: 40352815 PMC12062583

[119] Li Z Zhang B Duan S Liu R Wang Y Wang Y . Ultrasound-activated nanovesicles for adenosine exhaustion and immune checkpoint blockade in cancer immunotherapy. J Control Release. (2025) 385:113988. doi: 10.1016/j.jconrel.2025.113988, PMID: 40582643

[120] Qin P Han T Yu ACH Xu L . Mechanistic understanding the bioeffects of ultrasound-driven microbubbles to enhance macromolecule delivery. J Control Release. (2018) 272:169–81. doi: 10.1016/j.jconrel.2018.01.001, PMID: 29305924

[121] Song Y Chen J Zhang C Xin L Li Q Liu Y . Mechanosensitive channel Piezo1 induces cell apoptosis in pancreatic cancer by ultrasound with microbubbles. iScience. (2022) 25:103733. doi: 10.1016/j.isci.2022.103733, PMID: 35118354 PMC8792083

[122] Zhu Q He Y Dong XX Xu Y Zhang Y Liu Z . Microbubble enhanced ultrasound with low mechanical index promotes therapeutic angiogenesis in hind limb ischemia mouse model. Med Phys. (2025) 52:1706–16. doi: 10.1002/mp.17539, PMID: 39666574

[123] Feng Y Qiu D He Y Jin H Chen L Xi F . Effect of ultrasound combined with microbubbles therapy on tumor hypoxic microenvironment. Front Pharmacol. (2024) 15:1502349. doi: 10.3389/fphar.2024.1502349, PMID: 39872052 PMC11769831

[124] Zhao Z Lin X Zhang L Liu X Wang Q Shi Y . Lipidated methotrexate microbubbles: A promising rheumatoid arthritis theranostic medicine manipulated via ultrasonic irradiation. J BioMed Nanotechnol. (2021) 17:1293–304. doi: 10.1166/jbn.2021.3105, PMID: 34446133

[125] Guo R Wang L Huang J Pang H Wang L Zhu B . Ultrasound-targeted microbubble destruction-mediated cell-mimetic nanodrugs for treating rheumatoid arthritis. ACS Biomater Sci Eng. (2023) 9:3670–9. doi: 10.1021/acsbiomaterials.3c00475, PMID: 37184981

[126] Wu H He Y Wu H Zhou M Xu Z Xiong R . Near-infrared fluorescence imaging-guided focused ultrasound-mediated therapy against Rheumatoid Arthritis by MTX-ICG-loaded iRGD-modified echogenic liposomes. Theranostics. (2020) 10:10092–105. doi: 10.7150/thno.44865, PMID: 32929336 PMC7481417

[127] Do HD Marie C Bessoles S Dhotel H Seguin J Larrat B . Combination of thermal ablation by focused ultrasound, pFAR4-IL-12 transfection and lipidic adjuvant provide a distal immune response. Explor Target Antitumor Ther. (2022) 3:398–413. doi: 10.37349/etat.2022.00090, PMID: 36046055 PMC9400762

[128] Shu H Lv W Ren ZJ Li H Dong T Zhang Y . Ultrasound-mediated PLGA-PEI nanobubbles carrying STAT6 siRNA enhances NSCLC treatment via repolarizing tumor-associated macrophages from M2 to M1 phenotypes. Curr Drug Delivery. (2024) 21:1114–27. doi: 10.2174/1567201820666230724151545, PMID: 37491853

[129] Luo T Sun J Zhu S He J Hao L Xiao L . Ultrasound-mediated destruction of oxygen and paclitaxel loaded dual-targeting microbubbles for intraperitoneal treatment of ovarian cancer xenografts. Cancer Lett. (2017) 391:1–11. doi: 10.1016/j.canlet.2016.12.032, PMID: 28043912

[130] Xu Y Liu R Yang H Qu S Qian L Dai Z . Enhancing photodynamic therapy efficacy against cancer metastasis by ultrasound-mediated oxygen microbubble destruction to boost tumor-targeted delivery of oxygen and renal-clearable photosensitizer micelles. ACS Appl Mater Interfaces. (2022) 14:25197–208. doi: 10.1021/acsami.2c06655, PMID: 35615986

[131] Ding M Zhang Y Yu N Zhou J Zhu L Wang X . Augmenting immunogenic cell death and alleviating myeloid-derived suppressor cells by sono-activatable semiconducting polymer nanopartners for immunotherapy. Adv Mater. (2023) 35:e2302508. doi: 10.1002/adma.202302508, PMID: 37165741

[132] Chen Z Liu W Yang Z Luo Y Qiao C Xie A . Sonodynamic-immunomodulatory nanostimulators activate pyroptosis and remodel tumor microenvironment for enhanced tumor immunotherapy. Theranostics. (2023) 13:1571–83. doi: 10.7150/thno.79945, PMID: 37056565 PMC10086211

[133] Zheng J Sun Y Long T Yuan D Yue S Zhang N . Sonosensitizer nanoplatform-mediated sonodynamic therapy induced immunogenic cell death and tumor immune microenvironment variation. Drug Delivery. (2022) 29:1164–75. doi: 10.1080/10717544.2022.2058653, PMID: 35393920 PMC9004507

[134] Dong L Liu D Zhang J Ling Y Li X Ou J . Ultrasound-triggered NPC1L1-targeting nanobubbles for remodeling the tumor microenvironment in pancreatic cancer chemoimmunotherapy. ACS Appl Mater Interfaces. (2025) 17:34965–81. doi: 10.1021/acsami.5c01194, PMID: 40478203 PMC12186220

[135] Zhao Y Shi D Guo L Shang M Sun X Meng D . Ultrasound targeted microbubble destruction-triggered nitric oxide release via nanoscale ultrasound contrast agent for sensitizing chemoimmunotherapy. J Nanobiotechnology. (2023) 21:35. doi: 10.1186/s12951-023-01776-8, PMID: 36717899 PMC9885630

[136] Joshi RS Kanugula SS Sudhir S Pereira MP Jain S Aghi MK . The role of cancer-associated fibroblasts in tumor progression. Cancers (Basel). (2021) 13:1399. doi: 10.3390/cancers13061399, PMID: 33808627 PMC8003545

[137] Snipstad S Vikedal K Maardalen M Kurbatskaya A Sulheim E Davies CL . Ultrasound and microbubbles to beat barriers in tumors: Improving delivery of nanomedicine. Adv Drug Delivery Rev. (2021) 177:113847. doi: 10.1016/j.addr.2021.113847, PMID: 34182018

[138] Einen C Snipstad S Wesche HF Nordlund V Devold EJ Amini N . Impact of the tumor microenvironment on delivery of nanomedicine in tumors treated with ultrasound and microbubbles. J Control Release. (2025) 378:656–70. doi: 10.1016/j.jconrel.2024.12.037, PMID: 39701458

[139] Mai Z Lin Y Lin P Zhao X Cui L . Modulating extracellular matrix stiffness: a strategic approach to boost cancer immunotherapy. Cell Death Dis. (2024) 15:307. doi: 10.1038/s41419-024-06697-4, PMID: 38693104 PMC11063215

[140] Curley CT Mead BP Negron K Kim N Garrison WJ Miller GW . Augmentation of brain tumor interstitial flow via focused ultrasound promotes brain-penetrating nanoparticle dispersion and transfection. Sci Adv. (2020) 6:eaay1344. doi: 10.1126/sciadv.aay1344, PMID: 32494662 PMC7195188

[141] Choi Y Han H Jeon S Yoon HY Kim H Kwon IC . Deep tumor penetration of doxorubicin-loaded glycol chitosan nanoparticles using high-intensity focused ultrasound. Pharmaceutics. (2020) 12:974. doi: 10.3390/pharmaceutics12100974, PMID: 33076520 PMC7650702

[142] Zhang Q Jin H Chen L Chen Q He Y Yang Y . Effect of ultrasound combined with microbubble therapy on interstitial fluid pressure and VX2 tumor structure in rabbit. Front Pharmacol. (2019) 10:716. doi: 10.3389/fphar.2019.00716, PMID: 31293427 PMC6606793

[143] Luo J Cao J Ma G Wang X Sun Y Zhang C . Collagenase-loaded H-tiO(2) nanoparticles enhance ultrasound imaging-guided sonodynamic therapy in a pancreatic carcinoma xenograft model via digesting stromal barriers. ACS Appl Mater Interfaces. (2022) 14:40535–45. doi: 10.1021/acsami.2c08951, PMID: 36043358

[144] Gao F Wu J Niu S Sun T Li F Bai Y . Biodegradable, pH-sensitive hollow mesoporous organosilica nanoparticle (HMON) with controlled release of pirfenidone and ultrasound-target-microbubble-destruction (UTMD) for pancreatic cancer treatment. Theranostics. (2019) 9:6002–18. doi: 10.7150/thno.36135, PMID: 31534533 PMC6735371

[145] Xiao H Li X Li B Zhong Y Qin J Wang Y . Sono-promoted drug penetration and extracellular matrix modulation potentiate sonodynamic therapy of pancreatic ductal adenocarcinoma. Acta Biomater. (2023) 161:265–74. doi: 10.1016/j.actbio.2023.02.038, PMID: 36893956

[146] Wu J Sun L Liu T Dong G . Ultrasound-targeted microbubble destruction-mediated downregulation of EZH2 inhibits stemness and epithelial-mesenchymal transition of liver cancer stem cells. Onco Targets Ther. (2021) 14:221–37. doi: 10.2147/OTT.S269589, PMID: 33469303 PMC7810681

[147] Liao M Zhang Q Huang J Huang X Cheng C Tu J . Near-infrared and ultrasound triggered Pt/Pd-engineered cluster bombs for the treatment of solid tumors. J Control Release. (2024) 375:331–45. doi: 10.1016/j.jconrel.2024.09.024, PMID: 39278358

[148] Li M Liu Y Zhang Y Yu N Li J . Sono-activatable semiconducting polymer nanoreshapers multiply remodel tumor microenvironment for potent immunotherapy of orthotopic pancreatic cancer. Adv Sci (Weinh). (2023) 10:e2305150. doi: 10.1002/advs.202305150, PMID: 37870196 PMC10724419

[149] Lee HR Kim DW Jones VO Choi Y Ferry VE Geller MA . Sonosensitizer-functionalized graphene nanoribbons for adhesion blocking and sonodynamic ablation of ovarian cancer spheroids. Adv Healthc Mater. (2021) 10:e2001368. doi: 10.1002/adhm.202001368, PMID: 34050609 PMC8550295

[150] Zhu L Zhao H Zhou Z Xia Y Wang Z Ran H . Peptide-functionalized phase-transformation nanoparticles for low intensity focused ultrasound-assisted tumor imaging and therapy. Nano Lett. (2018) 18:1831–41. doi: 10.1021/acs.nanolett.7b05087, PMID: 29419305

[151] Wang XQ Li ZN Wang QM Jin HY Gao Z Jin ZH . Lipid nano-bubble combined with ultrasound for anti-keloids therapy. J Liposome Res. (2018) 28:5–13. doi: 10.1080/08982104.2016.1239633, PMID: 27733083

[152] Gao J Liu J Meng Z Li Y Hong Y Wang L . Ultrasound-assisted C(3)F(8)-filled PLGA nanobubbles for enhanced FGF21 delivery and improved prophylactic treatment of diabetic cardiomyopathy. Acta Biomater. (2021) 130:395–408. doi: 10.1016/j.actbio.2021.06.015, PMID: 34129954

[153] Liang S Lin M Wang J Wei H Huang S Yin T . A ROS/ultrasound dual-responsive nanocarrier enhances drug penetration for ameliorating metabolic dysfunction-associated steatohepatitis. Acta Biomater. (2025) 202:503–16. doi: 10.1016/j.actbio.2025.07.010, PMID: 40617493

[154] Zuo D Tan B Jia G Wu D Yu L Jia L . A treatment combined pRussian blue nanoparticles with low-intensity pulsed ultrasound alleviates cartilage damage in knee osteoarthritis by initiating PI3K/Akt/mTOR pathway. Am J Transl Res. (2021) 13:3987–4006. doi: 10.21203/rs.3.rs-77010/v1, PMID: 34149994 PMC8205753

[155] Zhang YS Ke S Hu X Wang SY Peng WQ Qian XH . Enhancing wound healing through sonodynamic silver/barium titanate heterostructures-loading gelatin/PCL nanodressings. Int J Biol Macromol. (2024) 283:137648. doi: 10.1016/j.ijbiomac.2024.137648, PMID: 39547623

[156] Gu BK Park SJ Kim MS Kang CM Kim JI Kim CH . Fabrication of sonicated chitosan nanofiber mat with enlarged porosity for use as hemostatic materials. Carbohydr Polym. (2013) 97:65–73. doi: 10.1016/j.carbpol.2013.04.060, PMID: 23769518

[157] Hou Y Yang M Li J Bi X Li G Xu J . The enhancing antifungal effect of AD1 aptamer-functionalized amphotericin B-loaded PLGA-PEG nanoparticles with a low-frequency and low-intensity ultrasound exposure on C.albicans biofilm through targeted effect. NanoImpact. (2021) 21:100275. doi: 10.1016/j.impact.2020.100275, PMID: 35559767

[158] Tang R He H Lin X Wu N Wan L Chen Q . Novel combination strategy of high intensity focused ultrasound (HIFU) and checkpoint blockade boosted by bioinspired and oxygen-supplied nanoprobe for multimodal imaging-guided cancer therapy. J Immunother Cancer. (2023) 11:e006226. doi: 10.1136/jitc-2022-006226, PMID: 36650023 PMC9853265

[159] Wooram U Hyewon K Dong Gil Y Seung Woon L Gijung K Man Kyu S . Necroptosis-inducible polymeric nanobubbles for enhanced cancer sonoimmunotherapy. Advanced Mater. (2020) 32:e1907953. doi: 10.1002/adma.201907953, PMID: 32125731

[160] Wang Y Gong F Han Z Lei H Zhou Y Cheng S . Oxygen-deficient molybdenum oxide nanosensitizers for ultrasound-enhanced cancer metalloimmunotherapy. Angew Chem Int Ed Engl. (2023) 62:e202215467. doi: 10.1002/anie.202215467, PMID: 36591974

[161] Mihlan M Wissmann S Gavrilov A Kaltenbach L Britz M Franke K . Neutrophil trapping and nexocytosis, mast cell-mediated processes for inflammatory signal relay. Cell. (2024) 187:5316–5335 e28. doi: 10.1016/j.cell.2024.07.014, PMID: 39096902

[162] Zhou Y Shen G Zhou X Li J . Therapeutic potential of tumor-associated neutrophils: dual role and phenotypic plasticity. Signal Transduct Target Ther. (2025) 10:178. doi: 10.1038/s41392-025-02242-7, PMID: 40461514 PMC12134342

[163] Adrover JM Han X Sun L Fujii T Sivetz N Dassler-Plenker J . Neutrophils drive vascular occlusion, tumour necrosis and metastasis. Nature. (2025) 645:484–495. doi: 10.1038/s41586-025-09278-3, PMID: 40670787 PMC12422981

[164] Zhu D Lu Y Yang S Hu T Tan C Liang R . PAD4 inhibitor-functionalized layered double hydroxide nanosheets for synergistic sonodynamic therapy/immunotherapy of tumor metastasis. Adv Sci (Weinh). (2024) 11:e2401064. doi: 10.1002/advs.202401064, PMID: 38708711 PMC11234469

[165] Shen J Hao J Chen Y Liu H Wu J Hu B . Neutrophil-mediated clinical nanodrug for treatment of residual tumor after focused ultrasound ablation. J Nanobiotechnology. (2021) 19:345. doi: 10.1186/s12951-021-01087-w, PMID: 34715854 PMC8555249

[166] Sun L Zhou JE Luo T Wang J Kang L Wang Y . Nanoengineered neutrophils as a cellular sonosensitizer for visual sonodynamic therapy of Malignant tumors. Adv Mater. (2022) 34:e2109969. doi: 10.1002/adma.202109969, PMID: 35174915

[167] Li Y Teng X Wang Y Yang C Yan X Li J . Neutrophil delivered hollow titania covered persistent luminescent nanosensitizer for ultrosound augmented chemo/immuno glioblastoma therapy. Adv Sci (Weinh). (2021) 8:e2004381. doi: 10.1002/advs.202004381, PMID: 34196474 PMC8425909

[168] Li Y Yang X Wei Z Niu H Wu L Chen C . Sulforaphane wrapped in self-assembled nanomicelle enhances the effect of sonodynamic therapy on glioma. Pharmaceutics. (2024) 17 34. doi: 10.3390/pharmaceutics17010034, PMID: 39861683 PMC11769538

[169] Wei J Zhong A Zhang Y Deng E Mo H Zhao H . Sivelestat-loaded neutrophil-membrane-coated antioxidative nanoparticles for targeted endothelial protection in sepsis. Pharmaceutics. (2025) 17:766. doi: 10.3390/pharmaceutics17060766, PMID: 40574078 PMC12196302

[170] Liao CC Yu HP Yang SC Alalaiwe A Dai YS Liu FC . Multifunctional lipid-based nanocarriers with antibacterial and anti-inflammatory activities for treating MRSA bacteremia in mice. J Nanobiotechnology. (2021) 19:48. doi: 10.1186/s12951-021-00789-5, PMID: 33588861 PMC7885212

[171] Nunes NS Chandran P Sundby M Visioli F da Costa Goncalves F Burks SR . Therapeutic ultrasound attenuates DSS-induced colitis through the cholinergic anti-inflammatory pathway. EBioMedicine. (2019) 45:495–510. doi: 10.1016/j.ebiom.2019.06.033, PMID: 31253515 PMC6642284

[172] Zhang S Zhang C Yan H Yang L Shi N Liu C . Sacral nerve stimulation alleviates intestinal inflammation through regulating the autophagy of macrophages and activating the inflammasome mediated by a cholinergic antiinflammatory pathway in colitis rats. Neuromodulation. (2024) 27:302–11. doi: 10.1016/j.neurom.2023.01.005, PMID: 36740464

[173] Luo Z Zheng S Hu Z Li P Zeng J Lu Y . Ultrasound-responsive taurine lipid nanoparticles attenuate oxidative stress and promote macrophage polarization for diabetic wound healing. Free Radic Biol Med. (2025) 233:302–16. doi: 10.1016/j.freeradbiomed.2025.04.007, PMID: 40187503

[174] Hu H Wang S Li Q Zhao J Pang Y Wang J . Autophagy-enhanced nanosonosensitizer mediated sonodynamic therapy for post-myocardial infarction neuromodulation and arrhythmia prevention. Theranostics. (2025) 15:2201–14. doi: 10.7150/thno.103780, PMID: 39990226 PMC11840733

[175] Yao M Jinlin L Yun Z Bing H Yun L Chaoqi L . Nanobubble-mediated co-delivery of Ce6 and miR-195 for synergized sonodynamic and checkpoint blockade combination therapy with elicitation of robust immune response in hepatocellular carcinoma. Eur J Pharmaceutics Biopharmaceutics. (2022) 181:36–48. doi: 10.1016/j.ejpb.2022.10.017, PMID: 36307001

[176] Ji C Si J Xu Y Zhang W Yang Y He X . Mitochondria-targeted and ultrasound-responsive nanoparticles for oxygen and nitric oxide codelivery to reverse immunosuppression and enhance sonodynamic therapy for immune activation. Theranostics. (2021) 11:8587–604. doi: 10.7150/thno.62572, PMID: 34373760 PMC8344010

[177] Wu N Zhang Q Tang R Deng L Cao Y Fu B . Ultrasound visualization of spatiotemporal autophagy-regulated nanodroplets for amplifying ICB in melanoma via remodeling tumor inflammatory microenvironment. ACS Appl Mater Interfaces. (2025) 17:29364–78. doi: 10.1021/acsami.5c03394, PMID: 40331917 PMC12100593

[178] Wang Y Li H Niu G Li Y Huang Z Cheng S . Boosting sono-immunotherapy of prostate carcinoma through amplifying domino-effect of mitochondrial oxidative stress using biodegradable cascade-targeting nanocomposites. ACS Nano. (2024) 18:5828–46. doi: 10.1021/acsnano.3c12511, PMID: 38332473

[179] Chen H Liu L Ma A Yin T Chen Z Liang R . Noninvasively immunogenic sonodynamic therapy with manganese protoporphyrin liposomes against triple-negative breast cancer. Biomaterials. (2021) 269:120639. doi: 10.1016/j.biomaterials.2020.120639, PMID: 33434714

[180] Cheng W Yang J Pan Y Qu H Duan Z Wu J . Noninvasive activation of local and systemic immunity with a sequential-targeting sonodynamic nanovaccine to treat glioblastoma. ACS Nano. (2025) 19:27804–27824. doi: 10.1021/acsnano.5c08928, PMID: 40698543

[181] Ya Z Wu H Jia W Chu H Zhu M Li Y . Whirlwind acoustic vortex-regulated intelligent microbots for enhanced uniform drug bioaccumulation to boost systemic immunity against large tumors. Small. (2025) 21:e2503639. doi: 10.1002/smll.202503639, PMID: 40484802

[182] Guo J Pan X Wu Q Li P Wang C Liu S . Bio-barrier-adaptable biomimetic nanomedicines combined with ultrasound for enhanced cancer therapy. Signal Transduct Target Ther. (2025) 10:137. doi: 10.1038/s41392-025-02217-8, PMID: 40274835 PMC12022184

[183] Glickstein B Bismuth M Gattegno R Bercovici T Shaul O Aronovich R . Volumetric nanodroplet-enhanced ultrasound surgery combined with immune checkpoint inhibition as a cancer therapy platform. Small. (2025) 21:e2411474. doi: 10.1002/smll.202411474, PMID: 40059532 PMC12160685

[184] Li M Li Y Zheng J Ma Z Zhang J Wu H . Ultrasound-responsive nanocarriers with siRNA and Fe(3)O(4) regulate macrophage polarization and phagocytosis for augmented non-small cell lung cancer immunotherapy. J Nanobiotechnology. (2024) 22:605. doi: 10.1186/s12951-024-02883-w, PMID: 39375761 PMC11460142

[185] Yin C Wang G Zhang Q Li Z Dong T Li Q . Ultrasound nanodroplets loaded with Siglec-G siRNA and Fe(3)O(4) activate macrophages and enhance phagocytosis for immunotherapy of triple-negative breast cancer. J Nanobiotechnology. (2024) 22:773. doi: 10.1186/s12951-024-03051-w, PMID: 39696453 PMC11658085

[186] Xu S Meng L Hu Q Li F Zhang J Kong N . Closed-loop control of macrophage engineering enabled by focused-ultrasound responsive mechanoluminescence nanoplatform for precise cancer immunotherapy. Small. (2024) 20:e2401398. doi: 10.1002/smll.202401398, PMID: 39101277

[187] Chen S Li Y Zhou Z Saiding Q Zhang Y An S . Macrophage hitchhiking nanomedicine for enhanced beta-elemene delivery and tumor therapy. Sci Adv. (2025) 11:eadw7191. doi: 10.1126/sciadv.adw7191, PMID: 40397726 PMC12094207

[188] Shen Y Yu N Zhao W Niu S Qiu P Zeng H . M1-macrophage membrane-camouflaged nanoframeworks activate multiple immunity via calcium overload and photo-sonosensitization. Biomaterials. (2025) 320:123287. doi: 10.1016/j.biomaterials.2025.123287, PMID: 40147112

[189] Kang Y She Y Zang Y Yuan M Niu G Tian X . Biohybrid microrobot enteric-coated microcapsule for oral treatment of colorectal cancer. Adv Mater. (2025), 37 e20586. doi: 10.1002/adma.202420586, PMID: 40679042

[190] Zhang J Wang P Meng L Li X Li F Kong N . Focused ultrasound-responsive artificial killer cells for enhanced closed-loop cancer immunotherapy. Mater Horiz. (2025) 12:8134–8146. doi: 10.1039/d5mh00644a, PMID: 40635646

[191] Wang L Zhang C Zhao J Zhu Z Wang J Fan W . Biomimetic targeting nanoadjuvants for sonodynamic and chronological multi-immunotherapy against holistic biofilm-related infections. Adv Mater. (2024) 36:e2308110. doi: 10.1002/adma.202308110, PMID: 38088059

[192] Hou L Liu Q Shen L Liu Y Zhang X Chen F . Nano-delivery of fraxinellone remodels tumor microenvironment and facilitates therapeutic vaccination in desmoplastic melanoma. Theranostics. (2018) 8:3781–96. doi: 10.7150/thno.24821, PMID: 30083259 PMC6071534

[193] Xie X Zhang J Wang Y Shi W Tang R Tang Q . Nanomaterials augmented bioeffects of ultrasound in cancer immunotherapy. Mater Today Bio. (2024) 24:100926. doi: 10.1016/j.mtbio.2023.100926, PMID: 38179429 PMC10765306

[194] Salgado M Sepulveda-Arriagada V Konar-Nie M Garcia-Robles MA Saez JC . Poly (I:C)-induced inflammation requires the activation of toll-like receptor 3/Ca(2+)/CaMKII/pannexin 1-dependent signaling. Theranostics. (2025) 15:2470–86. doi: 10.7150/thno.100687, PMID: 39990207 PMC11840745

[195] Saotome K Murthy SE Kefauver JM Whitwam T Patapoutian A Ward AB . Structure of the mechanically activated ion channel Piezo1. Nature. (2018) 554:481–6. doi: 10.1038/nature25453, PMID: 29261642 PMC6010196

[196] Hope JM Dombroski JA Pereles RS Lopez-Cavestany M Greenlee JD Schwager SC . Fluid shear stress enhances T cell activation through Piezo1. BMC Biol. (2022) 20:61. doi: 10.1186/s12915-022-01266-7, PMID: 35260156 PMC8904069

[197] Li M Zhang Y Jiang W Li S Lin X Ma M . ID1 boosts antiviral immunity by countering PRMT5-mediated STING methylation. Cell Rep. (2025) 44:116547. doi: 10.1016/j.celrep.2025.116547, PMID: 41205173

[198] Sethuraman SN Singh MP Patil G Li S Fiering S Hoopes PJ . Novel calreticulin-nanoparticle in combination with focused ultrasound induces immunogenic cell death in melanoma to enhance antitumor immunity. Theranostics. (2020) 10:3397–412. doi: 10.7150/thno.42243, PMID: 32206098 PMC7069083

[199] Engelen Y Krysko DV Effimova I Breckpot K Versluis M De Smedt S . Optimizing high-intensity focused ultrasound-induced immunogenic cell-death using passive cavitation mapping as a monitoring tool. J Control Release. (2024) 375:389–403. doi: 10.1016/j.jconrel.2024.09.016, PMID: 39293525

[200] Sheybani ND Batts AJ Mathew AS Thim EA Price RJ . Focused ultrasound hyperthermia augments release of glioma-derived extracellular vesicles with differential immunomodulatory capacity. Theranostics. (2020) 10:7436–47. doi: 10.7150/thno.46534, PMID: 32642004 PMC7330848

[201] Yang K Xiao Z He X Weng R Zhao X Sun T . Mechanisms of pannexin 1 (PANX1) channel mechanosensitivity and its pathological roles. Int J Mol Sci. (2022) 23:1523. doi: 10.3390/ijms23031523, PMID: 35163442 PMC8836264

[202] Mackey C Feng Y Liang C Liang A Tian H Narayan OP . Mechanical modulation, physiological roles, and imaging innovations of intercellular calcium waves in living systems. Cancers (Basel). (2025) 17:1851. doi: 10.3390/cancers17111851, PMID: 40507332 PMC12153901

[203] Maciulevicius M Navickaite D Chopra S Jakstys B Satkauskas S . Sudden cell death induced by ca(2+) delivery via microbubble cavitation. Biomedicines. (2021) 9:32. doi: 10.3390/biomedicines9010032, PMID: 33406593 PMC7823641

[204] Sun C Dong Y Wei J Cai M Liang D Fu Y . Acoustically accelerated neural differentiation of human embryonic stem cells. Acta Biomater. (2022) 151:333–45. doi: 10.1016/j.actbio.2022.07.041, PMID: 35914692

[205] Araujo Martins Y Zeferino Pavan T Fonseca Vianna Lopez R . Sonodynamic therapy: Ultrasound parameters and *in vitro* experimental configurations. Int J Pharm. (2021) 610:121243. doi: 10.1016/j.ijpharm.2021.121243, PMID: 34743959

[206] Abramowicz JS . Biosafety of sonography: still a mystery to most obstetrics (and other) providers. J Ultrasound Med. (2020) 39:1683–5. doi: 10.1002/jum.15272, PMID: 32277498

[207] Hopewell S Chan AW Collins GS Hrobjartsson A Moher D Schulz KF . CONSORT 2025 statement: updated guideline for reporting randomized trials. JAMA. (2025) 333:1998–2005. doi: 10.1001/jama.2025.4347, PMID: 40228499

[208] Stuart T Satija R . Integrative single-cell analysis. Nat Rev Genet. (2019) 20:257–72. doi: 10.1038/s41576-019-0093-7, PMID: 30696980

[209] Wu L Huang C Emery BP Sedgwick AC Bull SD He XP . Forster resonance energy transfer (FRET)-based small-molecule sensors and imaging agents. Chem Soc Rev. (2020) 49:5110–39. doi: 10.1039/c9cs00318e, PMID: 32697225 PMC7408345

[210] Huang Z Kanchanawong P . Ultra high-speed single-molecule fluorescence imaging. J Cell Biol. (2023) 222:e202306136. doi: 10.1083/jcb.202306136, PMID: 37458726 PMC10351246

[211] Pajic-Lijakovic I Eftimie R Milivojevic M Bordas SPA . Multi-scale nature of the tissue surface tension: Theoretical consideration on tissue model systems. Adv Colloid Interface Sci. (2023) 315:102902. doi: 10.1016/j.cis.2023.102902, PMID: 37086625

[212] Walpole J Papin JA Peirce SM . Multiscale computational models of complex biological systems. Annu Rev BioMed Eng. (2013) 15:137–54. doi: 10.1146/annurev-bioeng-071811-150104, PMID: 23642247 PMC3970111

[213] Shaw A ter Haar G Haller J Wilkens V . Towards a dosimetric framework for therapeutic ultrasound. Int J Hyperthermia. (2015) 31:182–92. doi: 10.3109/02656736.2014.997311, PMID: 25774889

[214] Bader KB Padilla F Haworth KJ Ellens N Dalecki D Miller DL . Bioeffects committee of the american institute of ultrasound in: overview of therapeutic ultrasound applications and safety considerations: 2024 update. J Ultrasound Med. (2025) 44:381–433. doi: 10.1002/jum.16611, PMID: 39526313 PMC11796337

[215] McDannold N Livingstone M Top CB Sutton J Todd N Vykhodtseva N . Preclinical evaluation of a low-frequency transcranial MRI-guided focused ultrasound system in a primate model. Phys Med Biol. (2016) 61:7664–87. doi: 10.1088/0031-9155/61/21/7664, PMID: 27740941 PMC5079272

[216] Maciulevicius M Tamosiunas M Venslauskas MS Satkauskas S . The relation of bleomycin delivery efficiency to microbubble sonodestruction and cavitation spectral characteristics. Sci Rep. (2020) 10:7743. doi: 10.1038/s41598-020-64213-y, PMID: 32385397 PMC7210292

[217] Song KH Fan AC Hinkle JJ Newman J Borden MA Harvey BK . Microbubble gas volume: A unifying dose parameter in blood-brain barrier opening by focused ultrasound. Theranostics. (2017) 7:144–52. doi: 10.7150/thno.15987, PMID: 28042323 PMC5196892

[218] Cao X Cao J Xu T Zheng L Dai J Zhang X . Construction of nanodelivery system based on the interaction mechanism between ultrasound-treated soybean whey protein and quercetin: structure, physicochemical stability and bioaccessibility. Ultrason Sonochem. (2025) 112:107195. doi: 10.1016/j.ultsonch.2024.107195, PMID: 39671813 PMC11700283

[219] Darmani G Bergmann TO Butts Pauly K Caskey CF de Lecea L Fomenko A . Non-invasive transcranial ultrasound stimulation for neuromodulation. Clin Neurophysiol. (2022) 135:51–73. doi: 10.1016/j.clinph.2021.12.010, PMID: 35033772

[220] Maciulevicius M Raisutis R Jakstys B Svilainis L Chaziachmetovas A Satkauskas S . The assessment of calcium and bleomycin cytotoxic efficiency in relation to cavitation dosimetry. Pharmaceutics. (2023) 15:1463. doi: 10.3390/pharmaceutics15051463, PMID: 37242705 PMC10221366

[221] Meyer D Rusho RZ Alam W Christensen GE Howard DM Atha J . High-resolution three-dimensional hybrid MRI + Low dose CT vocal tract modeling: A cadaveric pilot study. J Voice. (2025) 39:963–70. doi: 10.1016/j.jvoice.2022.09.013, PMID: 40804735

[222] Saratkar SY Langote M Kumar P Gote P Weerarathna IN Mishra GV . Digital twin for personalized medicine development. Front Digit Health. (2025) 7:1583466. doi: 10.3389/fdgth.2025.1583466, PMID: 40851640 PMC12369496

[223] Vallee A . Envisioning the future of personalized medicine: role and realities of digital twins. J Med Internet Res. (2024) 26:e50204. doi: 10.2196/50204, PMID: 38739913 PMC11130780

[224] Martinez P Bottenus N Borden M . Cavitation characterization of size-isolated microbubbles in a vessel phantom using focused ultrasound. Pharmaceutics. (2022) 14:2246. doi: 10.3390/pharmaceutics14091925, PMID: 36145673 PMC9501432

[225] Sun T Zhang Y Power C Alexander PM Sutton JT Aryal M . Closed-loop control of targeted ultrasound drug delivery across the blood-brain/tumor barriers in a rat glioma model. Proc Natl Acad Sci U.S.A. (2017) 114:E10281–90. doi: 10.1073/pnas.1713328114, PMID: 29133392 PMC5715774

[226] Dezsi L Meszaros T Kozma G Olah H. V. M, C. Z. Szabo M Patko Z . A naturally hypersensitive porcine model may help understand the mechanism of COVID-19 mRNA vaccine-induced rare (pseudo) allergic reactions: complement activation as a possible contributing factor. Geroscience. (2022) 44:597–618. doi: 10.1007/s11357-021-00495-y, PMID: 35146583 PMC8831099

[227] Szebeni J . Evaluation of the acute anaphylactoid reactogenicity of nanoparticle-containing medicines and vaccines using the porcine CARPA model. Methods Mol Biol. (2024) 2789:229–43. doi: 10.1007/978-1-0716-3786-9_23, PMID: 38507008

[228] Downs ME Buch A Sierra C Karakatsani ME Teichert T Chen S . Long-term safety of repeated blood-brain barrier opening via focused ultrasound with microbubbles in non-human primates performing a cognitive task. PloS One. (2015) 10:e0125911. doi: 10.1371/journal.pone.0125911, PMID: 25945493 PMC4422704

[229] Novell A Kamimura HAS Cafarelli A Gerstenmayer M Flament J Valette J . A new safety index based on intrapulse monitoring of ultra-harmonic cavitation during ultrasound-induced blood-brain barrier opening procedures. Sci Rep. (2020) 10:10088. doi: 10.1038/s41598-020-66994-8, PMID: 32572103 PMC7308405

[230] Church CC Carstensen EL . Stable” inertial cavitation. Ultrasound Med Biol. (2001) 27:1435–7. doi: 10.1016/s0301-5629(01)00441-0, PMID: 11731057

[231] Gandhi K Barzegar-Fallah A Banstola A Rizwan SB Reynolds JNJ . Ultrasound-mediated blood-brain barrier disruption for drug delivery: A systematic review of protocols, efficacy, and safety outcomes from preclinical and clinical studies. Pharmaceutics. (2022) 14:833. doi: 10.3390/pharmaceutics14040833, PMID: 35456667 PMC9029131

[232] Angolano C Hansen E Ajjawi H Nowlin P Zhang Y Thunemann N . Characterization of focused ultrasound blood-brain barrier disruption effect on inflammation as a function of treatment parameters. BioMed Pharmacother. (2025) 182:117762. doi: 10.1016/j.biopha.2024.117762, PMID: 39719739 PMC11803570

[233] Corbo C Molinaro R Parodi A Toledano Furman NE Salvatore F Tasciotti E . The impact of nanoparticle protein corona on cytotoxicity, immunotoxicity and target drug delivery. Nanomed (Lond). (2016) 11:81–100. doi: 10.2217/nnm.15.188, PMID: 26653875 PMC4910943

[234] Rampado R Crotti S Caliceti P Pucciarelli S Agostini M . Recent advances in understanding the protein corona of nanoparticles and in the formulation of “Stealthy” Nanomaterials. Front Bioeng Biotechnol. (2020) 8:166. doi: 10.3389/fbioe.2020.00166, PMID: 32309278 PMC7145938

[235] Szebeni J Storm G Ljubimova JY Castells M Phillips EJ Turjeman K . Applying lessons learned from nanomedicines to understand rare hypersensitivity reactions to mRNA-based SARS-CoV-2 vaccines. Nat Nanotechnol. (2022) 17:337–46. doi: 10.1038/s41565-022-01071-x, PMID: 35393599

[236] Wang Z Li P Zeng X Guo J Zhang C Fan Z . CAR-T therapy dilemma and innovative design strategies for next generation. Cell Death Dis. (2025) 16:211. doi: 10.1038/s41419-025-07454-x, PMID: 40148310 PMC11950394

[237] Zhang M Liu C Tu J Tang M Ashrafizadeh M Nabavi N . Advances in cancer immunotherapy: historical perspectives, current developments, and future directions. Mol Cancer. (2025) 24:136. doi: 10.1186/s12943-025-02305-x, PMID: 40336045 PMC12057291

[238] Chen P Zhang P Shah NH Cui Y Wang Y . A comprehensive review of inorganic sonosensitizers for sonodynamic therapy. Int J Mol Sci. (2023) 24:12001. doi: 10.3390/ijms241512001, PMID: 37569377 PMC10418994

[239] Li D Zhang R Lan H Chen M Huang Z Zhao H . A retrospective study on adverse events of intravenous administration of sulfur hexafluoride microbubbles in abdominal and superficial applications in 83,778 patients. Insights Imaging. (2024) 15:65. doi: 10.1186/s13244-024-01632-9, PMID: 38411872 PMC10899544

[240] Umme S Siciliano G Primiceri E Turco A Tarantini I Ferrara F . Electrochemical sensors for liquid biopsy and their integration into lab-on-chip platforms: revolutionizing the approach to diseases. Chemosensors. (2023) 11:517. doi: 10.3390/chemosensors11100517

[241] Izadifar Z Izadifar Z Chapman D Babyn P . An introduction to high intensity focused ultrasound: systematic review on principles, devices, and clinical applications. J Clin Med. (2020) 9:460. doi: 10.3390/jcm9020460, PMID: 32046072 PMC7073974

[242] Ashar H Ranjan A . Immunomodulation and targeted drug delivery with high intensity focused ultrasound (HIFU): Principles and mechanisms. Pharmacol Ther. (2023) 244:108393. doi: 10.1016/j.pharmthera.2023.108393, PMID: 36965581 PMC12323623

[243] Gong Z Dai Z . Design and challenges of sonodynamic therapy system for cancer theranostics: from equipment to sensitizers. Adv Sci (Weinh). (2021) 8:2002178. doi: 10.1002/advs.202002178, PMID: 34026428 PMC8132157

[244] Golombek SK May JN Theek B Appold L Drude N Kiessling F . Tumor targeting via EPR: Strategies to enhance patient responses. Adv Drug Delivery Rev. (2018) 130:17–38. doi: 10.1016/j.addr.2018.07.007, PMID: 30009886 PMC6130746

[245] Hua S de Matos MBC Metselaar JM Storm G . Current trends and challenges in the clinical translation of nanoparticulate nanomedicines: pathways for translational development and commercialization. Front Pharmacol. (2018) 9:790. doi: 10.3389/fphar.2018.00790, PMID: 30065653 PMC6056679

[246] Zhu G Lynn GM Jacobson O Chen K Liu Y Zhang H . Albumin/vaccine nanocomplexes that assemble *in vivo* for combination cancer immunotherapy. Nat Commun. (2017) 8:1954. doi: 10.1038/s41467-017-02191-y, PMID: 29203865 PMC5715147

[247] Entzian K Aigner A . Drug delivery by ultrasound-responsive nanocarriers for cancer treatment. Pharmaceutics. (2021) 13:1135. doi: 10.3390/pharmaceutics13081135, PMID: 34452096 PMC8397943

